# Radiation dose escalation in locally advanced oesophageal cancer: a systematic review and hierarchical Bayesian meta-analysis

**DOI:** 10.1016/j.eclinm.2025.103432

**Published:** 2025-08-13

**Authors:** Yang-Hong Dai, Po-Huang Chen, Yi-Jhih Huang, Ding-Jie Lee, Po-Chien Shen, Cheng-Hsiang Lo, Yu-Guang Chen, Yu-Fu Su, Jen-Fu Yang, Ying-Fu Wang, Wen-Yen Huang, Chun-Shu Lin, Chih-Cheng Tsao, Katherine A. Vallis

**Affiliations:** a Department of Oncology, University of Oxford, Oxford, UK; b Department of Radiation Oncology, Tri-Service General Hospital, National Defense Medical University, Taipei, Taiwan; c Division of Haematology and Oncology, Department of Internal Medicine, Tri-Service General Hospital, National Defense Medical University, Taipei, Taiwan; d Division of Thoracic Surgery, Department of Surgery, Tri-Service General Hospital, National Defense Medical University, Taipei, Taiwan; e Division of Nephrology, Department of Internal Medicine, Tri-Service General Hospital Keelung Branch, National Defense Medical University, Taiwan; f Department of Biological Science and Technology, Institute of Bioinformatics and System Biology, National Yang Ming Chiao Tung University, Hsinchu, Taiwan; g Department of Radiation Oncology, Taitung MacKay Memorial Hospital, Taitung, Taiwan

**Keywords:** Oesophageal cancer, Radiation therapy, Dose escalation, Bayesian meta-analysis, Chemoradiotherapy, Survival outcomes, Squamous cell carcinoma

## Abstract

**Background:**

The impact of radiation dose escalation in locally advanced oesophageal cancer remains controversial. While higher doses may improve locoregional control, their effect on overall survival (OS) and progression-free survival (PFS) remains uncertain. The aim of this study was to thoroughly evaluate the impact of dose escalation on survival.

**Methods:**

A systematic search on Jan 4, 2025, identified studies evaluating definitive chemoradiotherapy (CRTx) for locally advanced oesophageal cancer stratified by radiation dose. Bayesian and frequentist meta-analyses assessed OS, PFS, and locoregional PFS (LRPFS). Meta-regression examined the influence of histology, tumour location, chemotherapy, and radiation techniques. Additional analyses included gene set enrichment analysis and immune infiltration estimation. This study is registered with PROSPERO, CRD42024538961.

**Findings:**

A total of 42 studies involving 8379 patients were included. High-dose radiotherapy significantly improved LRPFS, with the largest benefit observed at 1-year (median difference: 18.6%; 95% credible interval [CrI]: 10.7–26.1%). A modest improvement in OS was noted at 3-year (7.0%; 95% CrI: 0.01–13.9%), particularly in squamous cell carcinoma (SqCC). Meta-regression identified SqCC and taxane-based chemotherapy as key moderators, with high-dose conferring greater benefits in Asian populations. Genomic analysis revealed higher radiosensitivity and significant immune activation in Asian SqCC. Taxane-based chemotherapy regimens were the strongest predictors of 1-/2-year OS and PFS but diminished at 5-year. Any-grade pneumonitis was more common in high-dose, but frequencies of grade 3 or higher pneumonitis were similar. Modern techniques like intensity-modulated or volumetric-modulated radiotherapy were associated with higher complete response rate and a trend toward reduced toxicity. The study heterogeneity was moderate to high across pooled estimates but addressed through hierarchical modeling and subgroup/sensitivity analyses. Most included studies were retrospective with moderate risk of bias, and the certainty of evidence for primary outcomes was rated as low to high.

**Interpretation:**

Radiation dose escalation improves locoregional control and may enhance OS in SqCC, particularly in Asian populations, highlighting the need for histology- and region-specific therapeutic strategies. The choice of chemotherapy regimen may affect the interpretation of survival effects associated with high-dose. Furthermore, the genomic correlates of radiosensitivity and immune activation suggest potential for biologically guided dose personalization and combination with immunotherapy.

**Funding:**

None.

**Research in context**

**Evidence before this study**

We systematically searched PubMed, Embase, MEDLINE, Web of Science, and the Cochrane Library from database inception to Jan 4, 2025 using search terms related to oesophageal cancer, radiation dose escalation, and chemoradiotherapy. Studies were included if they reported survival or disease control outcomes for patients with locally advanced oesophageal cancer receiving definitive chemoradiotherapy stratified by radiation dose. Prior randomized trials such as INT 0123 and ARTDECO failed to show a survival benefit with higher doses, though they were limited by older techniques and heterogeneous patient populations. Previous meta-analyses have been inconsistent, often lacking adjustment for clinical confounders, histology, and geographic variability, and none incorporated both Bayesian and frequentist methods. No prior study integrated genomic or immune landscape data to explore population-specific radiosensitivity.

**Added value of this study**

This is the first meta-analysis to apply a hierarchical Bayesian framework alongside frequentist methods to evaluate the impact of radiation dose escalation in locally advanced oesophageal cancer, drawing from 42 studies and 8379 patients. Our findings show that high-dose radiotherapy (BED ≥70 Gy) improves LRPFS and provides modest survival benefits in SqCC, especially among Asian populations. Through meta-regression, SqCC histology and taxane-based chemotherapy emerged as key effect modifiers. Genomic analysis revealed lower radiosensitivity indices and greater immune activation in Asian SqCC, potentially explaining enhanced treatment responses. Integration of modern radiation techniques such as IMRT and VMAT was associated with higher complete response rates and a trend toward reduced toxicity.

**Implications of all the available evidence**

Radiation dose escalation improves locoregional control and may offer overall survival benefits in select subgroups of locally advanced oesophageal cancer, particularly Asian patients with SqCC. The results challenge the one-size-fits-all approach and support histology- and geography-specific treatment strategies. Genomic and immune differences between populations highlight the need for personalized radiotherapy protocols. Future prospective trials should incorporate molecular profiling, stratified randomization by region and histology, and modern radiation techniques to optimize treatment efficacy and minimize toxicity.

## Introduction

Oesophageal cancer ranks among the most aggressive malignancies worldwide, with over 0.6 million new cases and 0.54 million deaths reported in 2020.^1^ By 2040, its global incidence is projected to exceed 1 million annual cases, driven disproportionately by squamous cell carcinoma (SqCC) in Eastern Asia and sub-Saharan Africa and rising rates of adenocarcinoma in Western nations, where obesity and gastroesophageal reflux disease are key etiological factors.^2^ Despite advancements in multimodal therapy, prognosis remains dismal, with 5-year survival rates stagnating at 20% in the U.S. and even lower in resource-limited regions.^1^^,^^3^

The therapeutic paradigm for locally advanced oesophageal cancer shifted in the 1990s with the landmark RTOG 85-01 trial, which established concurrent chemoradiotherapy (CRTx) as the cornerstone of definitive management.^4^ However, locoregional failure persists in 40–60% of cases, underscoring the limitations of standard-dose radiotherapy (50.4 Gray [Gy]).^5^ Emerging data suggest that tumour hypoxia, anatomical constraints, and intrinsic radioresistance in SqCC may contribute to these suboptimal outcomes.^6^^,^^7^

Clinical translation, however, has proven contentious. The phase III INT 0123 trial found no survival benefit with 64.8 Gy versus 50.4 Gy, though critics highlight suboptimal radiation techniques and toxicity management in trial design.^8^ Subsequent studies leveraging intensity-modulated radiotherapy (IMRT), such as the Dutch ARTDECO trial, escalated doses to 61.6 Gy via simultaneous integrated boost but similarly reported no improvement in locoregional progression-free survival (LRPFS), overall survival (OS) and progression-free survival (PFS) compared to the SD arm.^9^ These null findings contrast with dose-escalation successes in non-small cell lung cancer (NSCLC), where stereotactic regimens (63–103 Gy) achieve superior local control,^10^ raising questions about oesophageal cancer-specific radiobiological barriers.

Given the uncertainties of dose escalation in oesophageal cancer treatment, our research focused on verifying the relationship between radiation dose and therapeutic efficacy and identifying potential clinical modulators. We conducted a meta-analysis that incorporated both frequentist and Bayesian approaches, integrating data from multiple cohort studies to assess whether higher radiation doses correlate with improved survival outcomes. Our study combined results from varied clinical environments and locations, encompassing a broad range of patient demographics, treatment methods, and disease stages. This comprehensive approach aimed to address the limitations of individual studies, offering a more detailed understanding of the dose-response dynamics in oesophageal cancer therapy.

## Methods

### Search strategy and selection criteria

Our research followed the Preferred Reporting Items for Systematic Reviews and Meta-Analyses (PRISMA) guidelines.^11^ On Jan 4, 2025, we conducted a comprehensive literature search across multiple databases, including PubMed MEDLINE, EMBASE, the Cochrane Library, Web of Science, and Scopus. The search focused on studies investigating radiotherapy as part of definitive treatment for localized oesophageal cancer in combination with systemic therapies. To enhance search precision, we utilized controlled vocabulary terms such as Medical Subject Headings (MeSH). No restrictions were applied regarding language or publication date. The study protocol was registered with PROSPERO (registration number: CRD42024538961). The detailed search strategy is provided in Supplementary Table S1.

We included both prospective and retrospective cohort studies involving patients with oesophageal cancer who received curative treatment that included radiation therapy. Eligible studies were required to provide detailed information on patient characteristics, radiation dosages (including total dose and/or number of fractions), and survival outcomes. Studies were excluded if they reported a wide range of radiation doses without clear stratification, included fewer than 30 patients, or were non-original research articles such as editorials, commentaries, or letters.

Data extraction was conducted with discrepancies resolved through consensus. We extracted the following information: authors, publication year, country, number of patients, and patient demographics such as age, gender, Eastern Cooperative Oncology Group (ECOG) performance status, histological type, tumour location, clinical stage, and the edition of staging system used (the American Joint Committee on Cancer). Treatment details included radiotherapy techniques, radiation doses, fraction sizes, and chemotherapy regimens. The primary outcomes extracted included OS, PFS, and LRPFS at 1, 2, 3, and 5 years. For LRPFS, we also included studies reporting local progression-free survival (LPFS) where regional recurrence data were not separately available, recognizing that LPFS captures a key component of locoregional control. This approach allowed for broader inclusion of studies with similar clinical intent. Secondary outcomes encompassed treatment failure rates, response rates, and reported toxicities. The quality assessment of randomized controlled trials (RCTs) was performed using the Cochrane Risk of Bias 2 (RoB 2) tool, while observational cohort studies were evaluated using the Newcastle-Ottawa Scale (NOS).^12^^,^^13^ Grading of Recommendations Assessment, Development, and Evaluation (GRADE) was used to evaluate quality of evidence and making practice recommendations.^14^ All assessments were conducted independently by three authors (Y.H.D., P.H.C., and Y.J.H.).

### Ethics

Ethics committee review and/or approval was not required for this study, as it was a systematic review and meta-analysis based exclusively on data from previously published literature.

### Data analysis

Survival rates and the number of patients at risk were collected at predefined time points (1, 2, 3, and 5 years). For studies that did not report a risk table, the number of patients at risk for each subsequent time point was estimated by multiplying the total number of patients by the reported survival rate at the corresponding time point.

Survival rate differences in oesophageal cancer are influenced by multiple confounders, limiting the reliability of proportional meta-analysis (PMA), which cannot adjust for study-level differences.^15^ To address this, a Bayesian meta-analytic framework was employed to refine PMA estimates by incorporating prior distributions and adjusting for key clinical factors. This approach integrates prior knowledge with observed data, producing posterior estimates that enhance accuracy and interpretability.^16^

PMA was conducted using R (version 4.3.2) and *metaprop* function, applying the inverse variance method with logit transformation under a random-effects model. Subgroup analyses were performed based on radiation dose group—defined by biologically effective dose (BED ≥70 Gy) using an α/β ratio of 10 Gy—as well as chemotherapy regimens and key patient characteristics, including median age, male sex, ECOG ≥1, tumour length, histology (SqCC), tumour location, T3–T4 stage, and nodal positivity.^17^ Data missingness was handled via multiple imputation (m = 5) using the *mice* package.

Bayesian meta-analyses (BMA) for survival rate differences were conducted using the *brms* package in R, with priors informed by PMA and study-level factors to account for heterogeneity. Prior means were based on estimated proportional differences, with standard deviations derived from 95% confidence intervals (CIs). This strategy ensures that prior distributions are evidence-informed rather than subjective or overly diffuse, thereby maintaining clinical plausibility and interpretability. At the same time, heterogeneity between studies was modeled using weakly informative priors (e.g., half-Cauchy for random effects), allowing the data to drive inference while retaining flexibility to accommodate between-study variation. This approach is conceptually aligned with the commensurate prior framework described by Hobbs et al., which emphasizes data-adaptive borrowing of strength from historical or subgroup data based on similarity, and explicitly incorporates uncertainty in between-study differences.^18^ The integration of PMA-informed priors within a hierarchical Bayesian structure ensures that prior knowledge is meaningfully incorporated without compromising the model’s flexibility or reliability.

Models assumed a binomial distribution with a logit link function and were estimated via four Markov Chain Monte Carlo chains (2000 iterations, 1000 warm-up), adapt_delta = 0.99, and tree depth = 15 for improved convergence. Convergence was confirmed with R-hat values <1.01 for all parameters, and no divergent transitions were observed. Computations were parallelized across four cores. Posterior distributions for radiation dose effects were visualized using bayesplot. When patient counts were estimated rather than reported, a correction factor (weight = 0.8) was applied to account for uncertainty. Bayes Factors (BFs), computed via Savage-Dickey density ratios using *bayestestR* package, quantified evidence against the null (BF >3 = moderate, BF >10 = strong evidence).^19^ To demonstrate the robustness of our analysis, sensitivity analysis was performed for regions (China versus non-China), study designs (prospective versus retrospective) and number at risk for survival analysis (reported number at risk versus estimated number at risk). To quantify between-study heterogeneity in the BMA models, we incorporated study-specific random intercepts grouped by study as hierarchical effects. The standard deviation of these random intercepts, often denoted as τ, represents the magnitude of between-study heterogeneity on the log-odds scale.

Meta-regression analyses were performed using the *metafor* package to identify potential moderators influencing the effect of radiation dose on survival outcomes. Significant moderators identified through meta-regression were subsequently used as subgroups for BMA. Significant moderators were visualized via heatmap using *ComplexHeatmap* package.

Subgroup PMA was conducted to compare high-dose versus standard-dose radiation groups with respect to any failure, locoregional failure, and distant failure. Similar analyses assessed treatment response, including partial plus complete response (PR + CR) rates and complete response (CR) rates. For toxicity outcomes, data were collected on both any-grade toxicity and ≥grade 3 toxicity, as defined by the Common Terminology Criteria for Adverse Events (CTCAE). Additionally, radiation techniques (2D/3D CRT versus IMRT) were analyzed to evaluate the impact of high-dose.

To address potential non-linear interactions between chemotherapy regimens and radiation dose, we employed MetaForest, a random forest–based meta-analytic method that accounts for study-level heterogeneity and complex predictor dependencies.^20^ Using the *MetaForest* package with a random-effects weighted model (5000 trees), we first constructed two models: a treatment-only model (including radiation dose group and chemotherapy regimens), and a full model that additionally incorporated key patient characteristics (e.g., age, sex, ECOG ≥1, T3–T4 stage, and nodal positivity) selected based on prior meta-regression findings. We evaluated model performance using out-of-bag R^2^ (OOB-R^2^) and assessed improvement in predictive accuracy through a paired t-test comparing predicted values from the two models. Patient characteristics with minimal or no contribution to predictive performance were excluded. Finally, we applied recursive feature elimination (100 replications) to identify variables with meaningful contributions to model performance. Partial dependence plots visualized the marginal effects of chemotherapy selection and radiation dose on survival outcomes.

To investigate potential biological explanations for the observed regional differences in radiotherapy response, we evaluated radiosensitivity using the RSI, a validated gene expression-based predictor of intrinsic tumour radioresistance. The RSI was calculated to compare SqCC radiosensitivity between Asian and Western populations.^21^ Gene expression data were sourced from Gene Expression Omnibus (GEO) (GSE53624, GSE225178) for Asian cohorts (China and Japan) and TCGA-ESCA (via *UCSCXenaTools* package) for Western populations.^22^^,^^23^ GEO data were normalized per author-defined methods, retaining probes with the highest variation, while TCGA data were processed using RNA-Seq by Expectation-Maximization expected counts and normalized via *limma*.^24^ RSI was computed using a predefined formula based on 10 key genes, with higher RSI indicating greater radioresistance (Supplementary Table S2).

To further assess the difference in predicted response to CRTx between Asian and Western populations, we performed single-sample Gene Set Enrichment Analysis (ssGSEA) using the *GSVA* package. Pathway enrichment scores were computed for four predefined gene sets related to treatment response (Supplementary Table S3).^25^, ^26^, ^27^, ^28^

Given that immune cell infiltration has been implicated in modulating radiosensitivity, we hypothesized that differences in immune composition within the tumour microenvironment may exist between populations.^29^ To investigate these potential variations, we applied the ESTIMATE algorithm to predict the stromal score, immune score, and ESTIMATE score.^30^ Normalized gene expression data from each dataset were processed using the *tidyestimate* package.

Publication bias was evaluated using funnel plots and Egger’s regression test, accounting for within-study dependency. A multilevel meta-regression model was employed, incorporating a variance-covariance structure to adjust for correlated effect sizes within the same study. Egger’s test was performed using robust variance estimation to account for clustering at the study level. The presence of potential publication bias was indicated by asymmetry in the funnel plot or a statistically significant result from Egger’s test (p < 0.05). Sensitivity was further assessed through a leave-one-out analysis to evaluate the influence of individual studies on the overall summary effect.

To assess survival rate differences across clinical factors, we applied Kolmogorov–Smirnov tests for two-group comparisons (e.g., high versus low nodal positivity) and Kruskal–Wallis tests for multi-category factors (e.g., chemotherapy regimens). Fisher’s exact test compared chemotherapy agent distribution between Asian and Western countries. Violin plots, generated via *tidyplots* package, was used to visualize distribution differences. A p-value <0.05 was considered significant.

### Role of the funding source

There was no funding source for this study.

## Results

**Fig. 1:**
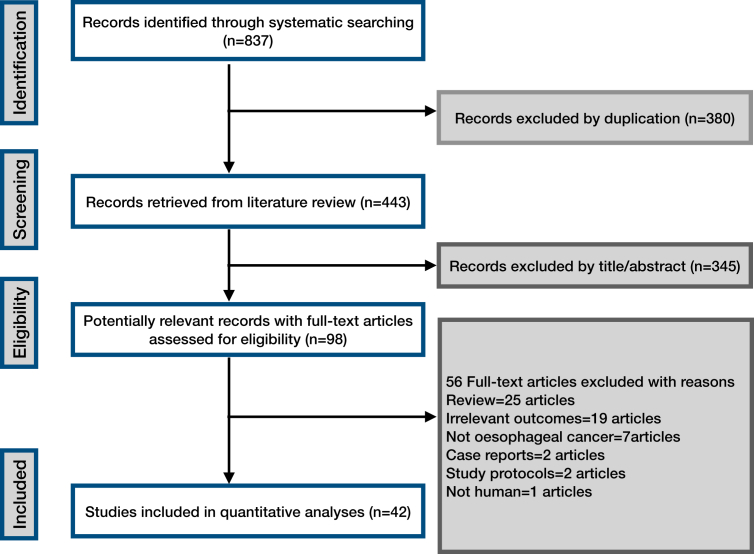
The flowchart of this study.

The results of the literature search are summarised in Fig. 1. The final meta-analysis included 42 studies with 8379 patients who were treated with a range of radiation doses, comprising 13 prospective and 29 retrospective studies. When the location of the trials was considered, the largest group were those conducted in China (33.3%, 14/42), followed by Japan (14.3%, 6/42). All included RCTs had either a low risk of bias or some concerns, with none classified as high risk. Cohort studies were rated as high quality based on the NOS. GRADE assessments showed moderate confidence in OS benefits from prospective studies, though retrospective evidence was less certain. LRPFS was rated highly reliable in prospective trials, while PFS results had lower confidence. Study characteristics and quality/certainty assessments are detailed in Supplementary Tables S4–S7 and Supplementary Fig. S1.

Median splits of study-level factors were shown in Supplementary Table S8. Radiation dose, the primary focus, showed significant differences in 1-year PFS (p = 0.007), 1-year LRPFS (p < 0.001), 2-year LRPFS (p = 0.021), and 3-year LRPFS (p < 0.001) between high-dose and standard-dose (Supplementary Table S8). Chemotherapy regimens were significantly associated with 2-year PFS (p = 0.035), while nodal positivity influenced 5-year LRPFS (p = 0.026). Between-study heterogeneity estimates from each BMA model are summarized in Supplementary Table S9. Among all outcomes, models for OS exhibited the highest heterogeneity, with a mean posterior estimate of τ = 0.6225, compared to 0.5025 for PFS and 0.5125 for LRPFS.

**Fig. 2:**
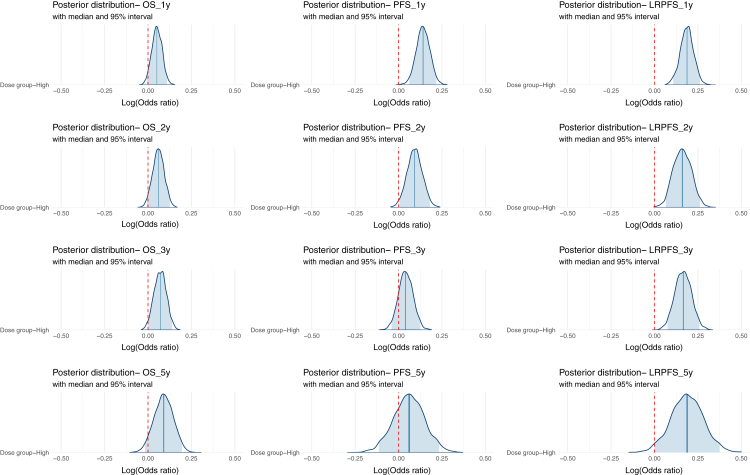
Posterior distributions of effect size (log odds ratio) at 1, 2, 3, and 5 years in patients treated with high-dose versus standard-dose radiation. The median and 95% credible intervals are presented. The red dashed line represents zero survival difference, indicating the point of no effect. OS = overall survival; PFS = progression-free survival; LRPFS = locoregional progression-free survival.

The posterior distributions for 3-year OS favored the high-dose group (median difference of log-odds ratio = 7%, 95% credible interval [CrI]: 0.01%-13.9%, BF = 1.93) (Fig. 2, Supplementary Table S10). For PFS, posterior distributions at 1-year (95% CrI: 6.3%-21.7%, BF = 14.7) and 2-year (95% CrI: 0.5%-17.9%, BF = 2.79) were concentrated on positive values, suggesting a higher likelihood of improved outcomes. This effect diminished at 3- and 5-year PFS (BFs = 1.15 and 1.02, respectively). The strongest effect was seen in LRPFS, where posterior distributions consistently favored the high-dose group. The largest difference occurred at 1-year LRPFS (18.6%, 95% CrI: 10.7%-26.1%, BF = 13.6). A sensitivity analysis stratified by region (China versus Non-China) and study design (Prospective versus Retrospective) revealed some variability in effect estimates. Chinese studies showed a stronger association between dose escalation and OS (Supplementary Table S11). Prospective studies indicated greater short-term survival benefits, notably in 1-year PFS (21.5%, 95% CrI: 10.2%-32.9%, BF = 17.7) and 3-year OS (10.6%, 95% CrI: 1.5%-19.6%, BF = 3.02). Retrospective studies consistently reported higher LRPFS across all time points. Non-Chinese studies exhibited stronger evidence for improved short-term disease control, particularly in 1-year LRPFS (25.3%, 95% CrI: 14.6%-36.0%, BF = 758.9) and 1-year PFS (23.9%, 95% CrI: 7.2%-40.8%, BF = 27.9), suggesting a potential regional variation in treatment effects. Despite the use of estimated values in some studies, the number at risk demonstrated consistent trends across time points.

**Fig. 3:**
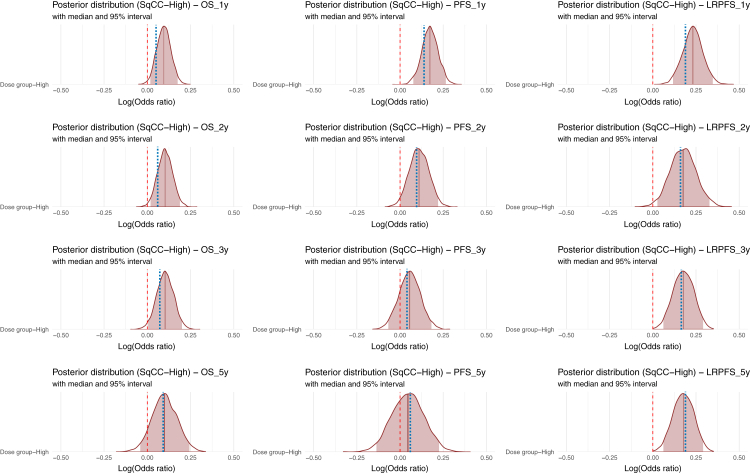
Posterior distributions of effect size (log odds ratio) at 1, 2, 3, and 5 years in patients with SqCC treated by high-dose versus standard-dose radiation. The median and 95% credible intervals are presented. The red dashed line represents zero survival difference, indicating the point of no effect. The blue dashed line indicates survival rate differences without stratification. OS = overall survival; PFS = progression-free survival; LRPFS = locoregional progression-free survival.

Meta-regression analyses identified SqCC and tumour location (middle/lower oesophagus) as significant survival moderators (Supplementary Fig. S2). Subgroup BMA showed a rightward shift in posterior distributions and increased BFs for studies with a high SqCC proportion, indicating a stronger survival benefit with high-dose radiation (Fig. 3, Supplementary Table S12). The largest survival difference was 4.3% for 1- and 2-year OS, with similar patterns observed for PFS (1- and 2-year) and LRPFS (1-, 2-, and 3-year). In contrast, middle/lower oesophageal cancer showed posterior distributions symmetrically centered around zero, suggesting limited dose-related survival benefits, except for a slight positive shift in 2-year LRPFS (Supplementary Fig. S3). Radiation technique subgroup analysis showed no significant survival advantage for high-dose radiation (Supplementary Fig. S4 and Table S13). For SqCC-rich studies (SqCC ≥87.8%), Asian studies reported a higher survival benefit (average BF = 48.9) compared to Western studies (BF = 2.55) (Supplementary Table S14). Despite an imbalance in study numbers (31 Asian versus 3 Western), Western studies had a significantly higher proportion of patients with ECOG PS ≥1 (Supplementary Fig. S5). This trend persisted even when the threshold for identifying SqCC-rich studies was lowered to 50% (7 Western studies), though the ECOG difference was no longer statistically significant (Supplementary Table S15).

**Fig. 4:**
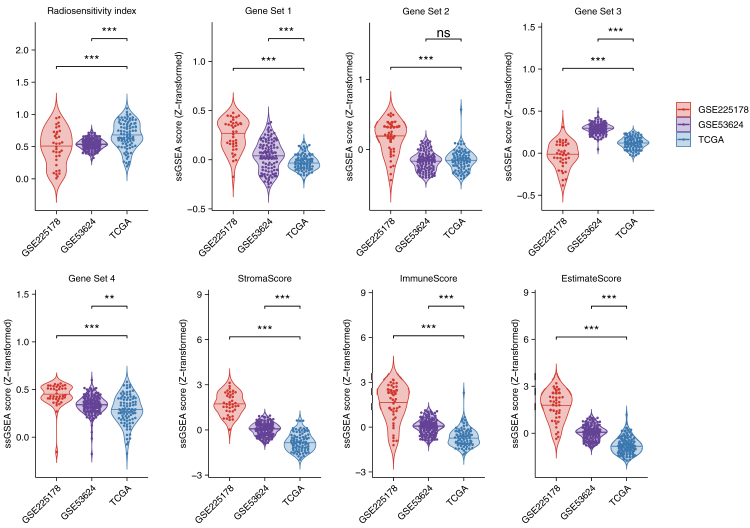
Violin plots depicting the ssGSEA scores (Z-transformed) across three datasets: GSE225178 (red), GSE53624 (purple), and TCGA (blue). The analyzed features include radiosensitivity index, gene set enrichment scores (Gene Sets 1–4), StromaScore, ImmuneScore, and EstimateScore. Statistical significance was assessed between groups, with ∗∗∗p < 0.001, ∗∗p < 0.01, and ∗p < 0.05. “ns” indicates no significant difference. ssGSEA = single-sample Gene Set Enrichment Analysis.

RSI was significantly higher in TCGA samples, suggesting greater radioresistance than in Chinese and Japanese cohorts (Fig. 4). Asian patients showed higher ssGSEA scores for CRTx-responsive gene sets (Gene Sets 1 & 4), while chemoradioresistance-related gene sets (Gene Sets 2 & 3) exhibited conflicting results. Additionally, stromal and immune cell infiltration was consistently higher in Asian patients, indicating a stronger tumour microenvironment influence in SqCC.

PMA found no significant differences in failure or response rates between radiation dose groups (Supplementary Table S16). However, in the high-dose group, IMRT/VMAT was associated with a higher CR rate (0.762; 95% CI: 0.563–0.888; p = 0.039, Table 1). The high-dose group had a higher incidence of any-grade pneumonitis than the standard-dose group (22.7% versus 7.99%; p = 0.011, Table 2), but ≥grade 3 pneumonitis did not differ significantly (p = 0.092, Supplementary Table S17). IMRT/VMAT showed a trend toward lower any-grade toxicity, though not statistically significant (Supplementary Table S18). IMRT/VMAT also significantly reduced neutropenia (53.5% versus 92.8%; p < 0.001), but this result was based on a single study, limiting its reliability.

**Table 1:** Subgroup meta-analysis of radiation techniques regarding response rates and failure rates in high-dose.

	Subgroup	No. of studies	No. of patients	No. of events	Estimated proportion	95% CI	I2 (%)	p (test of subgroup difference)
PR + CR	IMRT/VMAT	10	351	303	0.867	0.812; 0.907	39.2	0.822
	2D/3D CRT		322	277	0.856	0.745; 0.923	74.7	
CR	IMRT/VMAT	7	226	172	0.762	0.563; 0.888	89.9	0.039
	2D/3D CRT		214	93	0.433	0.218; 0.679	90.6	
Any failure	IMRT/VMAT	8	242	150	0.623	0.378; 0.818	90.1	0.752
	2D/3D CRT		302	172	0.568	0.325; 0.782	93.9	
LR failure	IMRT/VMAT	9	356	102	0.284	0.224; 0.352	47.2	0.938
	2D/3D CRT		390	113	0.290	0.156; 0.475	89.3	
Distant failure	IMRT/VMAT	9	356	75	0.212	0.163; 0.270	38.4	0.499
	2D/3D CRT		390	70	0.182	0.124; 0.258	73.9	

PR = partial response; CR = complete response; LR = locoregional; RP = radiation pneumonitis; CI = confidence interval; IMRT = intensity-modulated radiotherapy; VMAT = volumetric-modulated therapy; CRT = conformal radiotherapy.

**Table 2:** Difference of any-grade toxicities between high-dose and standard-dose.

	Subgroup	No. of studies	No. of patients	No. of events	Estimated proportion	95% CI	I2 (%)	p (test of subgroup difference)
Anemia	High-dose	10	362	220	0.645	0.244; 0.911	91.7	0.583
	Standard-dose		703	327	0.504	0.087; 0.915	93.2	
Leukopenia	High-dose	8	362	304	0.839	0.722; 0.913	74.0	0.534
	Standard-dose		345	242	0.757	0.418; 0.931	88.9	
Neutropenia	High-dose	9	393	305	0.751	0.292; 0.957	92.3	0.378
	Standard-dose		564	245	0.477	0.137; 0.841	92.9	
Thrombocytopenia	High-dose	8	406	101	0.264	0.150; 0.419	87.6	0.941
	Standard-dose		484	108	0.249	0.049; 0.681	94.1	
Fatigue	High-dose	2	126	57	0.452	0.368; 0.539	0	0.186
	Standard-dose		73	26	0.356	0.255; 0.472	NA	
Nausea	High-dose	6	288	103	0.356	0.292; 0.425	65.7	0.606
	Standard-dose		478	101	0.246	0.043; 0.704	92.8	
Dysphagia	High-dose	7	268	79	0.318	0.144; 0.563	89.5	0.206
	Standard-dose		660	321	0.489	0.374; 0.606	84.6	
Esophagitis	High-dose	11	466	301	0.766	0.295; 0.962	92.2	0.496
	Standard-dose		494	222	0.577	0.243; 0.853	93.8	
Heart	High-dose	3	120	3	0.035	0.013; 0.097	0	0.375
	Standard-dose		471	9	0.020	0.011; 0.039	0	
Fistula	High-dose	4	272	12	0.047	0.013; 0.149	66.6	0.823
	Standard-dose		316	17	0.055	0.024; 0.121	63.9	
Stricture	High-dose	2	142	9	0.067	0.035; 0.125	2.7	0.412
	Standard-dose		117	5	0.043	0.018; 0.099	0	
Pneumonitis	High-dose	7	455	93	0.227	0.145; 0.339	75.9	0.011
	Standard-dose		233	23	0.079	0.039; 0.156	58.3	

CI = confidence interval.

To assess the impact of pneumonitis on OS, we analyzed any-grade and grade 3 or higher pneumonitis. Meta-regression, adjusted for confounders, found no significant association for either any-grade or grade 3 or higher pneumonitis (Supplementary Table S19).

**Fig. 5:**
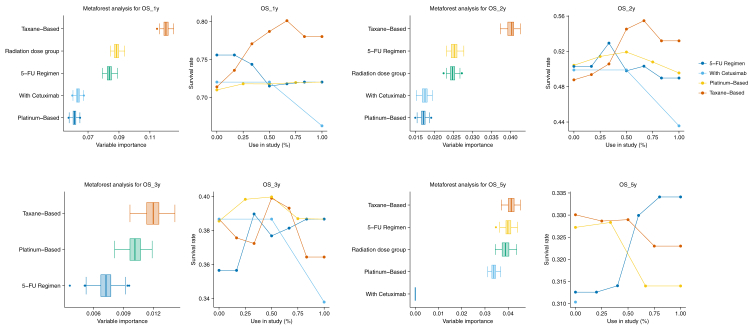
MetaForest analysis of overall survival at 1, 2, 3, and 5 years. (Left) Variable importance plots depicting the relative contribution of treatment-related factors. (Right) Partial dependence plots depicting the association between the percentage of chemotherapy regimen usage and survival rates. OS = overall survival.

Platinum was the most used agent (n = 40 studies), followed by 5-FU (n = 28 studies) and taxane (n = 15 studies), with Cetuximab in only four studies. No significant differences in chemotherapy usage were observed between Asia and Western countries (Supplementary Table S20). The patient characteristics were generally not associated with improved predictive performance in the MetaForest models (Supplementary Table S21). The inclusion of these variables in the full model yielded minimal or no gain in OOB-R^2^, and none reached statistical significance by paired t-test. As a result, subsequent analyses were conducted using the treatment-only models. MetaForest analysis identified taxane-based regimens as the strongest predictor of OS at 1- and 2-years, but their impact declined at 3- and 5-years (Fig. 5). Partial dependence plots confirmed increased survival with taxane use at early time points, while its long-term benefit remained uncertain. Conversely, 5-FU was associated with a positive effect on 5-year OS. For PFS, taxane was predictive only at 1- and 2-years, mirroring its OS trend, whereas 5-FU had a more positive impact at 5-year (Supplementary Figs. S6 and S7). However, for LRPFS, radiation dose was the dominant factor at 1-, 2-, and 3-years, while 5-FU emerged as the strongest predictor at 5-years (Supplementary Figs. S8 and S9). Radiation dose groups contributed to survival differences but were less predictive of OS than chemotherapy (Supplementary Fig. S10). In Asian populations, taxane was more strongly associated with OS at 1-, 2-, and 5-years, but its PFS benefit was limited to 1-year (Supplementary Figs. S11–S14). Due to insufficient data for model convergence, Western populations were not analyzed.

Funnel plots are presented in Supplementary Fig. S15. No apparent publication bias was observed across studies, except for 1-year OS, 2-/3-year PFS, total response rate, neutropenia, thrombocytopenia, nausea, and fistula. To assess the robustness of the findings, leave-one-out sensitivity analyses were performed and summarized in Supplementary Fig. S16. In all cases, the 95% CIs of the re-estimated effects overlapped with the overall main estimate, indicating that no single study had a disproportionate influence on the pooled results and suggesting the absence of influential outliers.

## Discussion

This meta-analysis of 8379 patients from 42 studies supports radiation dose escalation (BED ≥70 Gy) as a survival benefit in locally advanced oesophageal cancer, particularly for Asian populations. The benefit appeared pathology-dependent, with SqCC showing improved OS, PFS, and LRPFS, likely due to distinct tumour biology in Asian patients, including higher stromal/immune infiltration and CRTx-sensitive genomic profiles. While high-dose increased any-grade pneumonitis risk, it had no significant impact on OS. These findings highlight the influence of demographic factors on high-dose outcomes, emphasizing the need for region-specific treatment strategies.

Radiation dose escalation in CRTx for locally advanced oesophageal cancer remains controversial, with RCTs and real-world studies yielding mixed results. The ARTDECO trial found no LRPFS, OS, or PFS benefit from 61.6 Gy (BED = 75.15) over 50.4 Gy, though a 10% lower chemotherapy (Paclitaxel + Carboplatin) compliance in the high-dose arm may have influenced outcomes.^9^ Similarly, another phase III trial reported a smaller chemotherapy adherence gap (67.6% versus 72.6%) but still found no OS benefit in thoracic SqCC treated with Paclitaxel + Carboplatin.^31^ In contrast, our meta-analysis of 42 studies, accounting for clinical variables and systemic therapy regimens, showed high-dose improved OS, particularly in SqCC, aligning with recent meta-analyses.^32^^,^^33^ The discordant outcomes of dose-escalation trials likely stem from biological and demographic heterogeneity. Western cohorts, predominantly composed of adenocarcinoma, exhibit greater chemo-sensitivity, as evidenced by the superiority of FLOT over neoadjuvant CRTx for adenocarcinoma.^34^ Additionally, studies with middle/lower oesophagus had a lower SqCC proportion (79.2% versus 97.5%), mirroring the lack of OS benefit in this subgroup. These findings emphasize that while high-dose consistently improves LRPFS, OS benefits at 1- and 2-year are largely SqCC-specific, underscoring the need for histology-driven dose-escalation strategies. Additionally, trial design limitations may contribute to the divergent findings. ARTDECO and You et al. were limited by modest sample sizes, narrow patient eligibility, and insufficient power to detect subgroup-specific benefits. Moreover, toxicity-related modifications, and salvage interventions may have diluted potential benefits of high-dose in these trials.

High dose appears to confer greater survival benefits in Asian patients with SqCC compared to their Western counterparts, a disparity that may be driven by molecular differences and treatment practices. Genomic studies revealed higher frequencies of *TP53* and *NFE2L2* mutations in tumours from Chinese patients with SqCC, which could influence radiation response.^35^^,^^36^ However, these mutations are typically associated with radioresistance rather than enhanced radiosensitivity, suggesting they may not fully account for the improved outcomes observed in Asian cohorts.^37^^,^^38^ Therefore, other genomic and microenvironmental factors likely contribute to the enhanced treatment efficacy in this population. Notably, the gene signatures analyzed in this study were all linked to tumour immunity. Specifically, *SLC27A5* and *ALOXE3*, part of gene set 1, were positively correlated with *CD274* (*PD-L1*) and *CD40* expression, both of which play key roles in immune modulation.^28^ Gene set 2, comprising *CD180* and *CIMP*, includes molecules involved in immune signaling pathways, particularly Toll-like receptor signaling and inflammatory responses.^27^ Furthermore, gene set 3 represents the epithelial-mesenchymal transition score, which has been inversely correlated with immune activation.^26^ Lastly, gene set 4 directly encompasses genes involved in immune response regulation.^25^ These studies, from which the gene sets were derived, further support the association between increased immune activation, enhanced anti-tumour immune responses, and improved outcomes following CRTx in SqCC. These findings emphasize the tumour immune microenvironment's crucial role in treatment response and its potential as a determinant of therapeutic efficacy.^39^ Higher radiosensitivity, immune infiltration, and activation of CRTx-favorable pathways in Asian cohorts suggest a more responsive SqCC phenotype to high-dose. Clinical evidence suggests camrelizumab, alone or with RT (50–63 Gy), is effective and well-tolerated in Asian SqCC patients. Those receiving >4 cycles, especially with a favorable Lung Immune Prognostic Index, had prolonged OS and PFS, underscoring the role of immune response.^40^ In contrast, high-dose CRTx appears less effective in Western patients, highlighting the need for further research to identify responsive subpopulations and optimize treatment strategies.^41^

Our MetaForest analysis highlights the dominant role of chemotherapy in survival outcomes, with taxane-based regimens showing the strongest association with OS and PFS at 1-/2-year, though their impact waned over time. 5-FU-based regimens had a more limited but positive effect on long-term OS. These findings suggest that taxane-based therapy may reduce the benefit of high-dose, particularly in Asian populations, where taxane regimens had a greater impact on OS. The observed attenuation of taxane-associated survival gains at 3 and 5 years may reflect the limitations of taxanes in sustaining long-term tumour control. Mechanistically, taxanes exert potent cytotoxic effects by disrupting mitosis, resulting in rapid tumour regression. However, compared to 5-FU, taxanes more profoundly affect tumour vasculature and stromal integrity.^42^ When combined with high-dose radiation, this disruption may exacerbate ischemia and hypoxia, impair drug delivery, and create a microenvironment conducive to cancer stem cell survival. Furthermore, cumulative toxicities associated with taxanes can compromise treatment adherence and limit the administration of subsequent therapies. Together, these factors may contribute to the diminished long-term survival benefit observed in recent cohorts with increased taxane use.

Among Asian patients, 3-year OS ranged from 22.4%–51.8% with Cisplatin/5-FU, improving to 55.4% with Paclitaxel/5-FU and 53.1% with Docetaxel/Cisplatin.^8^^,^^43^, ^44^, ^45^ Notably, Paclitaxel/5-FU was associated with higher pneumonitis risk (8.8% versus 2.7% for Cisplatin/5-FU), though severe cases were similar. A meta-analysis further confirmed better clinical responses, OS, and PFS for taxane-based treatment in SqCC compared to 5-FU-based regimens.^46^ Additionally, Cetuximab-containing regimens showed no survival advantage, consistent with the SCOPE1 trial.^47^ Given taxane’s strong survival impact, failure patterns should be further investigated. Additional studies are needed to evaluate its role in combination with high-dose and determine whether it negates the potential benefits of dose escalation.

Despite the potential benefits of high-dose, toxicity remains a concern. Our analysis found higher any-grade pneumonitis in the high-dose group, though ≥grade 3 pneumonitis was not significantly different. The low incidence of severe pneumonitis (3.5%) may explain its lack of association with OS.^48^ Modern techniques in high-dose showed a trend toward reduced toxicity, aligning with findings from Freilich et al., where IMRT lowered ≥grade 3 toxicities without compromising survival compared to 3D-CRT (median dose: 50.4 Gy).^49^ Similarly, Suh et al. reported proton therapy reduced lung/heart exposure versus photon therapy, with no survival difference.^50^ Our analysis also linked modern techniques to higher CR rates in high-dose, though their survival impact remains unclear. No prospective studies have compared IMRT versus 3D-CRT at escalated doses, highlighting the need for further validation. These findings suggest that IMRT and proton therapy may enhance the therapeutic ratio by reducing toxicity while maintaining efficacy.

Our analysis included a substantial proportion of studies from China, where treatment practices—such as the use of higher radiation doses (in our included studies, median BED = 72 Gy in China versus 63 Gy in non-China regions)—may differ from Western guidelines. While subgroup analyses revealed consistent directionality of dose-escalation effects across regions, differences in healthcare systems, centralized surgical care, and variable access to advanced technologies could influence treatment outcomes. These contextual factors should be carefully considered when generalizing our findings to non-China regions.

By integrating PMA with Bayesian methods, our analysis addresses key limitations of prior dose-escalation trials by accounting for model uncertainty, incorporating prior knowledge, and managing heterogeneity.^16^ BMA improves estimation by borrowing strength across subgroups, providing stable survival estimates while capturing dose-response interactions with systemic therapies. Unlike frequentist models, the Bayesian approach offers direct probabilistic interpretations, aiding clinical decision-making when survival differences are small but meaningful. BFs further quantify the strength of evidence for high-dose benefits, providing a robust framework to integrate heterogeneous data while adjusting for chemotherapy regimens and patient characteristics.

This study has several limitations. First, retrospective data aggregation introduces biases, including variations in staging, chemotherapy, radiation techniques, and follow-up. Definitions of PFS and LRPFS were not standardized across all studies, and inconsistencies in reporting may introduce outcome misclassification. Although we made efforts to harmonize definitions—for example, treating LPFS as a surrogate for LRPFS in studies lacking regional data—this may result in underestimation of true locoregional event rates. Data on additional interventions such as salvage surgery or curative treatment of recurrence were inconsistently reported across the included studies. Indications, and outcome reporting for these procedures varied substantially or were omitted altogether, limiting the feasibility of meaningful subgroup analyses. This lack of standardized reporting may obscure their potential contribution to long-term survival and represents an important area for improvement in future research. Second, while BMA addressed heterogeneity, residual confounding from unmeasured factors (e.g., staging methods, molecular subtypes, treatment compliance, and toxicity management) remains. Third, BMA in our study relies on PMA results, which may be biased in small samples. Fourth, the number of patients at risk for survival analysis was estimated for studies lacking risk tables by multiplying the total cohort size by reported survival rates at each time point. This approach assumes uniform censoring and may introduce bias, especially in studies with differential follow-up or incomplete reporting, potentially affecting the precision of survival estimates. Future investigations leveraging individual patient data would enable more accurate time-to-event analyses and mitigate these limitations. Fifth, high-dose may increase toxicity, impacting treatment adherence, but limited patient-level data restricted a more granular analysis. Although pneumonitis was not linked to OS, underreporting of toxicity-related treatment modifications may have influenced survival. Lastly, genomic and immune profiling revealed differences between Asian and Western SqCC, but limited transcriptomic data hindered further stratification. Future studies using single-cell sequencing and spatial transcriptomics could provide deeper insights into tumour-immune interactions and treatment response. While the RSI analysis provides valuable biological insight, it is important to note that the publicly available datasets used may not fully represent the clinical populations included in our meta-analysis. Differences in patient demographics, treatment protocols, and geographic distribution—particularly the underrepresentation of Asian cohorts—limit the direct generalizability of these findings. Therefore, the RSI results should be interpreted as exploratory and hypothesis-generating rather than directly predictive for clinical decision-making.

Our meta-analysis highlights that radiation dose escalation (BED ≥70 Gy) improves LRPFS and may enhance OS in locally advanced oesophageal cancer, particularly for SqCC. Outcomes vary by histology, region, and chemotherapy, with taxane-based regimens being key OS predictors. Genomic and immune differences between Asian and Western SqCC may affect response. Modern radiation techniques show potential, but prospective studies are needed to optimize personalized, high-dose treatment strategies.

## Contributors

Conceptualization: Yang-Hong Dai, Yi-Jhih Huang; Data collection: Yang-Hong Dai, Po-Huang Chen, Ding-Jie Lee, Accessed and verified the data: Po-Chien Shen, Cheng-Hsiang Lo, Yu-Guang Chen, Yu-Fu Su, Jen-Fu Yang, Ying-Fu Wang, Wen-Yen Huang, Chun-Shu Lin; Formal analysis: Yang-Hong Dai, Po-Huang Chen; Writing—original draft: Yang-Hong Dai; Writing—review & editing: Chih-Cheng Tsao, Katherine A. Vallis; Project administration: Chih-Cheng Tsao, Katherine A. Vallis. All authors read and approved the final draft of the manuscript.

## Data sharing statement

The data that support the findings of this study are available from the corresponding author upon reasonable request.

## Declaration of interests

The authors have stated that they have no conflict of interest.
