# Supplementary material for: Radiation dose escalation in locally advanced oesophageal cancer: a systematic review and hierarchical Bayesian meta-analysis
Source: eClinicalMedicine. 2025 Aug 13;87:103432. doi: 10.1016/j.eclinm.2025.103432 (PMC12362008; doi:10.1016/j.eclinm.2025.103432)
Supplement: Supplementary Figs. S1–S16 and Tables S1–S21 [file mmc1.docx]

**Supplementary material**

**Supplementary Table S1. Searching terms used in this study.**

| ("Esophageal Neoplasms"[Title/Abstract] OR esophageal[Title/Abstract] OR oesophageal[Title/Abstract] OR esophagus[Title/Abstract]) AND ("Neoplasms"[Title/Abstract] OR tumor[Title/Abstract] OR cancer[Title/Abstract] OR carcinoma[Title/Abstract]) AND ("Chemoradiotherapy"[Title/Abstract] OR chemoradiation[Title/Abstract] OR chemoradiotherapy[Title/Abstract] OR radiochemotherapy[Title/Abstract] OR chemo-irradiation[Title/Abstract] OR chemo-radiotherapy[Title/Abstract]) AND ("Radiotherapy Dosage"[Title/Abstract] OR dose[Title/Abstract] OR "dose escalation"[Title/Abstract] OR "radiation dose"[Title/Abstract]) AND (("Clinical Trial"[Publication Type] OR "Clinical Trials as Topic"[MeSH Terms] OR "clinical trial"[All Fields]) OR ("Retrospective Studies"[MeSH Terms] OR "retrospective study"[All Fields])) |
| --- |

| **Supplementary Table S2. Calculation of radiosensitivity index (RSI).** | |
| --- | --- |
| **Ten genes for RSI** | AR, JUN, STAT1, PRKCB, RELA, ABL1, SUMO1, CDK, HDAC1, IRF1 |
| **Equation for calculating RSI** | –AR*0.0098009 + JUN*0.0128283 + STAT1*0.0254552 – PRKCB*0.0017589–RELA*0.0038171+ ABL1*0.1070213–SUMO1*0.0002509 – CDK1*0.0092431–HDAC1*0.0204469–IRF1*0.0441683 |

| **Supplementary Table S3. Gene sets for predicting treatment response of chemoradiotherapy in esophageal squamous cell carcinoma.** | | |
| --- | --- | --- |
| **Gene set 1** | *ATF2*, *SLC27A5*, *ALOXE3* | 1. **ATF2 (Activating Transcription Factor 2)**    - A transcription factor involved in stress response, apoptosis, and tumor progression.    - Plays a role in **DNA damage response**, **P53 signaling**, and **epithelial-mesenchymal transition (EMT)**.    - Can influence tumor resistance to therapy by regulating gene expression under stress conditions. 2. **SLC27A5 (Solute Carrier Family 27 Member 5, also known as FATP5)**    - Involved in **fatty acid transport and metabolism**, particularly in lipid uptake and β-oxidation.    - Dysregulation may impact tumor cell energy metabolism and therapy sensitivity.    - Potentially linked to immune cell infiltration in the tumor microenvironment. 3. **ALOXE3 (Arachidonate Lipoxygenase 3)**    - A lipid-metabolizing enzyme involved in **oxidative lipid signaling** and **skin differentiation pathways**.    - Can regulate **tumor-associated inflammation and immune responses**.    - May influence tumor progression and response to therapy by affecting lipid peroxidation and oxidative stress.   These genes collectively contribute to **chemoradiotherapy sensitivity in SqCC** by regulating key pathways such as **P53 signaling, EMT, immune interactions, and lipid metabolism**. |
| **Gene set 2** | *CTSS*, *CD180*, *SCIMP* | 1. **CTSS (Cathepsin S)**    - A cysteine protease involved in **protein degradation**, **antigen processing**, and **tumor invasion**.    - Plays a role in **tumor immune evasion**, promoting cancer progression by degrading extracellular matrix components.    - Associated with **radioresistance** through its role in **tumor microenvironment remodeling** and **inflammatory response**. 2. **CD180 (Cluster of Differentiation 180, also known as RP105)**    - A Toll-like receptor (TLR)-like molecule involved in **immune regulation and B-cell activation**.    - Contributes to **radioresistance** by modulating **inflammatory signaling and immune escape**.    - May promote **cancer cell survival** under radiation-induced stress through interactions with the **tumor immune microenvironment**. 3. **SCIMP (SLP Adapter and CSK-Interacting Membrane Protein)**    - A transmembrane adaptor protein associated with **T-cell receptor signaling and immune modulation**.    - Regulates **antigen presentation** and immune response in tumor cells.    - Its involvement in **radioresistance** may be linked to immune evasion and alterations in **tumor-associated signaling pathways**.   These three genes collectively contribute to **radioresistance in SqCC** by influencing **tumor-immune interactions, extracellular matrix remodeling, and inflammatory signaling**, providing potential targets for prognosis and therapeutic intervention. |
| **Gene set 3** | *CLEC18A*, *PIR*, *KCNN4*, *MST1R*, *CAPG*, *ALDH5A1*, *COX7B* | 1. **CLEC18A (C-Type Lectin Domain Family 18 Member A)**    - Involved in **immune response and cell adhesion**.    - May play a role in **tumor progression** by influencing **cell-matrix interactions** and immune regulation.    - Potentially contributes to **EMT and chemoradiotherapy resistance**. 2. **PIR (Pirin)**    - A nuclear protein involved in **oxidative stress response and transcriptional regulation**.    - Enhances **EMT and tumor invasion**, contributing to **radioresistance and poor prognosis**.    - Plays a role in **NF-κB signaling**, which is linked to **inflammation and therapy resistance**. 3. **KCNN4 (Potassium Calcium-Activated Channel Subfamily N Member 4)**    - A potassium ion channel involved in **cell migration, proliferation, and survival**.    - Regulates **calcium signaling**, influencing **tumor progression and therapy resistance**.    - Associated with **increased metastasis and EMT activation** in several cancers. 4. **MST1R (Macrophage-Stimulating 1 Receptor, also known as RON)**    - A receptor tyrosine kinase involved in **tumor cell survival, motility, and EMT**.    - Activation of MST1R enhances **radiation resistance** by promoting **pro-survival signaling pathways**.    - Plays a role in **immune modulation and tumor microenvironment interactions**. 5. **CAPG (Macrophage-Capping Protein)**    - Regulates **actin cytoskeleton remodeling**, impacting **cell motility and invasion**.    - Linked to **cancer metastasis** and **EMT-driven resistance to chemoradiotherapy**.    - May contribute to **tumor immune evasion and therapy adaptation**. 6. **ALDH5A1 (Aldehyde Dehydrogenase 5 Family Member A1)**    - Involved in **cellular detoxification and oxidative stress response**.    - Plays a role in **cancer cell survival under hypoxia and stress conditions**.    - May modulate **therapy resistance through metabolic adaptation**. 7. **COX7B (Cytochrome C Oxidase Subunit 7B)**    - A mitochondrial electron transport chain component critical for **cellular respiration and energy metabolism**.    - May be involved in **oxidative phosphorylation alterations** that support **tumor survival and therapy resistance**.    - Potentially linked to **radioresistance through metabolic reprogramming**.   These **seven genes collectively influence EMT, tumor metabolism, oxidative stress, and immune modulation**, contributing to **neoadjuvant chemoradiotherapy resistance in SqCC** and serving as potential biomarkers for prognosis and therapeutic targeting. |
| **Gene set 4** | *SERPINE1*, *MMP12*, *PLAUR*, *EPS8* | 1. **SERPINE1 (Serpin Family E Member 1, also known as PAI-1)**    - A serine protease inhibitor involved in **fibrinolysis, tumor progression, and immune modulation**.    - Promotes **EMT**, contributing to **tumor invasion and therapy resistance**.    - Regulates the **tumor microenvironment (TME)** by influencing **angiogenesis and immune suppression**. 2. **MMP12 (Matrix Metalloproteinase 12, also known as Macrophage Metalloelastase)**    - A matrix metalloproteinase that degrades **extracellular matrix (ECM) components**, facilitating **tumor invasion and metastasis**.    - Modulates **immune responses** by influencing **macrophage infiltration and inflammatory cytokine signaling**.    - Implicated in **tumor resistance to therapy** by remodeling the ECM and affecting immune cell recruitment. 3. **PLAUR (Plasminogen Activator, Urokinase Receptor, also known as uPAR)**    - A receptor involved in **cell adhesion, migration, and immune regulation**.    - Enhances **tumor cell survival and metastasis** by activating **proteolytic cascades**.    - Contributes to **immune evasion** by modulating **inflammatory responses and TME interactions**. 4. **EPS8 (Epidermal Growth Factor Receptor Pathway Substrate 8)**    - A signaling adaptor protein that regulates **EGFR-mediated cell proliferation, migration, and cytoskeletal reorganization**.    - Involved in **tumor progression and resistance to therapy** through **EMT activation**.    - Affects **immune response** by interacting with signaling pathways that control **tumor-immune interactions**.   These **four immune-related genes collectively influence tumor invasion, EMT, immune modulation, and extracellular matrix remodeling**, contributing to **chemoradiotherapy response and prognosis prediction in SqCC**. Their integration into a predictive immune signature provides a **clinically applicable biomarker for individualized treatment strategies**. |

| **Supplementary Table S4. Study characteristics.** | | |
| --- | --- | --- |
|  | **Number** | **Percentage (%)** |
| **Countries** |  |  |
| China | 14 | 33.3 |
| Japan | 6 | 14.3 |
| Taiwan | 4 | 9.5 |
| USA | 4 | 9.5 |
| Korea | 3 | 7.1 |
| Netherlands | 3 | 7.1 |
| Germany | 2 | 4.8 |
| France | 2 | 4.8 |
| UK | 1 | 2.4 |
| India | 1 | 2.4 |
| Norway | 1 | 2.4 |
| Sweden | 1 | 2.4 |
| **Study type** |  |  |
| Prospective | 13 | 31 |
| Retrospective | 29 | 69 |
| **Gender** |  |  |
| Male | 6888 | 83.5 |
| Female | 1361 | 16.5 |
| **Performance status** |  |  |
| ECOG=0 | 2619 | 58.0 |
| ECOG≥1 | 1913 | 42.0 |
| **Pathology** |  |  |
| Adenocarcinoma | 1110 | 14.0 |
| Squamous cell carcinoma | 6788 | 85.9 |
| Adenosquamous cell carcinoma | 3 | 0.01 |
| **Size** |  |  |
| < 5 cm | 881 | 46.9 |
| > 5 cm | 998 | 53.1 |
| Length |  |  |
| **T stage** |  |  |
| T12 | 1026 | 26.4 |
| T34 | 2863 | 73.6 |
| **N stage** |  |  |
| N0 | 1300 | 31.4 |
| N+ | 2834 | 68.6 |
| **M stage** |  |  |
| M0 | 4961 | 95.1 |
| M+ | 256 | 4.9 |
| **Clinical stage** |  |  |
| Stage 1 or 2 | 1288 | 24.7 |
| Stage 3 or 4 | 3929 | 75.3 |
| **Localization** |  |  |
| Cervical/upper esophagus | 1822 | 34.5 |
| Middle/lower esophagus | 3454 | 65.5 |
| ECOG= Eastern Cooperative Oncology Group. | | |

| **Supplementary Table S5. Detailed study characteristics.** | | | | | | | | |
| --- | --- | --- | --- | --- | --- | --- | --- | --- |
| **First author** | **Year** | **Country** | **Study type** | **BED** | **RT technique** | **Dose escalation** | **Chemotherapy** | **Outcomes** |
| Arnold Herskovic_CRT | 1992 | USA | P | 60 | 3DCRT |  | Cisplatin + 5-FU | OS, any failure, LR failure, distant failure |
| Arnold Herskovic_RT | 1992 | USA | P | 76.8 | 3DCRT | - Initial phase: regional field: 50 Gy. - Escalation phase: boost of 14 Gy to primary tumor. - No tissue inhomogeneity correction. | Cisplatin + 5-FU | OS, any failure, LR failure, distant failure |
| Bruce D Minsky_SD | 2002 | USA | P | 59.5 | 3DCRT |  | Cisplatin + 5-FU | OS, any failure, LR failure, distant failure |
| Bruce D Minsky_HD | 2002 | USA | P | 76.5 | 3DCRT | - Initial phase: regional field: 50.4 Gy. - Escalation phase: boost of 14.4 Gy to primary tumor. | Cisplatin + 5-FU | OS, any failure, LR failure, distant failure |
| Kaoru Ishida | 2004 | Japan | P | 72 | 3DCRT | - T-shape filed (bilateral supraclavicular nodes + mediastinum): 60 Gy - For regional field at the celiac region, dose was reduced to 46 Gy | Cisplatin + 5-FU | OS, response rate (PR+CR) |
| Ryo Takagawa | 2009 | Japan | R | 72 | 2D | - Initial phase: up to 40 Gy using anterior-posterior opposed fields - Escalation phase: boost of 20 Gy to the primary tumor and the locally enlarged lymph nodes using an oblique-opposed technique to exclude the spinal cord from the field. | Cisplatin + 5-FU | OS, response rate (PR+CR, CR) |
| M. Hurmuzlu | 2010 | Norway | R | 79.2 | 3DCRT | - Four fields: AP+oblique fields. - PTV: 50 Gy. - Reduced PTV (different angling of the oblique fields): additional 16 Gy. | Cisplatin + 5-FU | OS, response rate (PR+CR), any failure |
| Jihua Han_CRT | 2012 | China | R | 79.2 | 3DCRT | - Four to six conformal fields. - PTV to 64-66 Gy. | Nedaplatin + 5-FU | OS, PFS, LR failure, distant failure |
| Jihua Han_RT | 2012 | China | R | 79.2 | 3DCRT | - Four to six conformal fields - PTV to 64-66 Gy | Nil | OS, PFS, LR failure, distant failure |
| Wei-Guo Zhu_SD | 2012 | China | R | 72.5 | IMRT | - The dose escalation technique employed the SIB within a simplified IMRT framework. - PGTVnd (gross nodal disease): received a boosted dose in the hsIMRT (high dose) group (68.1 Gy), vs. 60 Gy in csIMRT (conventional dose). - PTV1: received 63.9 Gy in hsIMRT vs. 60 Gy in csIMRT. - PTV2 (elective/prophylactic nodes): both groups received 54 Gy. | Cisplatin + 5-FU | OS, PFS, response rate (PR+CR, CR) |
| Wei-Guo Zhu_HD | 2012 | China | R | 83.6 | IMRT | - The dose escalation technique employed the SIB within a simplified IMRT framework. - PGTVnd (gross nodal disease): received a boosted dose in the hsIMRT (high dose) group (68.1 Gy), vs. 60 Gy in csIMRT (conventional dose). - PTV1: received 63.9 Gy in hsIMRT vs. 60 Gy in csIMRT. - PTV2 (elective/prophylactic nodes): both groups received 54 Gy. | Cisplatin + 5-FU | OS, PFS, response rate (PR+CR, CR) |
| Yasumasa Nishimura_short | 2012 | Japan | R | 72 | 3DCRT | - CTV1: 40 Gy - CTV2: boost of 20 Gy to a reduced volume encompassing the GTV with margins | Cisplatin + 5-FU | OS |
| Yasumasa Nishimura_protracted | 2012 | Japan | R | 72 | 3DCRT | - CTV1: 40 Gy - CTV2: boost of 20 Gy to a reduced volume encompassing the GTV with margins | Cisplatin + 5-FU (protracted) | OS |
| Ken Kato | 2013 | Japan | P | 59.5 | 3DCRT |  | Cisplatin + 5-FU | OS, PFS, response rate (CR), any failure |
| Thomas Crosby | 2013 | UK | P | 60 | 3DCRT |  | Cisplatin+capecitabine | OS, any failure, LR failure, distant failure |
| Thomas Crosby_Cetux | 2013 | UK | P | 60 | 3DCRT |  | Cisplatin+capecitabine+cetuximab | OS, any failure, LR failure, distant failure |
| JB Clavier_SD | 2013 | France | R | 60 |  |  | Cisplatin + 5-FU | OS, any failure, LR failure, distant failure |
| JB Clavier_HD | 2013 | France | R | 79.2 |  | - First phase: anterior-posterior beams to 36 Gy - Escalation phase: Transitioned to three-field technique with anterior and oblique posterior beams, boosting to > 50.4 Gy | Cisplatin + 5-FU | OS, any failure, LR failure, distant failure |
| Xue Meng | 2013 | China | P | 70.1 | 3DCRT | - GTV to 59.4 Gy (1.8 Gy/day) - All fields were treated on each day of therapy | Cetuximab+Paclitaxel+Cisplatin | OS, PFS, response rate (PR+CR, CR), any failure, LR failure, distant failure |
| Thierry Conroy_CF | 2014 | France | P | 60 | IMRT |  | Cisplatin+FU | OS, PFS, response rate (PR+CR), LR failure, distant failure |
| Thierry Conroy_FOLFOX | 2014 | France | P | 60 | IMRT |  | FOLFOX | OS, PFS, response rate (PR+CR), LR failure, distant failure |
| J. Freilich_3DCRT | 2014 | USA | R | 59.5 | 3DCRT |  | Mix: Cisplatin + 5-FU | OS, PFS, LR failure, distant failure |
| J. Freilich_IMRT | 2014 | USA | R | 59.5 | IMRT |  | Mix: Cisplatin + 5-FU | OS, PFS, LR failure, distant failure |
| E Versteijne | 2015 | Netherlands | R | 59.5 | 3DCRT |  | Weekly paclitaxel+carboplatin | OS, LRPFS, LR failure, distant failure |
| Yang-Gun Suh_SD | 2014 | Korea | R | 64.8 | 2D/3DCRT |  | 5-FU+cisplatin or 5-FU alone | OS, PFS, LRPFS, response rate (PR+CR, CR), any failure, LR failure, distant failure |
| Yang-Gun Suh_HD | 2014 | Korea | R | 75.6 | 2D/3DCRT | - Initial phase: AP–PA parallel-opposed fields; 30.6-45 Gy - Escalation phase: GTV boosted to75.6 Gy | 5-FU+cisplatin or 5-FU alone | OS, PFS, LRPFS, response rate (PR+CR, CR), any failure, LR failure, distant failure |
| Katsuhiko Higuchi_SD | 2014 | Japan | R | 59.5 | 3DCRT |  | Docetaxel+Cisplatin+F-FU | Response rate (PR+CR, CR) |
| Katsuhiko Higuchi_HD | 2014 | Japan | R | 72.2 | 3DCRT | - Initial phase: GTV to 39.6 Gy with AP-PA opposed fields - Escalation phase: boost of 21.6 Gy with oblique field | Docetaxel+Cisplatin+F-FU | Response rate (PR+CR, CR) |
| Falk Roeder | 2014 | Germany | R | 66.1 | IMRT |  | Cisplatin + 5-FU | OS, PFS, LRPFS |
| Xiaolin Ge_RT | 2015 | China | R | 72 | IMRT | - PTV was 60 Gy to 64 Gy at 2.0 Gy per fraction in 5 fractions per week. | Cisplatin + 5-FU/Docetaxel | OS, PFS, LRPFS, response rate (PR+CR, CR), LR failure, distant failure |
| Xiaolin Ge_CRT | 2015 | China | R | 72 | IMRT | - PTV was 60 Gy to 64 Gy at 2.0 Gy per fract | Cisplatin + 5-FU/Docetaxel | OS, PFS, LRPFS, response rate (PR+CR, CR), LR failure, distant failure |
| Chih-Yi Chen_SD | 2016 | Taiwan | R | 60 | IMRT/3DCRT |  | NR | OS |
| Chih-Yi Chen_HD | 2016 | Taiwan | R | 70 | IMRT/3DCRT | NR | NR | OS |
| Liru He_3DCRT | 2016 | China | R | 59.5 | 3DCRT |  | Mix: cisplatin+/-taxane+/-5-FU | OS |
| Liru He_IMRT | 2016 | China | R | 59.5 | IMRT |  | Mix: cisplatin+/-taxane+/-5-FU | OS |
| Hoa Yang_VMAT | 2017 | China | R | 79.2 | VMAT | - GTV: 60–70 Gy in 2.0–2.2 Gy/fraction (5 fractions/week) - PTV: 54–60 Gy in 1.8–2.0 Gy/fraction - PTV-elective: 45–54 Gy in 1.8–2.0 Gy/fraction - VMAT: Two coplanar full arcs - Total dose: 60–70 Gy | Mix: cisplatin+/-taxane+/-5-FU | OS, PFS, response rate (PR+CR, CR) |
| Hoa Yang_3DCRT | 2017 | China | R | 79.2 | 3DCRT | - 3DCRT: - Phase 1: 40–46 Gy to PTV/PTV-elective using 4–7 fields - Phase 2: Boost 20–26 Gy to pGTV with spinal cord shielding - Total dose: 60–70 Gy | Mix: cisplatin+/-taxane+/-5-FU | OS, PFS, response rate (PR+CR, CR) |
| Hoa Yang_IMRT | 2017 | China | R | 79.2 | IMRT | - IMRT: Single-isocenter, coplanar plan with 5–9 dynamic MLC fields - Total dose: 60–70 Gy | Mix: cisplatin+/-taxane+/-5-FU | OS, PFS, response rate (PR+CR, CR) |
| Yujin Xu | 2016 | China | R | 75 | 3DCRT | - Details of dose escalation: NR - Some SIB, some sequential | S1/Paclitaxel+cisplatin/Docetaxel+cisplatin/Cisplatin+5-FU | OS, PFS, LRPFS, response rate (PR+CR, CR), any failure, LR failure, distant failure |
| Mohan Suntharalingam_Cetux | 2017 | USA | P | 59.5 | 3DCRT |  | Weekly cisplatin+paclitaxel+cetuximab | OS, response rate (PR+CR, CR), LR failure, distant failure |
| Mohan Suntharalingam | 2017 | USA | P | 59.5 | 3DCRT |  | Weekly cisplatin+paclitaxel | OS, response rate (PR+CR, CR), LR failure, distant failure |
| Matthias Felix Haefner_3DCRT | 2017 | Germany | R | 64.8 | 3DCRT |  | Cisplatin + 5-FU | OS, PFS |
| Matthias Felix Haefner_IMRT | 2017 | Germany | R | 68.2 | IMRT |  | Cisplatin + 5-FU | OS, PFS |
| Chia-Lun Chang_SD | 2017 | Taiwan | R | 59.5 | IMRT |  | NR | OS |
| Chia-Lun Chang_HD | 2017 | Taiwan | R | 79.9 | IMRT | Details of dose escalation: NR | NR | OS |
| Yuxia Deng_SD | 2017 | China | R | 59.5 | IMRT/3DCRT |  | Cisplatin+5-FU+Doce/Pacli | OS, PFS, response rate (PR+CR, CR) |
| Yuxia Deng_HD | 2017 | China | R | 70.2 | IMRT/3DCRT | - PTV: ≥59.4 Gy - PTV=CTV (CTV-T+CTV-N)+0.5 cm margin - CTV-N: 0.5 to 0.8 cm expansion around the GTV-N and some patients also covered the regional nodal regions. | Cisplatin+5-FU+Doce/Pacli | OS, PFS, response rate (PR+CR, CR) |
| A Kumabe | 2018 | Japan | R | 72 | 2D/3DCRT | - Initial phase: 46 Gy using AP-PA opposed fields to primary tumor, metastatic lymph nodes with a 3.0 to 5.0 cm craniocaudal margin and a 2.0 to 3.0 cm radial margin - Escalation phase: boost of 14 Gy using two-port oblique opposed field | Cisplatin + 5-FU | OS, PFS, response rate (PR+CR, CR), any failure, LR failure, distant failure |
| Jun Won Kim | 2018 | Korea | R | 63 |  |  | FU+cisplatin or carboplatin | OS, PFS, LRPFS, response rate (CR), any failure, LR failure, distant failure |
| Nayan_SD | 2018 | India | P | 59.5 | 3DCRT |  | Cisplatin + 5-FU | OS, PFS, response rate (PR+CR, CR), any failure, LR failure, distant failure |
| Nayan_HD | 2018 | India | P | 76.5 | 3DCRT | - Initial phase: 39.6 Gy (22 fractions) using AP-PA fields to the primary tumor with a craniocaudal margin of at least 5 cm and circumferential margin of 2 cm to the tumor. - Escalation phase: primary tumor with a 2–3 cm craniocaudal margin to a total dose of 64.8 Gy | Cisplatin + 5-FU | OS, PFS, response rate (PR+CR, CR), any failure, LR failure, distant failure |
| Chao-Yueh Fan_SD | 2018 | Taiwan | R | 60 | IMRT/VMAT/3DCRT |  | Cisplatin + 5-FU | OS, PFS, LRPFS |
| Chao-Yueh Fan_HD | 2018 | Taiwan | R | 79.2 | IMRT/VMAT/3DCRT | Sequential Boost:   - CTV and regional nodes received 41.4–50.4 Gy in 1.8–2 Gy/fractions - Followed by a boost to GTV to 50.4–70 Gy   Simultaneous Integrated Boost (SIB):   - Delivered via VMAT or IMRT - GTV: 50.4–70 Gy - CTV/regional nodes: 45–60 Gy concurrently | Cisplatin + 5-FU | OS, PFS, LRPFS |
| Tae Hyung Kim_SD | 2019 | Korea | R | 59.5 | 2D/3DCRT/IMRT |  | Tri-weekly FU + cisplatin | OS, PFS, LRPFS, LR failure, distant failure |
| Tae Hyung Kim_HD | 2019 | Korea | R | 74.3 | 2D/3DCRT/IMRT | 3DCRT:   - Initial phase: 30.6-45 Gy using AP-PA fields to CTV (GTV-T and GTV-N with regional nodal areas) - Escalation phase: Cone down and boost to the CTV-T and CTV-N without regional nodal areas   IMRT: SIB technique   - PTV1: GTV+0.7 cm margin: 2.1 Gy per fraction - PTV2: CTV+0.7 cm margin: 1.7 Gy per fraction | Tri-weekly FU + cisplatin | OS, PFS, LRPFS, LR failure, distant failure |
| Chen Li | 2019 | China | R | 72.7 | IMRT/VMAT | SIB technique:   - Prophylactic PTV: 50.4 Gy/28 fractions - 60.76Gy /2.17Gy /28f (dose level 1). - 61.88Gy /2.21Gy /28f (dose level 2). - 64.12Gy /2.29Gy /28f (dose level 3). | Weekly paclitaxel + nedaplatin | OS, PFS, LRPFS |
| Yun Chen_CF | 2019 | China | P | 72.2 | NR |  | Cisplatin + 5-FU | OS, PFS, any failure, LR failure, distant failure |
| Yun Chen_PF | 2019 | China | P | 72.2 | NR | - 61.2 Gy in 34 fractions (5 days per week at 1.8 Gy/d) - Details of dose escalation: NR | Paclitaxel + 5-FU | OS, PFS, any failure, LR failure, distant failure |
| Judith de Vos-Geelen_SD_C | 2020 | Netherlands | R | 59.5 | IMRT/VMAT/3DCRT |  | Cisplatin-based | OS, response rate (PR+CR, CR) |
| Judith de Vos-Geelen_SD_PC | 2020 | Netherlands | R | 59.5 | IMRT/VMAT/3DCRT |  | Weekly paclitaxel + carboplatin | OS, response rate (PR+CR, CR) |
| Judith de Vos-Geelen_HD_PC | 2020 | Netherlands | R | 73.9 | IMRT/VMAT/3DCRT | - ≥50.4 Gy to PTV (CTV + margin up to 1cm) - CTV=GTV+elective nodal areas | Weekly paclitaxel + carboplatin | OS, response rate (PR+CR, CR) |
| Judith de Vos-Geelen_HD_C | 2020 | Netherlands | R | 84 | IMRT/VMAT/3DCRT | - ≥50.4 Gy to PTV (CTV + margin up to 1cm) - CTV=GTV+elective nodal areas | Cisplatin-based | OS, response rate (PR+CR, CR) |
| Gabriella Alexandersson von Döbeln | 2020 | Sweden | P | 60 | 3DCRT |  | Weekly 5-FU+Oxaliplatin+cetuximab | OS, PFS, LRPFS, response rate (PR+CR, CR), any failure, LR failure, distant failure |
| Chia-Chin Li_SD | 2021 | Taiwan | R | 60 | IMRT |  | NR | OS |
| Chia-Chin Li_HD | 2021 | Taiwan | R | 79.2 | IMRT | Details of dose escalation: NR | NR | OS |
| Maarten C C M Hulshof_SD | 2021 | Netherlands | P | 59.5 | IMRT |  | Weekly paclitaxel + carboplatin | OS, LRPFS |
| Maarten C C M Hulshof_HD | 2021 | Netherlands | P | 74.7 | IMRT | SIB technique:   - Primary tumor: 61.6 Gy/2.2 Gy per fraction | Weekly paclitaxel + carboplatin | OS, LRPFS |
| Yujin Xu_SD | 2022 | China | P | 60 | IMRT |  | Weekly docetaxel + cisplatin | OS, PFS, LRPFS, response rate (PR+CR), any failure, LR failure, distant failure |
| Yujin Xu_HD | 2022 | China | P | 72 | IMRT | - PTV (0.5 cm margin around the CTV-T and CTV-N): 60 Gy.   CTV-N covers regional drainage areas according to the tumor location. | Weekly docetaxel + cisplatin | OS, PFS, LRPFS, response rate (PR+CR), any failure, LR failure, distant failure |
| Huiping Zhu_SD | 2022 | China | R | 59.5 | IMRT |  | S1/Docetaxel + Nedaplatin | OS, PFS, response rate (PR+CR, CR), any failure, LR failure, distant failure |
| Huiping Zhu_HD | 2022 | China | R | 72 | IMRT | - Initial phase: 45-50.4 Gy/1.8 Gy to the elective nodal regions - Escalation phase: boost of primary tumor and metastatic nodes to 60 Gy/2 Gy | S1/Docetaxel + Nedaplatin | OS, PFS, response rate (PR+CR, CR), any failure, LR failure, distant failure |
| Jing You_SD | 2023 | China | P | 59.5 | IMRT |  | Weekly paclitaxel + carboplatin | OS, PFS, LRPFS, any failure, LR failure, distant failure |
| Jing You_HD | 2023 | China | P | 70.1 | IMRT | - 59.4 Gy in 33 fractions - After 23 fractions, CTV margins reduced: - CTVp: +2 cm (longitudinal) - CTVnd: +0.5 cm around involved nodes | Weekly paclitaxel + carboplatin | OS, PFS, LRPFS, any failure, LR failure, distant failure |
| Feihong Xie_SD | 2023 | China | R | 67.2 | IMRT |  | Cisplatin/5-FU or cisplatin/paclitaxel | OS, PFS, LRPFS, response rate (PR+CR) |
| Feihong Xie_HD | 2023 | China | R | 74.4 | IMRT | - 1.8 Gy or 2.0 Gy per fraction 5 days per week using IMRT. - Details of dose escalation: NR | Cisplatin/5-FU or cisplatin/paclitaxel | OS, PFS, LRPFS, response rate (PR+CR) |
| HD=high dose; SD=standard dose; CRT=chemoradiotherapy; P=prospective; R=retrospective; BED=biologically effective dose; 3DCRT=3-dimensional conformal radiotherapy; IMRT=intensity modulated radiotherapy; OS=overall survival; PFS=progression-free survival; LRPFS=locoregional progression-free survival; PR+CR=partial response + complete response; CR=complete response; LR failure=locoregional failure; NR=not reported; PC=protracted continuous; GTV=gross tumor volume; CTV=clinical target volume; PTV=planning target volume; SIB=simultaneous integrated boost. | | | | | | | |  |

| **Supplementary Table S6. Newcastle-Ottawa Scale quality assessment scale for cohort studies.** | | | | | | | | | |
| --- | --- | --- | --- | --- | --- | --- | --- | --- | --- |
| **Author, year** | **Representativeness of the Exposed Cohort** | **Selection of the Non-Exposed Cohort** | **Ascertainment of Exposure** | **Demonstration That Outcome of Interest Was Not Present at Start of Study** | **Comparability of Cohorts on the Basis of the Design or Analysis** | **Assessment of Outcome** | **Was Follow-Up Long Enough for Outcomes to Occur** | **Adequacy of Follow Up of Cohorts** | **Total points** |
| Han, 2012 | * | * | * | * | ** | * | * | * | 9 |
| Li, 2019 | * | * | * | * | ** | * |  | * | 8 |
| Zhu, 2012 | * | * | * | * | ** | * | * | * | 9 |
| Zhu, 2022 | * | * | * | * | ** | * |  | * | 8 |
| Ge, 2015 | * | * | * | * | ** | * | * | * | 9 |
| Xu, 2016 | * | * | * | * | ** | * | * | * | 9 |
| He, 2016 | * | * | * | * | ** | * |  | * | 8 |
| Yang, 2017 | * | * | * | * | ** | * | * | * | 9 |
| Meng, 2013 | * |  | * | * | ** | * | * | * | 8 |
| Deng, 2017 | * | * | * | * | ** | * | * | * | 9 |
| Clavier, 2013 | * | * | * | * | ** | * |  | * | 8 |
| Haefner, 2017 | * | * | * | * | ** | * | * | * | 9 |
| Roeder, 2014 | * |  | * | * | ** | * | * | * | 8 |
| Nayan, 2018 |  | * | * | * | ** | * |  | * | 7 |
| Ishida, 2004 | * |  | * | * | ** | * |  | * | 7 |
| Takagawa, 2009 | * |  | * | * | ** | * |  | * | 7 |
| Kumabe, 2018 | * |  | * | * | ** | * | * | * | 8 |
| Nishimura, 2012 | * | * | * | * | ** | * | * | * | 9 |
| Higuchi, 2014 | * |  | * | * | ** | * | * | * | 8 |
| Kim, 2018 | * |  | * | * | ** | * | * | * | 8 |
| Suh, 2014 | * | * | * | * | ** | * | * | * | 9 |
| Geelen, 2020 | * | * | * | * | ** | * | * | * | 9 |
| Versteijne, 2015 | * |  | * | * | ** | * |  | * | 7 |
| Hurmuzlu, 2010 | * |  | * | * | ** | * | * | * | 8 |
| Fan, 2018 | * | * | * | * | ** | * | * | * | 9 |
| Li, 2021 | * | * | * | * | ** | * | * | * | 9 |
| Chen, 2016 | * | * | * | * | ** | * | * | * | 9 |
| Chang, 2017 | * | * | * | * | ** | * | * | * | 9 |
| Freilich, 2014 | * | * | * | * | ** | * |  | * | 8 |
| Kato, 2013 | * |  | * | * | ** | * | * | * | 8 |
| Kim, 2019 | * | * | * | * | ** | * | * | * | 9 |
| Döbeln, 2020 | * | * | * | * | ** | * | * | * | 9 |
| Xie, 2023 | * | * | * | * | ** | * | * | * | 9 |
| Total score: 0-3=low quality; 4-6=intermediate quality; 7-9=high quality | | | | | | | | | |

| **Supplementary Table S7. Grading of Recommendations Assessment, Development, and Evaluation.** | | | | | | | |
| --- | --- | --- | --- | --- | --- | --- | --- |
| No. of studies | Design | Risk of bias | Inconsistency | Indirectness | Imprecision | Others | Certainty |
| Primary outcome: HD vs SD radiotherapy in terms of overall survival benefits | | | | | | | |
| 27 | Retrospective | Serious (-1) | Serious (-1) | Not serious | Not serious | Nil | ⊕⊕◯◯ Low |
| Primary outcome: HD vs SD radiotherapy in terms of overall survival benefits | | | | | | | |
| 14 | Prospective | Not serious | Serious (-1) | Not serious | Not serious | Nil | ⊕⊕⊕◯ Moderate |
| Primary outcome: HD vs SD radiotherapy in terms of progression-free survival benefits | | | | | | | |
| 16 | Retrospective | Serious (-1) | Serious (-1) | Not serious | Not serious | Nil | ⊕◯◯◯ Very Low |
| Primary outcome: HD vs SD radiotherapy in terms of progression-free survival benefits | | | | | | | |
| 7 | Prospective | Not serious | Serious (-1) | Not serious | Not serious | Nil | ⊕⊕◯◯ Low |
| Primary outcome: HD vs SD radiotherapy in terms of locoregional progression-free survival benefits | | | | | | | |
| 10 | Retrospective | Not serious | Not serious | Not serious | Not serious | Nil | ⊕⊕⊕◯ Moderate |
| Primary outcome: HD vs SD radiotherapy in terms of locoregional progression-free survival benefits | | | | | | | |
| 4 | Prospective | Not serious | Not serious | Not serious | Not serious | Nil | ⊕⊕⊕⊕ High |
| HD=high dose; SD=standard dose | | | | | | | |

| **Supplementary Table S8. Differences of survival rate between high and low categorical subgroups.** | | | | | | | | | | | | | |
| --- | --- | --- | --- | --- | --- | --- | --- | --- | --- | --- | --- | --- | --- |
|  | | **OS** | | | | **PFS** | | | | **LRPFS** | | | |
| **Categorical factors** | **Median** | **1year** | **2year** | **3year** | **5year** | **1year** | **2year** | **3year** | **5year** | **1year** | **2year** | **3year** | **5year** |
| Age | 64 | 0.251 | 0.846 | 0.779 | 0.829 | 0.962 | 0.824 | 0.589 | 0.683 | 0.346 | 0.111 | 0.160 | 0.925 |
| Male | 0.80 | 0.424 | 0.246 | 0.438 | 0.308 | 0.638 | 0.217 | 0.042 | 0.335 | 0.850 | 0.863 | 0.834 | 0.433 |
| ECOG≥1 | 0.49 | 0.710 | 0.747 | 0.853 | 0.582 | 0.474 | 0.463 | 0.936 | 0.711 | 0.344 | 0.092 | 0.258 | 0.164 |
| Tumor length | 6 | 0.525 | 0.954 | 0.713 | 0.987 | 0.988 | 0.799 | 0.980 | 0.679 | 0.109 | 0.091 | 0.046 | 0.234 |
| SqCC | 0.87 | 0.306 | 0.140 | 0.132 | 0.062 | 0.713 | 0.613 | 0.374 | 0.769 | 0.789 | 0.612 | 0.725 | 0.720 |
| Middle/lower | 0.67 | 0.576 | 0.810 | 0.345 | 0.285 | 0.676 | 0.998 | 0.881 | 0.499 | 0.109 | 0.028 | 0.160 | 0.711 |
| T3/4 | 0.76 | 0.879 | 0.686 | 0.837 | 0.294 | 0.639 | 0.502 | 0.978 | 0.832 | 0.569 | 0.457 | 0.821 | 0.404 |
| N positivity | 0.66 | 0.354 | 0.227 | 0.197 | 0.944 | 0.200 | 0.661 | 0.248 | 0.401 | 0.341 | 0.272 | 0.341 | 0.025 |
| Dose group | NA | 0.653 | 0.457 | 0.166 | 0.159 | 0.007 | 0.231 | 0.199 | 0.067 | 0.0001 | 0.021 | <0.001 | 0.091 |
| CHT regimen | NA | 0.685 | 0.558 | 0.278 | 0.471 | 0.094 | 0.035 | 0.092 | 0.484 | 0.311 | 0.1087 | 0.104 | 0.269 |
| EOCG=Eastern Cooperative Oncology Group; SqCC = squamous cell carcinoma; CHT=chemotherapy; OS=overall survival; PFS=progression-free survival; LRPFS=locoregional progression-free survival. NA=not applicable. P values are calculated with either Kolmogorov-Smirnov test or Kruskal-Wallis test (for CHT regimen). | | | | | | | | | | | | | |

| **Supplementary Table S9. Between-study heterogeneity τ of each Bayesian model.** | | | | | | |
| --- | --- | --- | --- | --- | --- | --- |
|  | | | **95% CrI** | |  | |
|  | **Estimate of** τ | **Estimated error** | **lower** | **upper** | **Rhat** | **Mean of estimates** |
| OS-1year | 0.68 | 0.10 | 0.51 | 0.92 | 1.00 | 0.6225 |
| OS-2year | 0.59 | 0.10 | 0.42 | 1.00 | 1.00 |  |
| OS-3year | 0.53 | 0.09 | 0.37 | 0.74 | 1.00 |  |
| OS-5year | 0.69 | 0.19 | 0.38 | 1.15 | 1.00 |  |
| PFS-1year | 0.48 | 0.11 | 0.31 | 0.74 | 1.00 | 0.5025 |
| PFS-2year | 0.47 | 0.13 | 0.26 | 0.75 | 1.01 |  |
| PFS-3year | 0.35 | 0.18 | 0.04 | 0.74 | 1.00 |  |
| PFS-5year | 0.71 | 0.63 | 0.03 | 2.37 | 1.01 |  |
| LRPFS-1year | 0.43 | 0.17 | 0.17 | 0.86 | 1.00 | 0.5125 |
| LRPFS-2year | 0.54 | 0.17 | 0.28 | 0.95 | 1.00 |  |
| LRPFS-3year | 0.37 | 0.20 | 0.05 | 0.84 | 1.00 |  |
| LRPFS-5year | 0.71 | 0.68 | 0.04 | 2.21 | 1.00 |  |
| OS=overall survival; PFS=progression-free year; LRPFS=locoregional progression-free survival; CrI=credible interval; Rhat= R-hat convergence diagnostic. | | | | | | |

| **Supplementary Table S10. Estimate of effects with radiation dose escalation on survival outcomes.** | | | | | | |
| --- | --- | --- | --- | --- | --- | --- |
|  | | | **95% CrI** | |  | |
|  | **Estimate** | **Estimated error** | **lower** | **upper** | **Rhat** | **Bayes factor** |
| OS-1year | 0.052 | 0.029 | -0.006 | 0.110 | 1.000 | 2.77 |
| OS-2year | 0.060 | 0.033 | -0.006 | 0.125 | 0.999 | 2.74 |
| OS-3year | 0.070 | 0.035 | 0.0001 | 0.139 | 1.000 | 1.93 |
| OS-5year | 0.090 | 0.055 | -0.017 | 0.196 | 1.003 | 1.55 |
| PFS-1year | 0.141 | 0.039 | 0.063 | 0.217 | 1.000 | 14.7 |
| PFS-2year | 0.093 | 0.044 | 0.005 | 0.179 | 0.999 | 2.79 |
| PFS-3year | 0.042 | 0.042 | -0.040 | 0.125 | 0.999 | 1.15 |
| PFS-5year | 0.060 | 0.089 | -0.113 | 0.233 | 1.001 | 1.02 |
| LRPFS-1year | 0.186 | 0.038 | 0.107 | 0.261 | 1.002 | 13.6 |
| LRPFS-2year | 0.161 | 0.052 | 0.064 | 0.262 | 0.999 | 5.08 |
| LRPFS-3year | 0.165 | 0.048 | 0.075 | 0.259 | 0.999 | 2.01 |
| LRPFS-5year | 0.188 | 0.093 | 0.003 | 0.374 | 1.002 | 1.59 |
| OS=overall survival; PFS=progression-free year; LRPFS=locoregional progression-free survival; CrI=credible interval; Rhat= R-hat convergence diagnostic. | | | | | | |

| **Supplementary Table S11. Sensitivity analysis.** | | | | | | |
| --- | --- | --- | --- | --- | --- | --- |
| **China** | | | | | | |
|  | | | **95% CrI** | |  | |
|  | **Estimate** | **Estimated error** | **lower** | **upper** | **Rhat** | **Bayes factor** |
| OS-1year | 0.038 | 0.031 | -0.022 | 0.101 | 0.999 | 1.87 |
| OS-2year | 0.129 | 0.049 | 0.033 | 0.226 | 1.000 | 1.74 |
| OS-3year | 0.104 | 0.045 | 0.012 | 0.192 | 1.000 | 4.37 |
| OS-5year | 0.129 | 0.080 | -0.029 | 0.287 | 1.000 | 1.56 |
| PFS-1year | 0.154 | 0.045 | 0.066 | 0.243 | 1.000 | 24.52 |
| PFS-2year | 0.159 | 0.067 | 0.029 | 0.287 | 1.002 | 5.01 |
| PFS-3year | 0.113 | 0.079 | -0.043 | 0.266 | 1.000 | 1.36 |
| PFS-5year | 0.002 | 0.103 | -0.198 | 0.205 | 1.000 | 1.05 |
| LRPFS-1year | 0.137 | 0.039 | 0.057 | 0.214 | 1.001 | 16.57 |
| LRPFS-2year | 0.157 | 0.058 | 0.045 | 0.272 | 1.000 | 4.95 |
| LRPFS-3year | 0.171 | 0.066 | 0.042 | 0.298 | 1.000 | 5.89 |
| LRPFS-5year | 0.051 | 0.132 | -0.206 | 0.310 | 1.005 | 1.02 |
| **Non-China** | | | | | | |
|  | | | **95% CrI** | |  | |
|  | **Estimate** | **Estimated error** | **lower** | **upper** | **Rhat** | **Bayes factor** |
| OS-1year | 0.031 | 0.039 | -0.042 | 0.109 | 1.001 | 0.976 |
| OS-2year | 0.012 | 0.043 | -0.073 | 0.094 | 1.001 | 0.760 |
| OS-3year | 0.066 | 0.049 | -0.029 | 0.164 | 1.000 | 1.67 |
| OS-5year | 0.092 | 0.069 | -0.043 | 0.224 | 1.001 | 1.29 |
| PFS-1year | 0.239 | 0.086 | 0.072 | 0.408 | 1.002 | 27.9 |
| PFS-2year | 0.146 | 0.079 | -0.011 | 0.301 | 1.001 | 2.74 |
| PFS-3year | 0.098 | 0.098 | -0.090 | 0.291 | 1.000 | 1.17 |
| PFS-5year | 0.248 | 0.153 | -0.059 | 0.547 | 1.001 | 1.33 |
| LRPFS-1year | 0.253 | 0.056 | 0.146 | 0.36 | 1.004 | 758.9 |
| LRPFS-2year | 0.198 | 0.068 | 0.065 | 0.328 | 1.000 | 6.26 |
| LRPFS-3year | 0.211 | 0.058 | 0.093 | 0.323 | 1.001 | 5.56 |
| LRPFS-5year | 0.257 | 0.092 | 0.078 | 0.438 | 1.002 | 2.07 |
| **Prospective** | | | | | | |
|  | | | **95% CrI** | |  | |
|  | **Estimate** | **Estimated error** | **lower** | **upper** | **Rhat** | **Bayes factor** |
| OS-1year | 0.073 | 0.049 | -0.027 | 0.17 | 1.000 | 2.04 |
| OS-2year | 0.088 | 0.054 | -0.020 | 0.193 | 1.002 | 1.98 |
| OS-3year | 0.106 | 0.047 | 0.015 | 0.196 | 1.001 | 3.02 |
| OS-5year | 0.003 | 0.084 | -0.161 | 0.169 | 1.000 | 0.938 |
| PFS-1year | 0.215 | 0.057 | 0.102 | 0.329 | 1.000 | 17.7 |
| PFS-2year | 0.124 | 0.065 | -0.001 | 0.254 | 1.001 | 2.05 |
| PFS-3year | 0.059 | 0.069 | -0.074 | 0.193 | 1.003 | 1.07 |
| PFS-5year | -0.011 | 0.102 | -0.214 | 0.186 | 1.004 | 0.926 |
| LRPFS-1year | 0.128 | 0.039 | 0.052 | 0.203 | 1.000 | 15.2 |
| LRPFS-2year | 0.066 | 0.049 | -0.029 | 0.161 | 1.001 | 1.88 |
| LRPFS-3year | 0.072 | 0.060 | -0.049 | 0.188 | 1.000 | 1.53 |
| LRPFS-5year | 0.049 | 0.113 | -0.172 | 0.273 | 1.001 | 1.16 |
| **Retrospective** | | | | | | |
|  | | | **95% CrI** | |  | |
|  | **Estimate** | **Estimated error** | **lower** | **upper** | **Rhat** | **Bayes factor** |
| OS-1year | 0.072 | 0.037 | -0.003 | 0.145 | 1.001 | 3.95 |
| OS-2year | 0.073 | 0.041 | -0.004 | 0.154 | 1.002 | 3.76 |
| OS-3year | 0.083 | 0.041 | 0.002 | 0.164 | 1.001 | 2.77 |
| OS-5year | 0.104 | 0.065 | -0.024 | 0.232 | 1.001 | 1.28 |
| PFS-1year | 0.142 | 0.050 | 0.041 | 0.239 | 1.001 | 18.2 |
| PFS-2year | 0.086 | 0.051 | -0.016 | 0.190 | 1.001 | 2.08 |
| PFS-3year | 0.024 | 0.057 | -0.088 | 0.136 | 1.000 | 0.958 |
| PFS-5year | -0.037 | 0.115 | -0.261 | 0.181 | 1.000 | 1.01 |
| LRPFS-1year | 0.259 | 0.057 | 0.143 | 0.371 | 1.000 | 50.3 |
| LRPFS-2year | 0.233 | 0.077 | 0.083 | 0.382 | 1.000 | 9.05 |
| LRPFS-3year | 0.245 | 0.060 | 0.127 | 0.361 | 1.000 | 19.6 |
| LRPFS-5year | 0.292 | 0.097 | 0.103 | 0.485 | 1.002 | 1.03 |
| **Reported number at risk** | | | | | | |
|  | | | **95% CrI** | |  | |
|  | **Estimate** | **Estimated error** | **lower** | **upper** | **Rhat** | **Bayes factor** |
| OS-1year | 0.049 | 0.027 | -0.005 | 0.108 | 1.001 | 3.23 |
| OS-2year | 0.055 | 0.031 | -0.006 | 0.127 | 1.000 | 2.71 |
| OS-3year | 0.065 | 0.032 | 0.0001 | 0.144 | 1.000 | 2.15 |
| OS-5year | 0.072 | 0.051 | -0.014 | 0.199 | 1.002 | 1.02 |
| PFS-1year | 0.162 | 0.032 | 0.061 | 0.225 | 1.001 | 17.7 |
| PFS-2year | 0.098 | 0.037 | 0.004 | 0.182 | 0.999 | 3.45 |
| PFS-3year | 0.040 | 0.048 | -0.027 | 0.137 | 1.002 | 1.07 |
| PFS-5year | 0.055 | 0.072 | -0.106 | 0.245 | 1.001 | 0.91 |
| LRPFS-1year | 0.173 | 0.032 | 0.112 | 0.289 | 1.001 | 15.7 |
| LRPFS-2year | 0.152 | 0.041 | 0.054 | 0.278 | 0.999 | 6.37 |
| LRPFS-3year | 0.144 | 0.044 | 0.071 | 0.267 | 1.001 | 2.24 |
| LRPFS-5year | 0.179 | 0.082 | 0.001 | 0.394 | 1.001 | 1.61 |
| **Estimated number at risk** | | | | | | |
|  | | | **95% CrI** | |  | |
|  | **Estimate** | **Estimated error** | **lower** | **upper** | **Rhat** | **Bayes factor** |
| OS-1year | 0.054 | 0.032 | -0.003 | 0.125 | 0.999 | 2.87 |
| OS-2year | 0.049 | 0.025 | -0.005 | 0.132 | 1.001 | 2.64 |
| OS-3year | 0.055 | 0.030 | 0.0001 | 0.154 | 1.000 | 2.11 |
| OS-5year | 0.069 | 0.051 | -0.014 | 0.204 | 1.002 | 1.02 |
| PFS-1year | 0.162 | 0.032 | 0.061 | 0.225 | 1.001 | 18.9 |
| PFS-2year | 0.088 | 0.032 | 0.003 | 0.192 | 0.999 | 4.46 |
| PFS-3year | 0.032 | 0.041 | -0.022 | 0.145 | 1.001 | 0.98 |
| PFS-5year | 0.042 | 0.069 | -0.101 | 0.223 | 1.001 | 0.88 |
| LRPFS-1year | 0.167 | 0.028 | 0.114 | 0.276 | 1.001 | 17.8 |
| LRPFS-2year | 0.141 | 0.032 | 0.045 | 0.282 | 1.002 | 5.87 |
| LRPFS-3year | 0.132 | 0.041 | 0.069 | 0.269 | 1.001 | 1.89 |
| LRPFS-5year | 0.172 | 0.076 | 0.001 | 0.432 | 1.001 | 1.45 |
| OS=overall survival; PFS=progression-free year; LRPFS=locoregional progression-free survival; CrI=credible interval; Rhat= R-hat convergence diagnostic. | | | | | | |

| **Supplementary Table S12. Bayesian subgroup analysis.** | | | | | | |
| --- | --- | --- | --- | --- | --- | --- |
| **SqCC** | | | | | | |
|  | | | **95% CrI** | |  | |
|  | **Estimate** | **Estimated error** | **lower** | **upper** | **Rhat** | **Bayes factor** |
| OS-1year | 0.096 | 0.041 | 0.018 | 0.176 | 0.999 | 14.0 |
| OS-2year | 0.103 | 0.043 | 0.019 | 0.189 | 1.000 | 6.33 |
| OS-3year | 0.104 | 0.049 | 0.005 | 0.201 | 1.001 | 2.9 |
| OS-5year | 0.100 | 0.072 | -0.041 | 0.243 | 1.000 | 1.24 |
| PFS-1year | 0.173 | 0.047 | 0.076 | 0.265 | 1.000 | 14.9 |
| PFS-2year | 0.110 | 0.056 | 0.001 | 0.220 | 1.000 | 2.48 |
| PFS-3year | 0.055 | 0.063 | -0.068 | 0.181 | 1.000 | 1.11 |
| PFS-5year | 0.045 | 0.093 | -0.136 | 0.225 | 1.001 | 0.993 |
| LRPFS-1year | 0.232 | 0.058 | 0.117 | 0.347 | 1.001 | 9.91 |
| LRPFS-2year | 0.176 | 0.075 | 0.027 | 0.328 | 1.000 | 3.94 |
| LRPFS-3year | 0.177 | 0.059 | 0.061 | 0.291 | 1.000 | 2.03 |
| LRPFS-5year | 0.177 | 0.059 | 0.061 | 0.291 | 1.000 | 1.61 |
| **Middle/lower** | | | | | | |
|  | | | **95% CrI** | |  | |
|  | **Estimate** | **Estimated error** | **lower** | **upper** | **Rhat** | **Bayes factor** |
| OS-1year | 0.075 | 0.058 | -0.039 | 0.192 | 0.999 | 1.79 |
| OS-2year | 0.111 | 0.063 | -0.012 | 0.230 | 1.002 | 1.96 |
| OS-3year | 0.128 | 0.098 | -0.062 | 0.317 | 1.002 | 2.18 |
| OS-5year | NA | NA | NA | NA | NA | NA |
| PFS-1year | 0.122 | 0.065 | -0.004 | 0.250 | 1.000 | 3.07 |
| PFS-2year | 0.101 | 0.072 | -0.041 | 0.241 | 1.000 | 1.57 |
| PFS-3year | 0.009 | 0.086 | -0.160 | 0.177 | 1.001 | 0.987 |
| PFS-5year | NA | NA | NA | NA | NA | NA |
| LRPFS-1year | NA | NA | NA | NA | NA | NA |
| LRPFS-2year | 0.265 | 0.134 | 0.009 | 0.535 | 1.002 | 2.88 |
| LRPFS-3year | NA | NA | NA | NA | NA | NA |
| LRPFS-5year | NA | NA | NA | NA | NA | NA |
| OS=overall survival; PFS=progression-free year; LRPFS=locoregional progression-free survival; CrI=credible interval; Rhat= R-hat convergence diagnostic. | | | | | | |

| **Supplementary Table S13. Bayesian subgroup analysis for radiation technique.** | | | | | | |
| --- | --- | --- | --- | --- | --- | --- |
| **IMRT/VMAT** | | | | | | |
|  | | | **95% CrI** | |  | |
|  | **Estimate** | **Estimated error** | **lower** | **upper** | **Rhat** | **Bayes factor** |
| OS-1year | 0.074 | 0.044 | -0.010 | 0.162 | 1.000 | 2.97 |
| OS-2year | 0.071 | 0.051 | -0.027 | 0.169 | 1.000 | NC |
| OS-3year | 0.051 | 0.054 | -0.054 | 0.156 | 0.999 | NC |
| OS-5year | -0.048 | 0.079 | -0.201 | 0.110 | 0.999 | 1.07 |
| PFS-1year | 0.146 | 0.056 | 0.035 | 0.258 | 1.000 | NC |
| PFS-2year | 0.101 | 0.053 | -0.003 | 0.205 | 1.001 | 1.90 |
| PFS-3year | 0.035 | 0.061 | -0.082 | 0.152 | 1.000 | 0.923 |
| PFS-5year | NA | | | | | |
| LRPFS-1year | 0.117 | 0.036 | 0.049 | 0.189 | 1.004 | 2.93 |
| LRPFS-2year | 0.123 | 0.056 | 0.0155 | 0.235 | 1.002 | 2.15 |
| LRPFS-3year | 0.132 | 0.060 | 0.015 | 0.248 | 1.001 | 1.86 |
| LRPFS-5year | 0.008 | 0.109 | -0.198 | 0.223 | 1.001 | 0.972 |
| OS=overall survival; PFS=progression-free year; LRPFS=locoregional progression-free survival; CrI=credible interval; Rhat= R-hat convergence diagnostic; NA=not-applicable; NC=non-convergence | | | | | | |

| **Supplementary Table S14. Bayesian subgroup analysis for SqCC in different demographic regions.** | | | | | | | |
| --- | --- | --- | --- | --- | --- | --- | --- |
|  | | | | **95% CrI** | |  | |
|  | **Region** | **Estimate** | **Estimated error** | **lower** | **upper** | **Rhat** | **Bayes factor** |
| OS-1year | Western | 0.052 | 0.057 | -0.062 | 0.164 | 1.001 | 0.357 |
|  | Asia | 0.137 | 0.049 | 0.042 | 0.233 | 1.001 | 30.3 |
| OS-2year | Western | -0.003 | 0.051 | -0.104 | 0.095 | 1.000 | 0.241 |
|  | Asia | 0.159 | 0.056 | 0.044 | 0.269 | 1.001 | 13.56 |
| OS-3year | Western | 0.035 | 0.066 | -0.093 | 0.163 | 1.002 | 0.912 |
|  | Asia | 0.131 | 0.061 | 0.011 | 0.251 | 1.000 | 3.83 |
| OS-5year | Western | 0.051 | 0.133 | -0.202 | 0.311 | 1.002 | 1.04 |
|  | Asia | 0.064 | 0.069 | -0.069 | 0.200 | 1.000 | 1.25 |
| PFS-1year | Western | 0.195 | 0.082 | 0.036 | 0.354 | 1.000 | 17.63 |
|  | Asia | 0.212 | 0.057 | 0.098 | 0.325 | 1.003 | 57.88 |
| PFS-2year | Western | -0.116 | 0.062 | -0.238 | 0.007 | 0.999 | 0.156 |
|  | Asia | 0.112 | 0.065 | -0.014 | 0.242 | 1.000 | 2.31 |
| PFS-3year | Western | -0.161 | 0.081 | -0.317 | 0.002 | 0.999 | 0.623 |
|  | Asia | 0.0682 | 0.077 | -0.085 | 0.222 | 1.000 | 1.14 |
| PFS-5year | Western | -0.148 | 0.163 | -0.469 | 0.176 | 0.999 | 0.914 |
|  | Asia | 0.031 | 0.096 | -0.166 | 0.215 | 0.999 | 0.978 |
| LRPFS-1year | Western | 0.543 | 0.152 | 0.246 | 0.842 | 1.000 | 299 |
|  | Asia | 0.174 | 0.044 | 0.088 | 0.261 | 1.000 | 14.4 |
| LRPFS-2year | Western | 0.042 | 0.127 | -0.202 | 0.299 | 0.999 | 0.223 |
|  | Asia | 0.169 | 0.054 | 0.064 | 0.274 | 1.002 | 2.8 |
| LRPFS-3year | Western | -0.046 | 0.144 | -0.331 | 0.223 | 1.000 | 0.247 |
|  | Asia | 0.168 | 0.064 | 0.046 | 0.292 | 0.168 | 2.08 |
| LRPFS-5year | Western | NA | | | | | |
|  | Asia | NA | | | | | |
| OS=overall survival; PFS=progression-free year; LRPFS=locoregional progression-free survival; CrI=credible interval; Rhat= R-hat convergence diagnostic; NA=not applicable due to insufficient adjusting factors | | | | | | | |

| **Supplementary Table S15. Differences of clinical factors between Asia and Western countries with high proportion of SqCC (threshold=50%) in the subgroup of HD.** | |
| --- | --- |
| **Factors** | **P value** |
| Age (year) | 0.320 |
| Male (%) | 0.682 |
| ECOG PS ≥1 | 0.055 |
| Middle/lower (%) | 0.276 |
| Tumor length (cm) | 0.145 |
| T 3/4 (%) | 0.132 |
| N+ (%) | 0.469 |
| PR+CR (%) | 0.991 |
| CR (%) | 0.412 |
| Any failure (%) | 0.259 |
| LR failure (%)(%) | 0.571 |
| Distant failure (%) | 0.411 |
| HD=high dose; ECOG PS= ECOG PS=Eastern Cooperative Oncology Group (ECOG) performance status; N=lymph node metastasis; PR=partial response; CR=complete response; LR=locoregional | |

| **Supplementary Table S16. Difference of response rates and failure rates between HD and SD.** | | | | | | | |
| --- | --- | --- | --- | --- | --- | --- | --- |
|  | **Subgroup** | **No. of studies** | **No. of patients** | **Estimated proportion** | **95% CI** | **I^2^ (%)** | **P (test of subgroup difference)** |
| PR+CR | HD | 8 | 928 | 0.819 | 0.732; 0.884 | 74.8 | 0.445 |
|  | SD |  |  | 0.771 | 0.653; 0.858 | 82.0 |  |
| CR | HD | 7 | 578 | 0.461 | 0.276; 0.658 | 90.7 | 0.072 |
|  | SD |  |  | 0.651 | 0.591; 0.707 | 0 |  |
| Any failure | HD | 13 | 1734 | 0.498 | 0.379; 0.616 | 86.9 | 0.681 |
|  | SD |  |  | 0.444 | 0.242; 0.666 | 91.7 |  |
| LR failure | HD | 16 | 2542 | 0.236 | 0.167; 0.322 | 82.4 | 0.489 |
|  | SD |  |  | 0.283 | 0.185; 0.406 | 91.3 |  |
| Distant failure | HD | 17 | 2301 | 0.186 | 0.151; 0.228 | 64.5 | 0.268 |
|  | SD |  |  | 0.141 | 0.089; 0.217 | 82.6 |  |
| HD=high dose; SD=standard dose; PR=partial response; CR=complete response; LR=locoregional; RP=radiation pneumonitis; CI=confidence interval | | | | | | | |

| **Supplementary Table S17. Difference of any-grade toxicities between HD and SD.** | | | | | | | |
| --- | --- | --- | --- | --- | --- | --- | --- |
|  | **Subgroup** | **No. of studies** | **No. of patients** | **Estimated proportion** | **95% CI** | **I^2^ (%)** | **P (test of subgroup difference)** |
| Anemia | HD | 10 | 1065 | 0.645 | 0.244; 0.911 | 91.7 | 0.583 |
|  | SD |  |  | 0.504 | 0.087; 0.915 | 93.2 |  |
| Leukopenia | HD | 8 | 707 | 0.839 | 0.722; 0.913 | 74.0 | 0.534 |
|  | SD |  |  | 0.757 | 0.418; 0.931 | 88.9 |  |
| Neutropenia | HD | 9 | 957 | 0.751 | 0.292; 0.957 | 92.3 | 0.378 |
|  | SD |  |  | 0.477 | 0.137; 0.841 | 92.9 |  |
| Thrombocytopenia | HD | 8 | 890 | 0.264 | 0.150; 0.419 | 87.6 | 0.941 |
|  | SD |  |  | 0.249 | 0.049; 0.681 | 94.1 |  |
| Fatigue | HD | 2 | 199 | 0.452 | 0.368; 0.539 | 0 | 0.186 |
|  | SD |  |  | 0.356 | 0.255; 0.472 | NA |  |
| Nausea | HD | 6 | 766 | 0.356 | 0.292; 0.425 | 65.7 | 0.606 |
|  | SD |  |  | 0.246 | 0.043; 0.704 | 92.8 |  |
| Dysphagia | HD | 7 | 928 | 0.318 | 0.144; 0.563 | 89.5 | 0.206 |
|  | SD |  |  | 0.489 | 0.374; 0.606 | 84.6 |  |
| Heart | HD | 3 | 591 | 0.035 | 0.013; 0.097 | 0 | 0.375 |
|  | SD |  |  | 0.020 | 0.011; 0.039 | 0 |  |
| Fistula | HD | 4 | 588 | 0.047 | 0.013; 0.149 | 66.6 | 0.823 |
|  | SD |  |  | 0.055 | 0.024; 0.121 | 63.9 |  |
| Stricture | HD | 2 | 259 | 0.067 | 0.035; 0.125 | 2.7 | 0.412 |
|  | SD |  |  | 0.043 | 0.018; 0.099 | 0 |  |
| RP | HD | 7 | 688 | 0.227 | 0.145; 0.339 | 75.9 | 0.011 |
|  | SD |  |  | 0.079 | 0.039; 0.156 | 58.3 |  |
| HD=high dose; SD=standard dose; PR=partial response; CR=complete response; LR=locoregional; RP=radiation pneumonitis; CI=confidence interval | | | | | | | |

| **Supplementary Table S18. Subgroup meta-analysis of radiation techniques regarding any-grade toxicities in HD.** | | | | | | | |
| --- | --- | --- | --- | --- | --- | --- | --- |
|  | **Subgroup** | **No. of studies** | **No. of patients** | **Estimated proportion** | **95% CI** | **I^2^ (%)** | **P (test of subgroup difference)** |
| Anemia | IMRT/VMAT | 4 | 255 | 0.394 | 0.288; 0.512 | 93.3 | 0.138 |
|  | 2D/3D CRT |  |  | 0.842 | 0.256; 0.988 | NA |  |
| Leukopenia | IMRT/VMAT | 3 | 200 | 0.747 | 0.663; 0.834 | NA | 0.111 |
|  | 2D/3D CRT |  |  | 0.958 | 0.661; 0.996 | 68.2 |  |
| Neutropenia | IMRT/VMAT | 3 | 200 | 0.535 | 0.419; 0.647 | NA | <0.001 |
|  | 2D/3D CRT |  |  | 0.928 | 0.782; 0.979 | 63.1 |  |
| Thrombocytopenia | IMRT/VMAT | 8 | 890 | 0.264 | 0.150; 0.419 | 87.6 | 0.941 |
|  | 2D/3D CRT |  |  | 0.249 | 0.049; 0.681 | 94.1 |  |
| Fatigue | IMRT/VMAT | 2 | 126 | 0.451 | 0.339; 0.567 | NA | 0.966 |
|  | 2D/3D CRT |  |  | 0.455 | 0.329; 0.586 | NA |  |
| Dysphagia | IMRT/VMAT | 2 | 127 | 0.276 | 0.069; 0.661 | 87.7 | 0.301 |
|  | 2D/3D CRT |  |  | 0.115 | 0.038; 0.303 | NA |  |
| Esophagitis | IMRT/VMAT | 5 | 342 | 0.582 | 0.503; 0.656 | 0 | 0.722 |
|  | 2D/3D CRT |  |  | 0.804 | 0.038; 0.997 | 96.5 |  |
| Pneumonitis | IMRT/VMAT | 4 | 283 | 0.213 | 0.135; 0.319 | 55.3 | 0.383 |
|  | 2D/3D CRT |  |  | 0.385 | 0.094; 0.791 | 92.5 |  |
| HD=high dose; CI=confidence interval; IMRT=intensity modulated radiotherapy; VMAT=volumetric modulated radiotherapy; NA=not applicable due to only one study. | | | | | | | |

| **Supplementary Table S19. Meta-regression analysis of pneumonitis and OS.** | | | | |
| --- | --- | --- | --- | --- |
| **Any-grade** | | | | |
|  | | **95% CI** | |  |
| **Survival** | **Estimate** | **lower** | **upper** | **P value** |
| OS-1 year | 0.746 | -0.979 | 2.47 | 0.397 |
| OS-2 year | -0.063 | -1.19 | 1.070 | 0.913 |
| OS-3 year | 0.241 | -1.22 | 1.705 | 0.747 |
| OS-5 year | -1.58 | -6.23 | 3.06 | 0.504 |
| **≥grade 3** | | | | |
|  | | **95% CI** | |  |
| **Survival** | **Estimate** | **lower** | **upper** | **P value** |
| OS-1 year | 3.75 | -5.61 | 13.11 | 0.433 |
| OS-2 year | 1.91 | -8.92 | 12.7 | 0.729 |
| OS-3 year | 9.12 | -11.4 | 29.6 | 0.383 |
| OS=overall survival; CI=confidence interval | | | | |

| **Supplementary Table S20. Differences of chemotherapy usage between regions.** | | | |
| --- | --- | --- | --- |
|  | **Region** | |  |
| **Agent** | **Asia** | **Western** | **P value** |
| Taxane | 10 | 4 | 0.746 |
| Platinum | 24 | 15 | 0.738 |
| 5-FU | 18 | 10 | 1 |
| Cetuximab | 1 | 4 | 0.171 |
| 5-FU=5-fluorouracil | | | |

| **Supplementary Table S21. Change of out-of-bag R^2^ between full model and treatment-model.** | | | | | | | | |
| --- | --- | --- | --- | --- | --- | --- | --- | --- |
|  | **Age** | **Sex** | **ECOG** | **Tumor length** | **SqCC** | **Middle/lower** | **T3/4** | **N+** |
| OS-1year | NS | NS | NS | NS | NS | NS | NS | NS |
| OS-2year | NS | NS | NS | NS | 5.9% (P=0.3819) | NS | NS | NS |
| OS-3year | NS | NS | NS | 1.7% (P= 0.4407) | 4.6% (P=0.7307) | NS | NS | NS |
| OS-5year | NS | NS | NS | NS | 13.8% (P=0.432) | NS | NS | NS |
| PFS-1year | NS | NS | NS | NS | 9.4% (P=0.397) | 14.7% (P=0.9848) | NS | NS |
| PFS-2year | NS | NS | NS | NS | NS | 21.7% (P=0.2403) | NS | NS |
| PFS-3year | NS | NS | NS | NS | NS | NS | NS | NS |
| PFS-5year | NS | NS | NS | NS | NS | NS | NS | NS |
| LRPFS-1year | NS | 4.7% (P=0.6811) | NS | NS | NS | -8% | -0.11% | NS |
| LRPFS-2year | NS | NS | NS | NS | NS | NS | NS | NS |
| LRPFS-3year | NS | NS | NS | NS | NS | NS | NS | NS |
| LRPFS-5year | NS | NS | NS | NS | NS | NS | NS | NS |
| OS=overall survival; PFS=progression-free year; LRPFS=locoregional progression-free survival; ECOG= Eastern Cooperative Oncology Group; SqCC=squamous cell carcinoma; NS: not significant in the meta-regression analysis. P values were calculated by paired T test. No T test was performed if there was negative benefit of the full model. | | | | | | | | |

**Supplementary Figure S1. Risk of bias of included randomized controlled trials.**


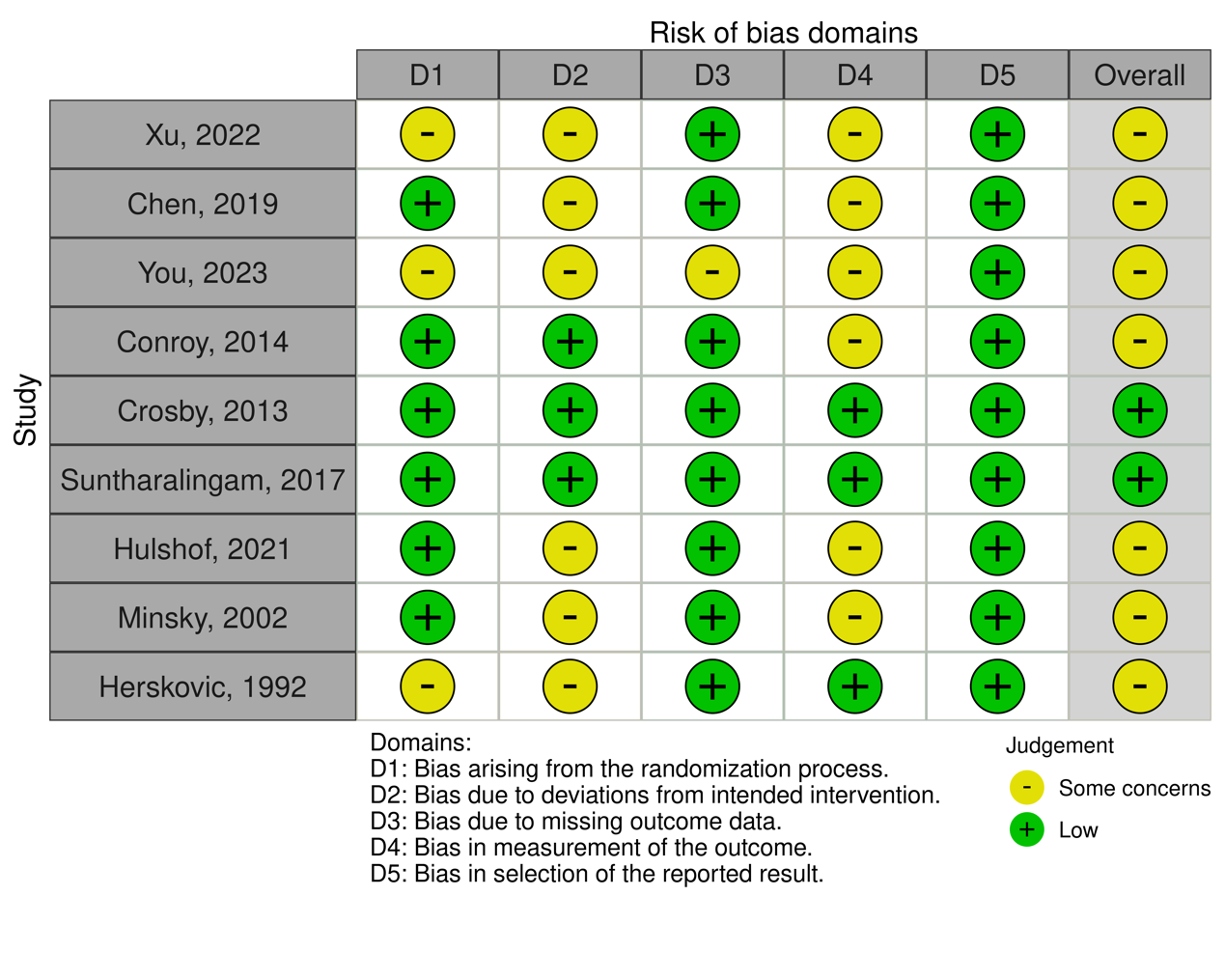


**Supplementary Figure S2. Meta-regression analyses for survival outcomes.**

**Supplementary Figure S3. Posterior distributions of the log-odds ratios for overall survival in the high-dose group in middle/lower-high studies.**

**
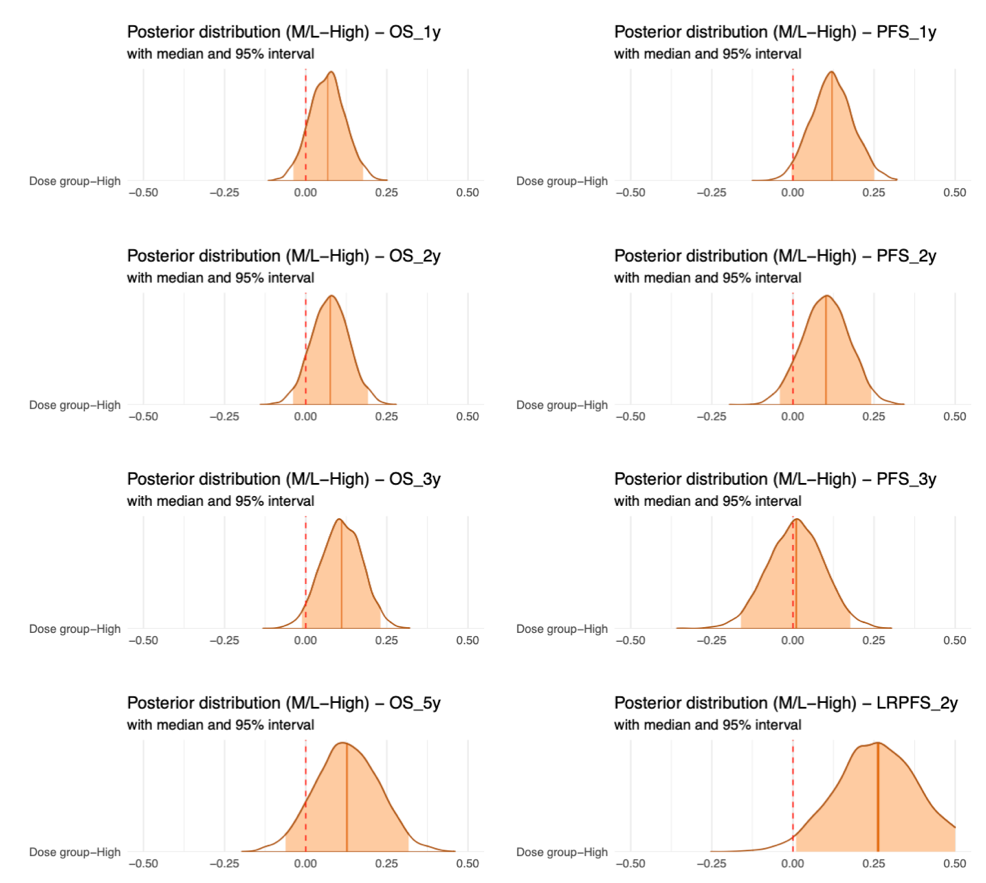
**

**Supplementary Figure S4. Posterior distributions of the log-odds ratios for survival outcomes in high-dose group in studies using modern radiation techniques.**

**
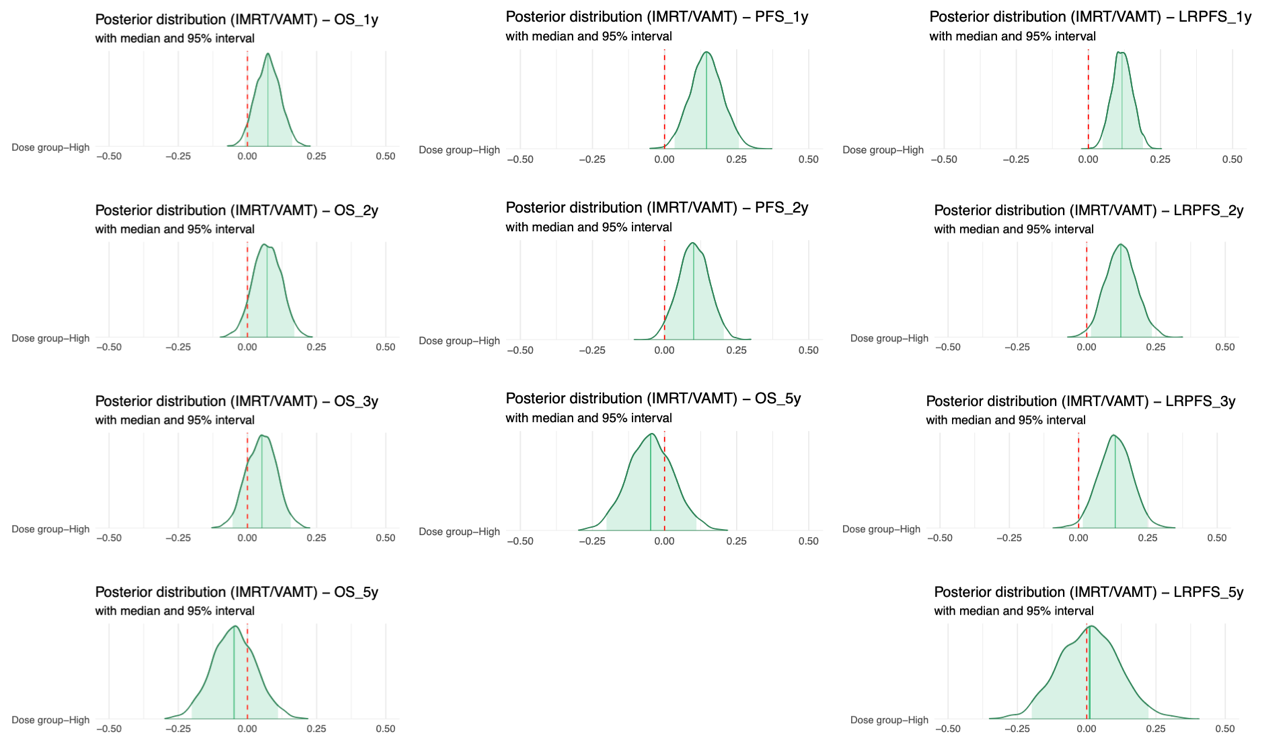
**

**Supplementary Figure S5. Differences of clinical factors between Asia and Western countries with high proportion of SqCC in the HD subgroup. SqCC=squamous cell carcinoma; HD=high dose; ECOG PS=Eastern Cooperative Oncology Group (ECOG) performance status; N=lymph node metastasis**

**Supplementary Figure S6. Importance plots for PFS in MetaForest analysis. PFS=progression-free survival.**

**
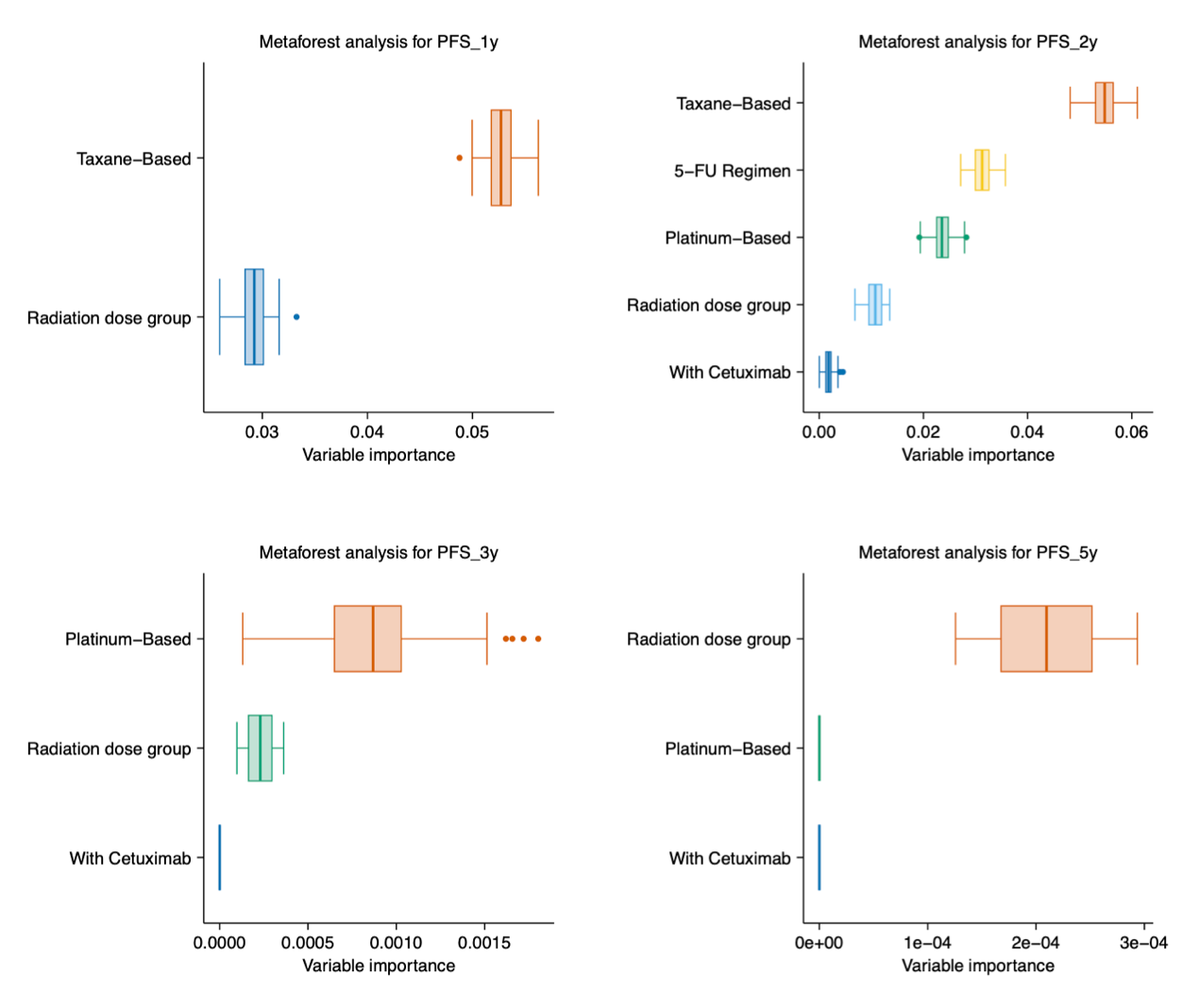
**

**Supplementary Figure S7. Partial dependence plots for PFS. PFS=progression-free survival. 5-FU=5-fluorouracil. Cetux=Cetuximab.**

**
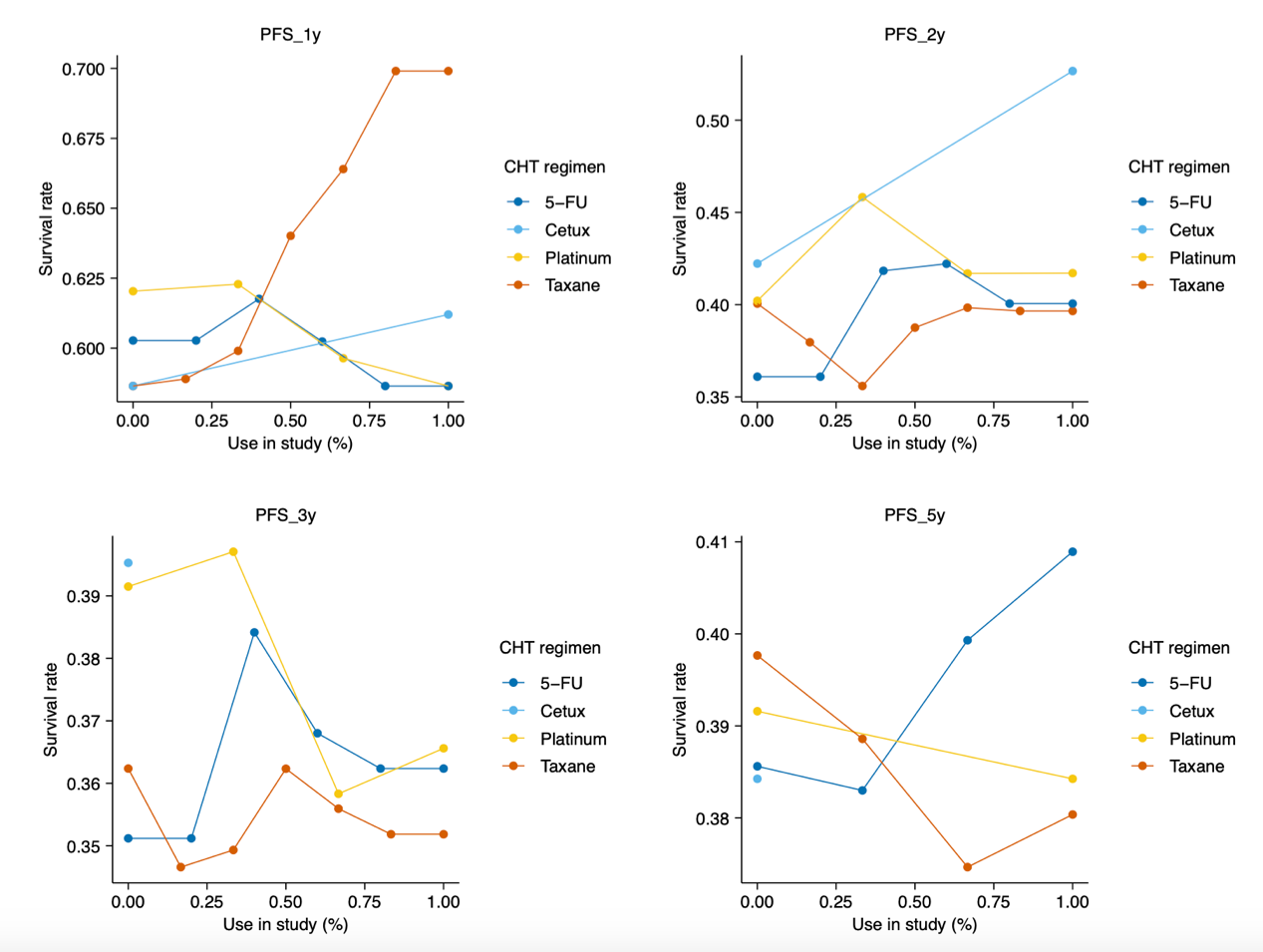
**

**Supplementary Figure S8. Importance plots for LRPFS in MetaForest analysis. LRPFS=Locoregional progression-free survival.**

**
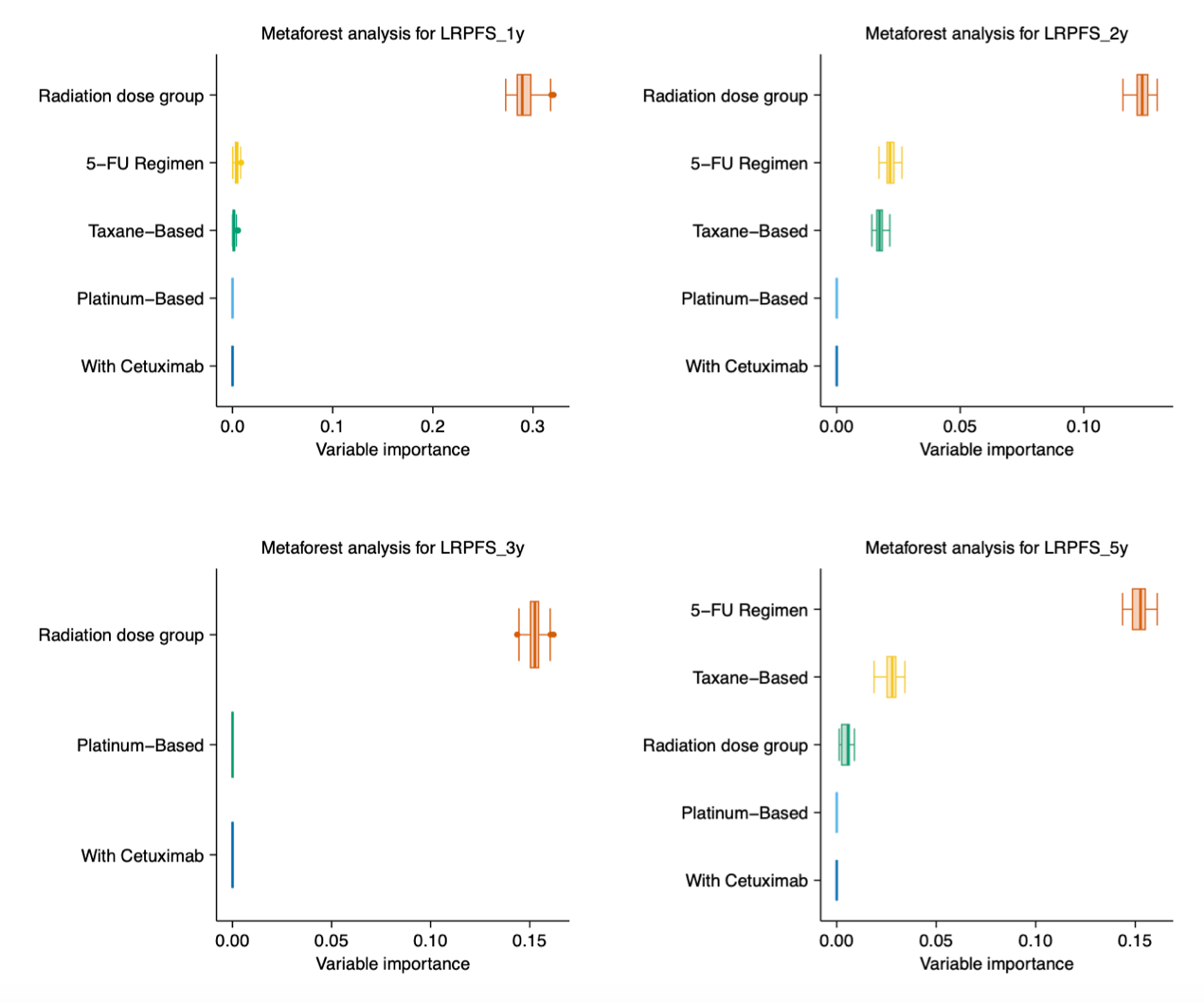
**

**Supplementary Figure S9. Partial dependence plots for LRPFS. LRPFS=Locoregional progression-free survival. 5-FU=5-fluorouracil. Cetux=Cetuximab.**

**
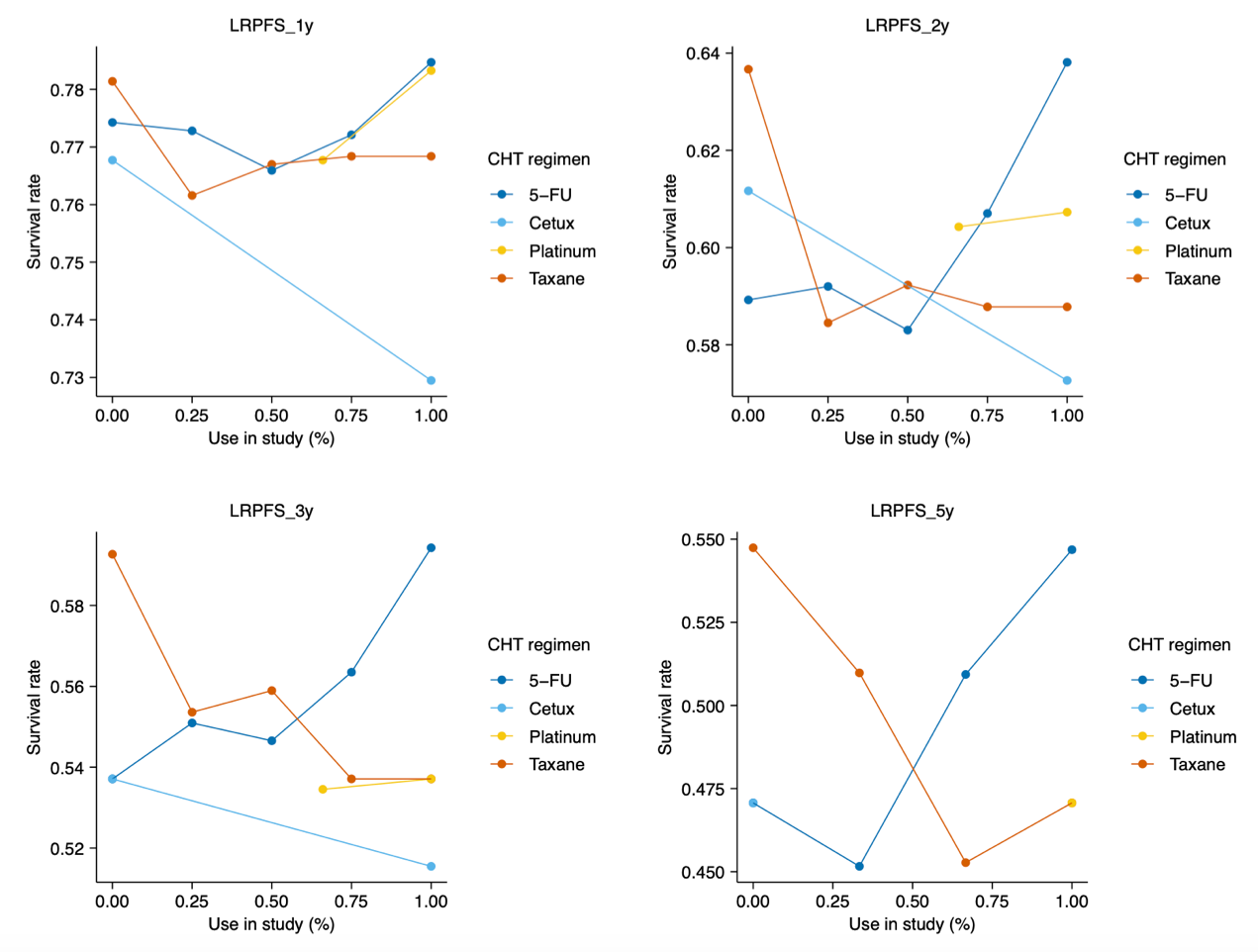
**

**Supplementary Figure S10. Partial dependence plots for Dose groups.**

**
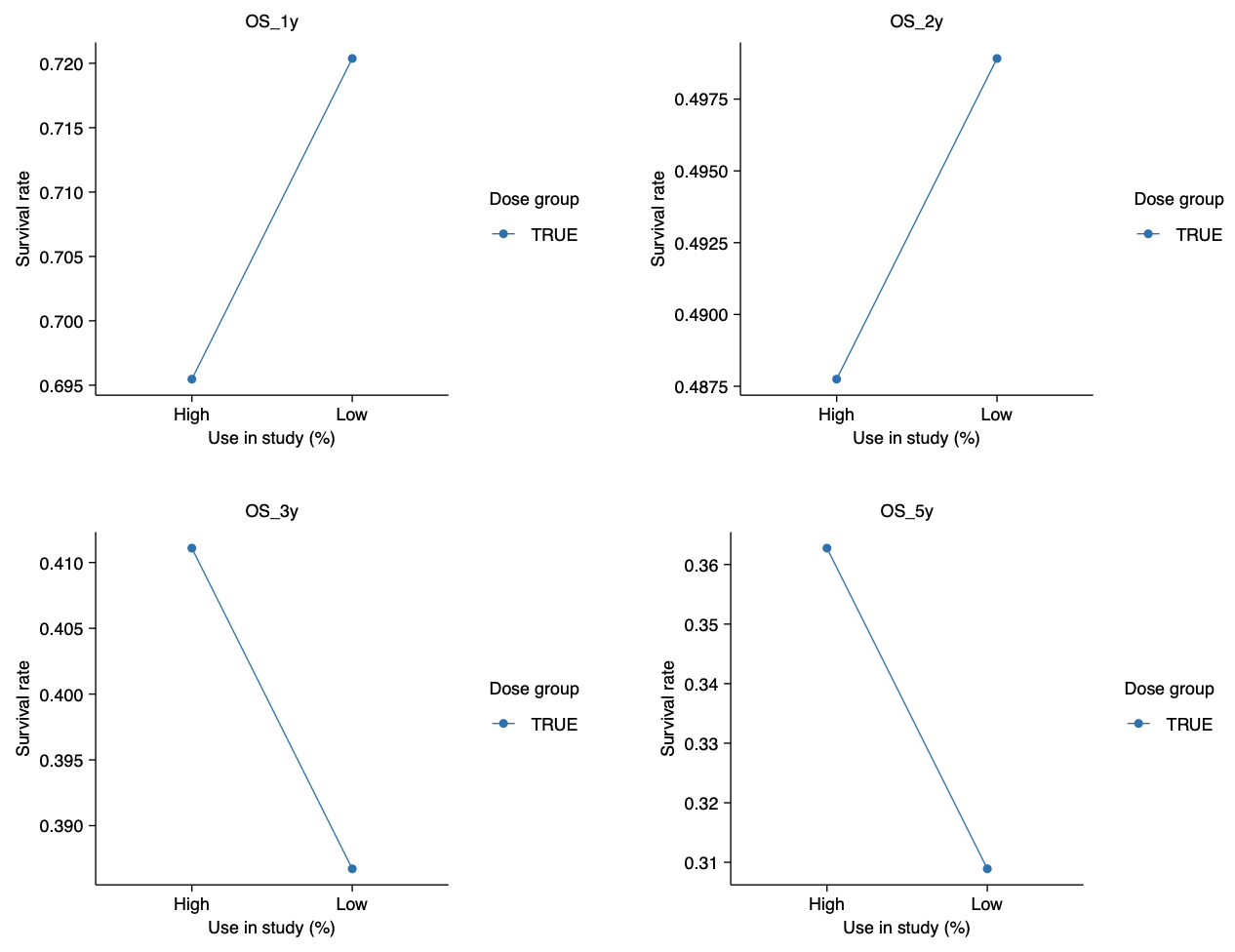

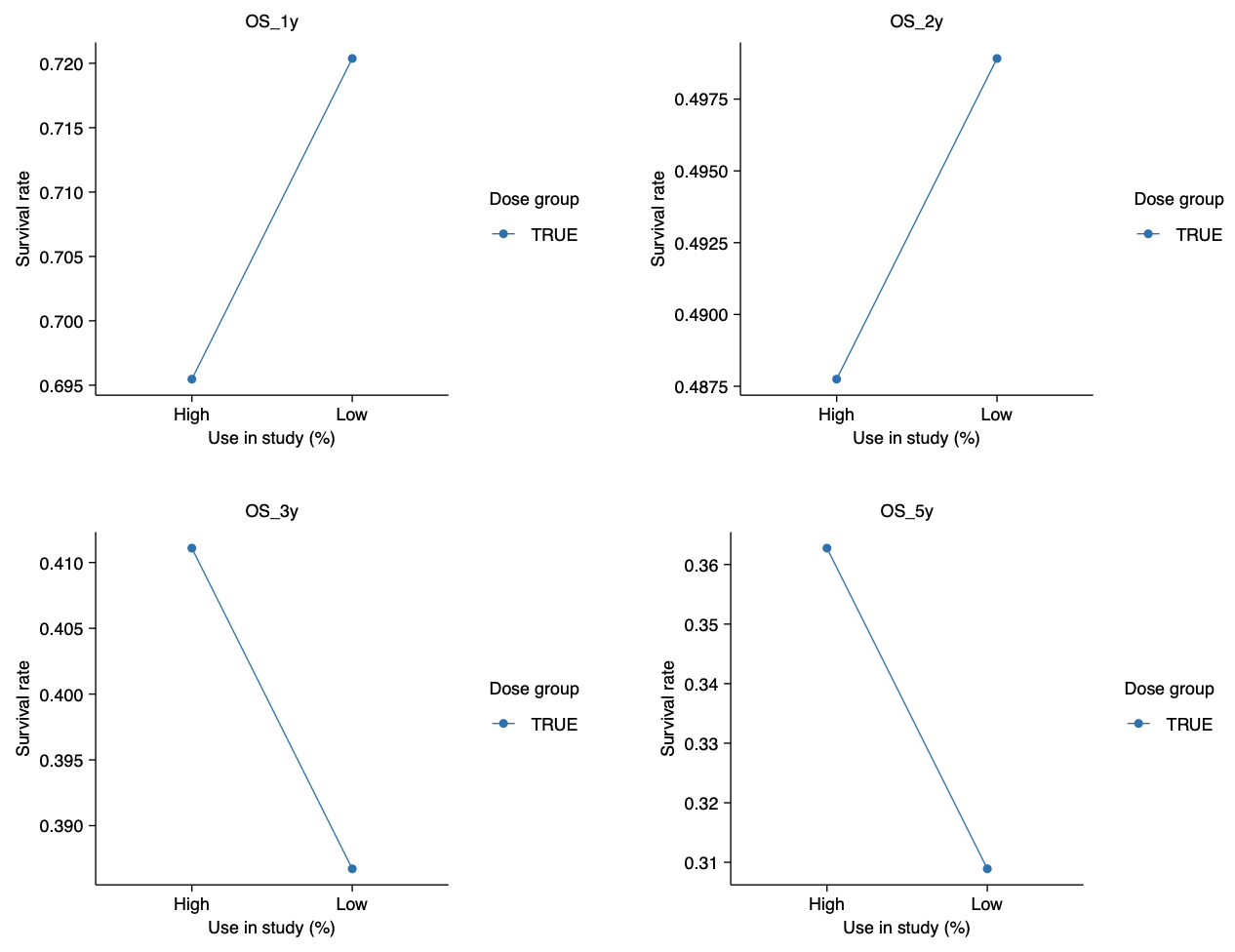
**

**
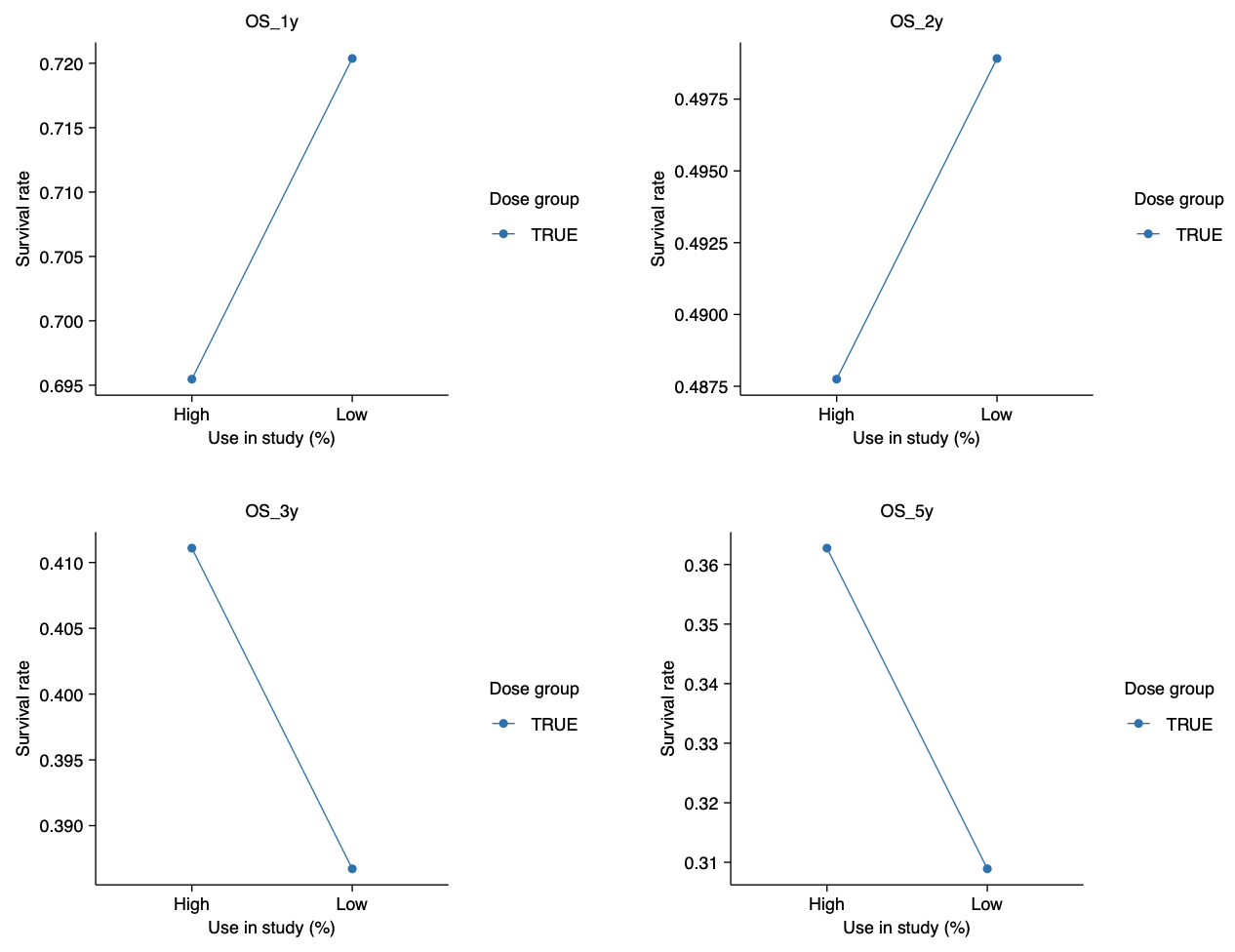

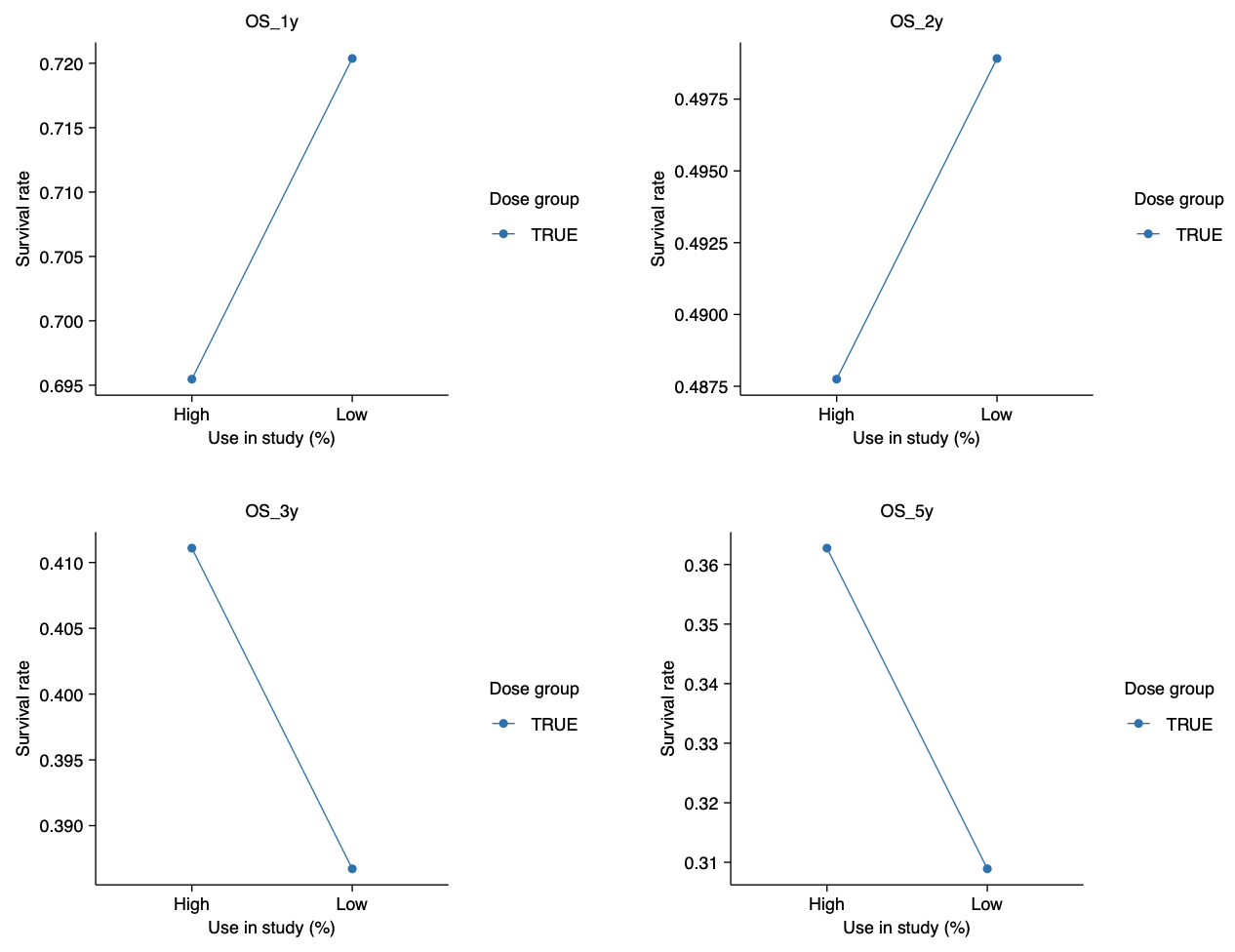
**

**
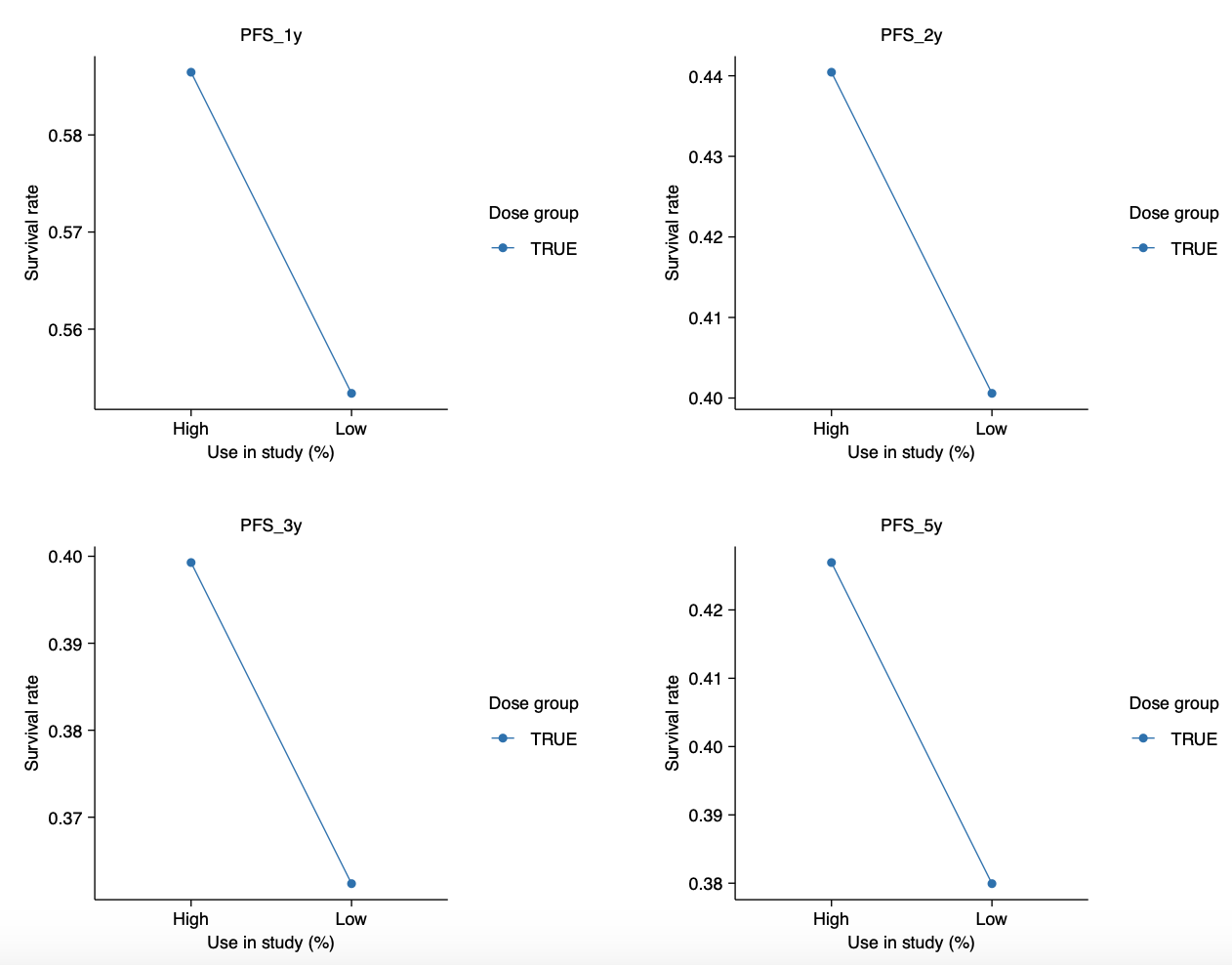

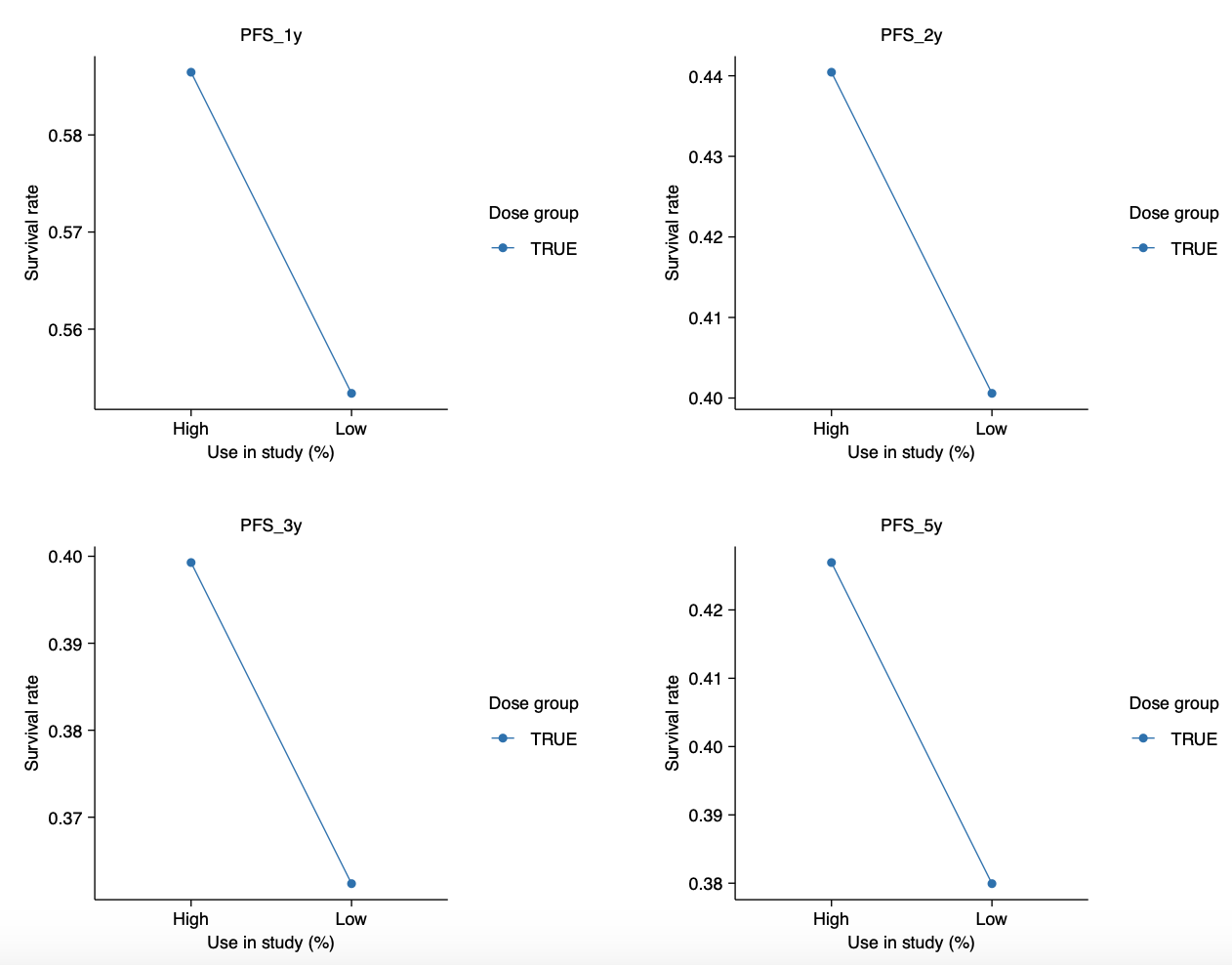
**

**
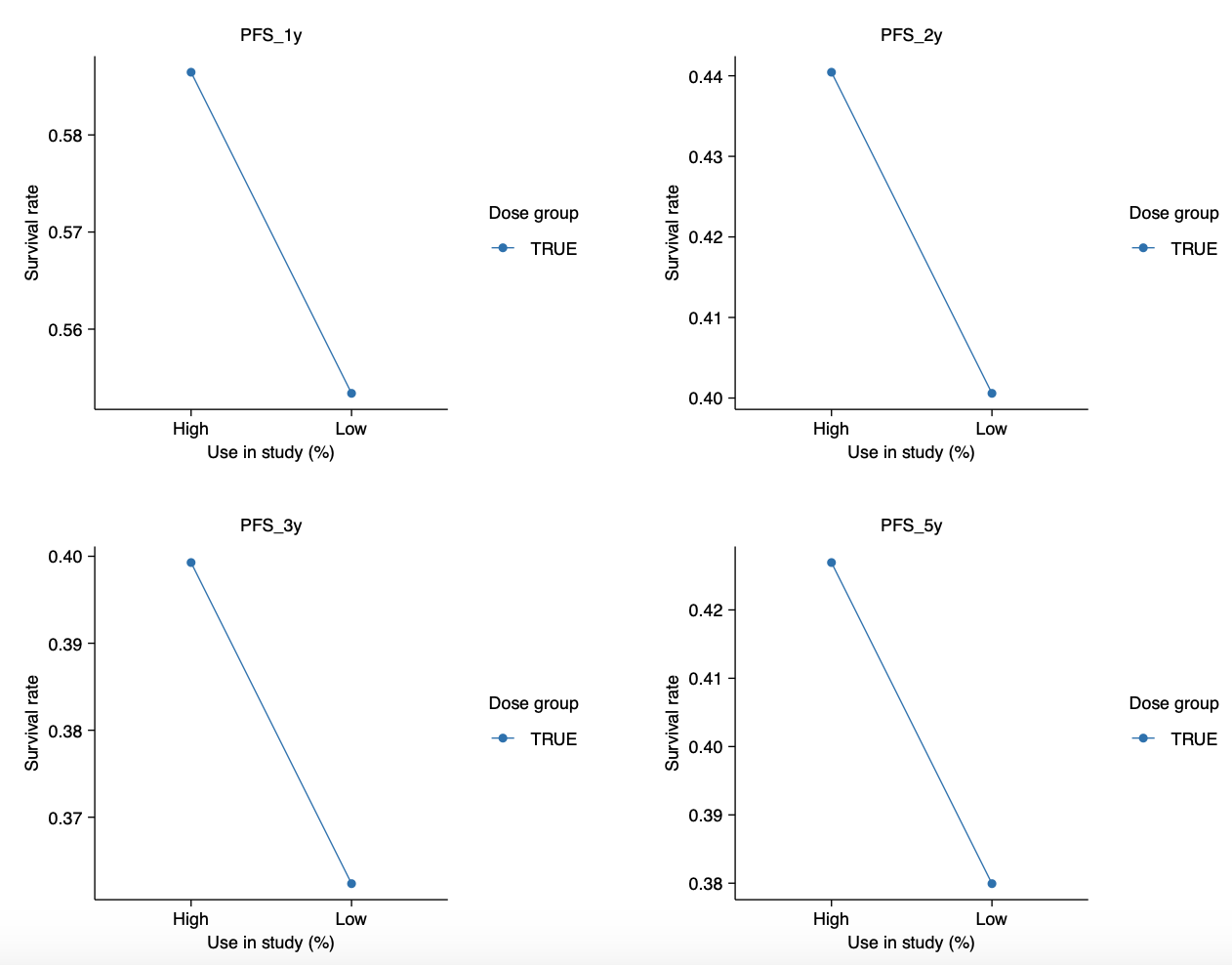

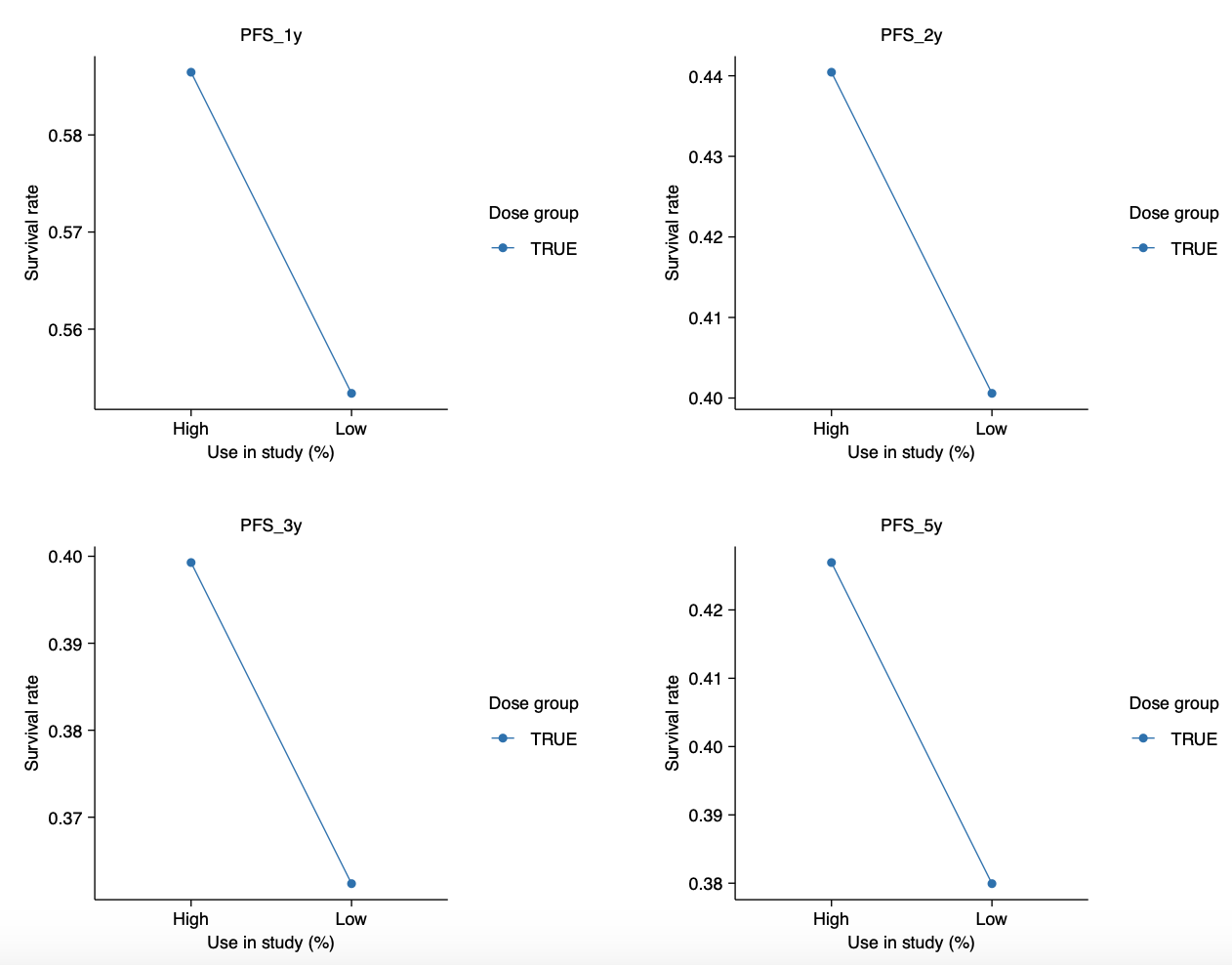
**

**
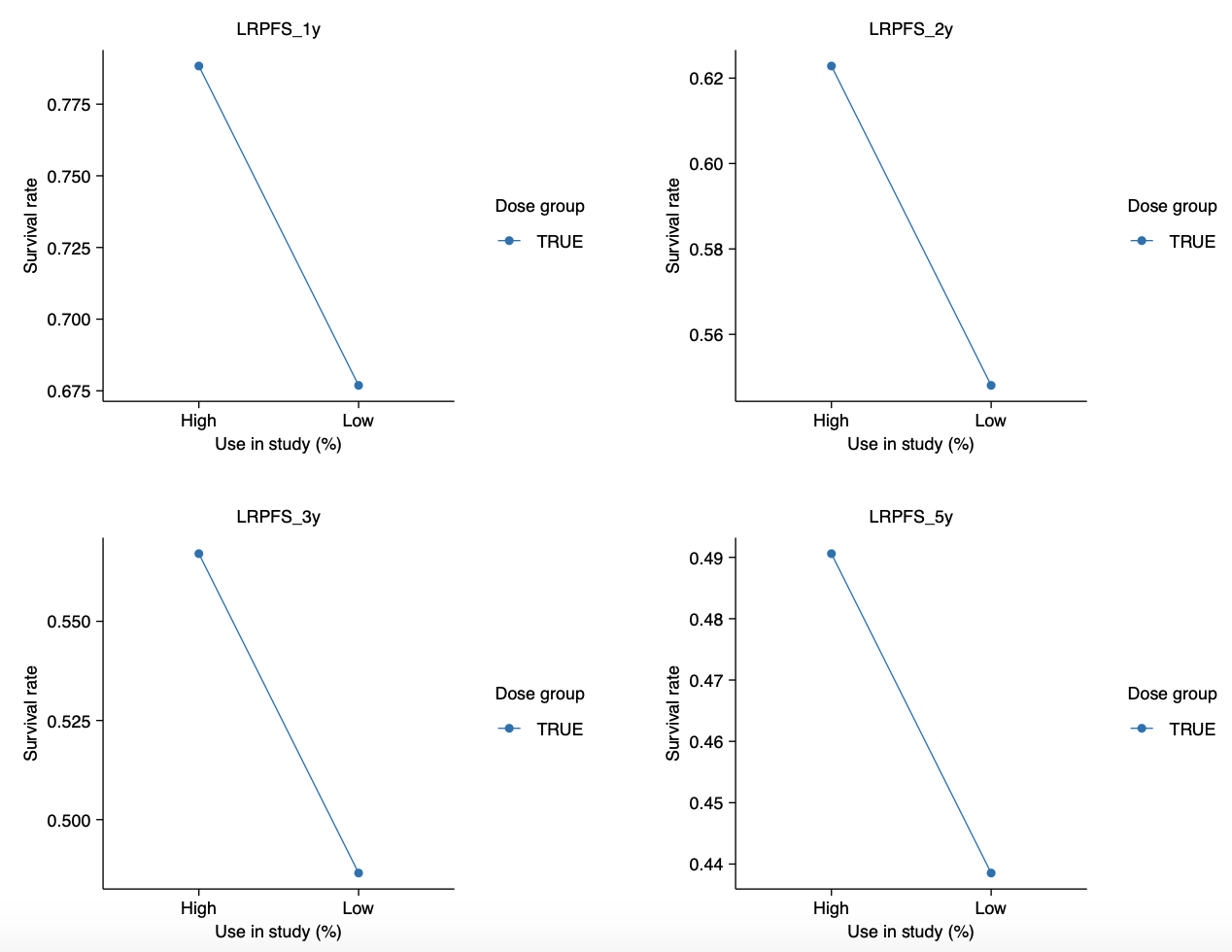

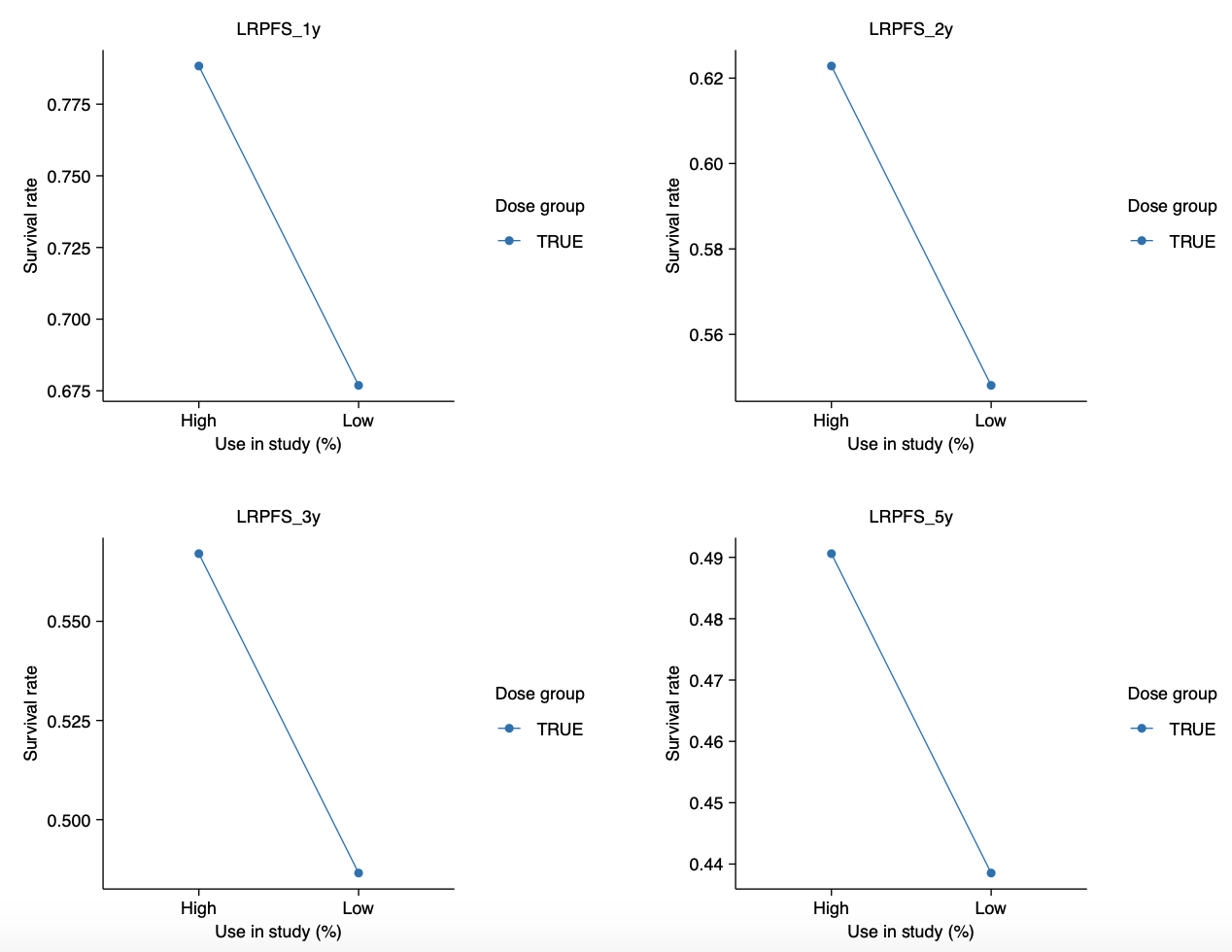
**

**
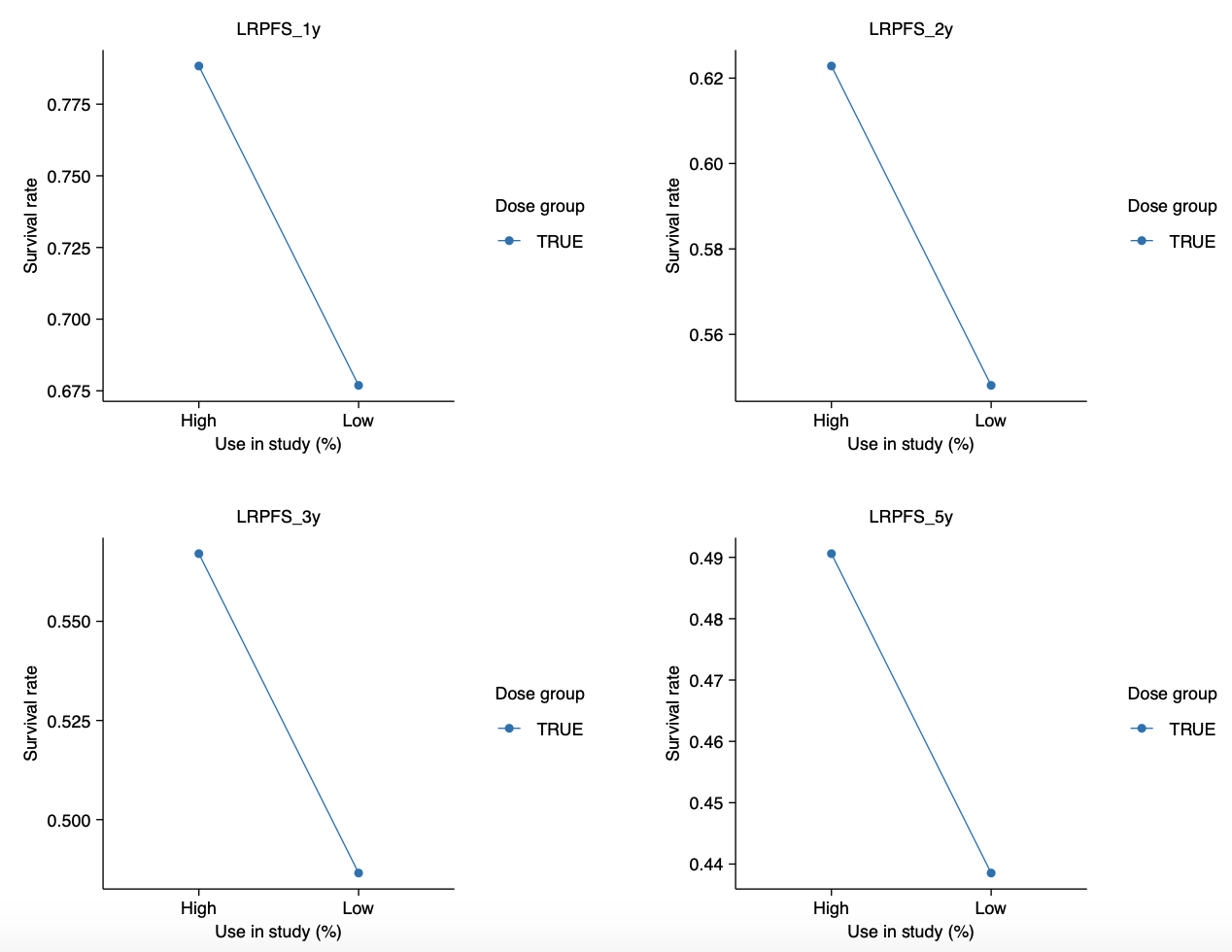

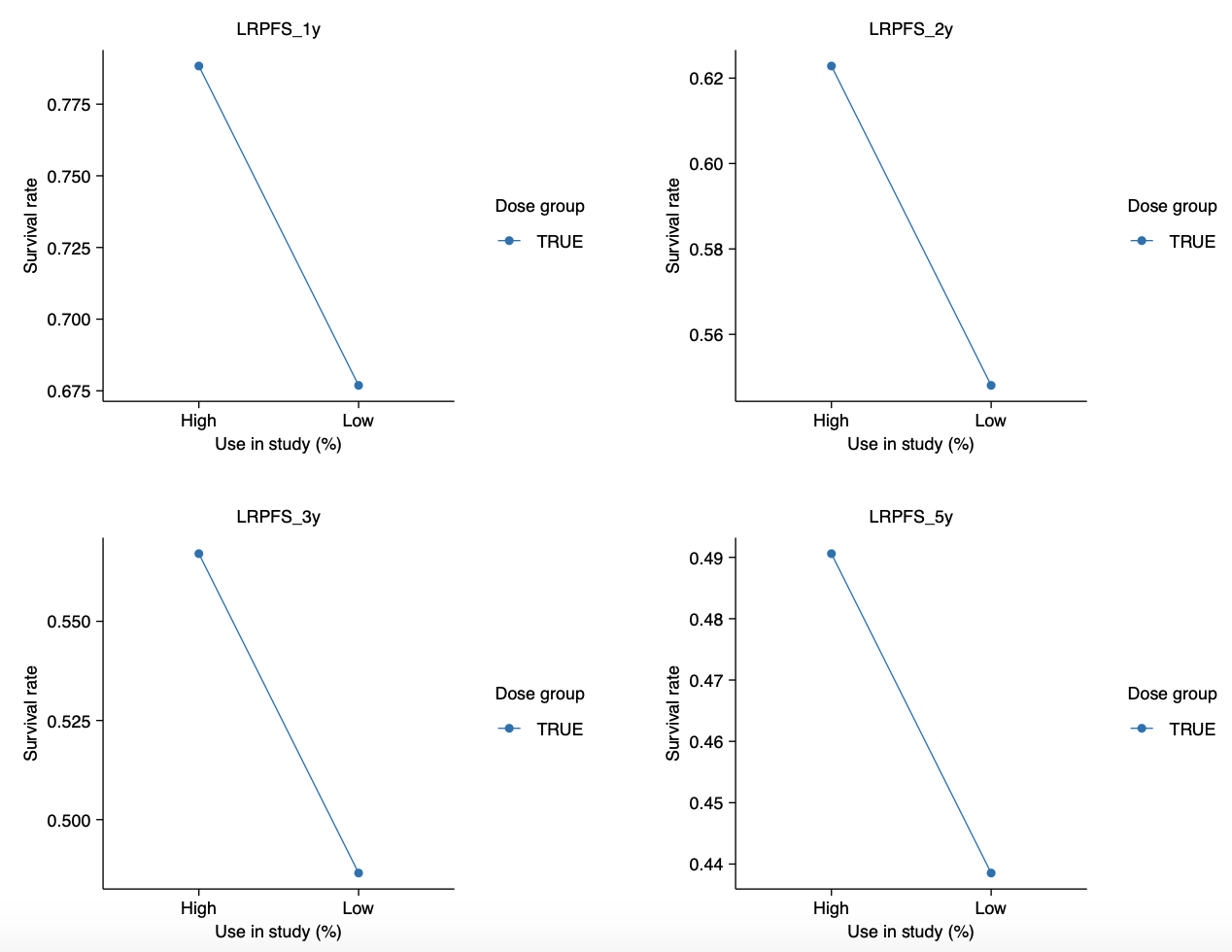
**

**Supplementary Figure S11. Importance plots for OS in Asian population. OS=overall survival.**

**
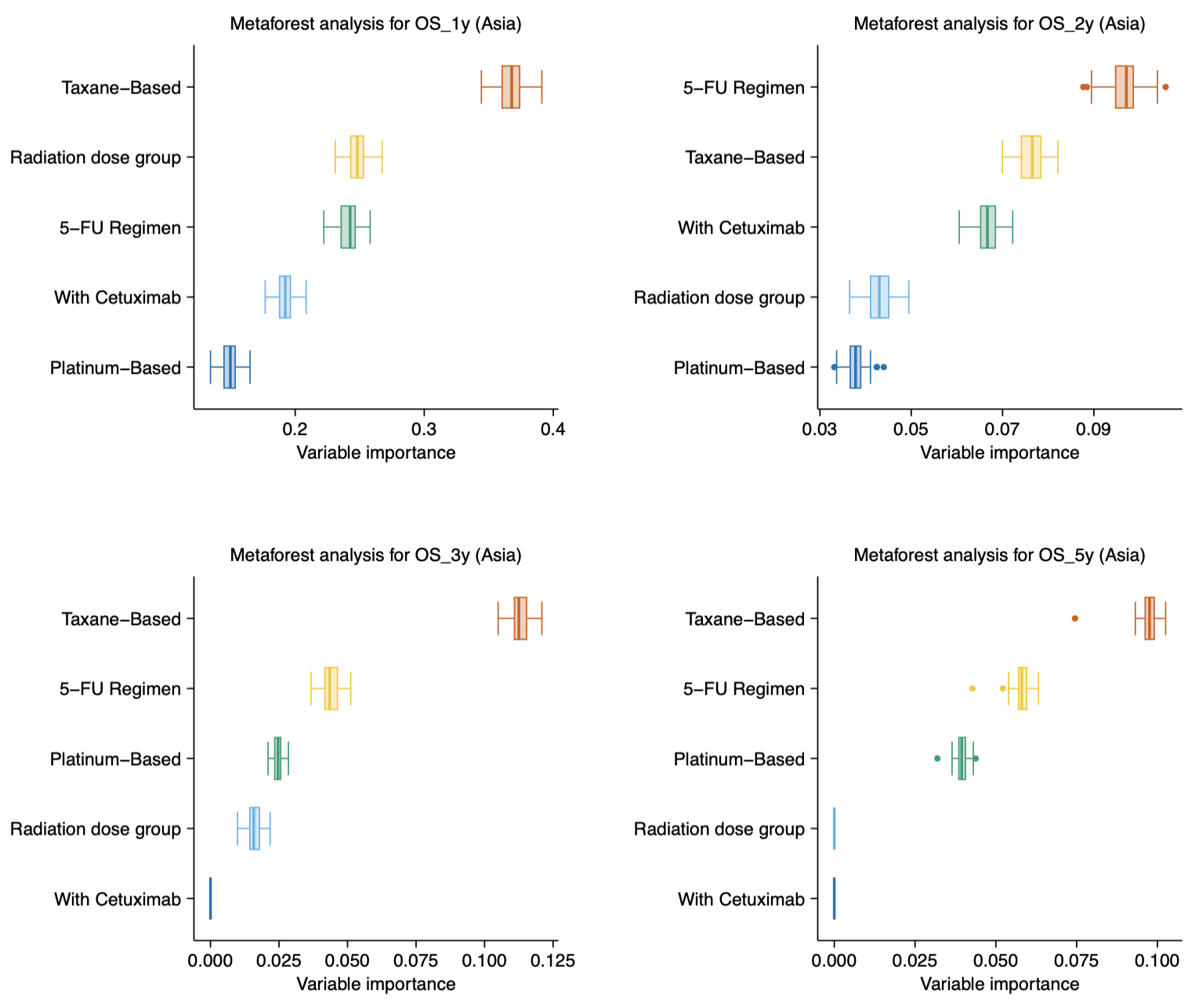
**

**Supplementary Figure S12. Partial dependence plots for OS in Asian population. OS=overall survival. 5-FU=5-fluorouracil. Cetux=Cetuximab.**

**
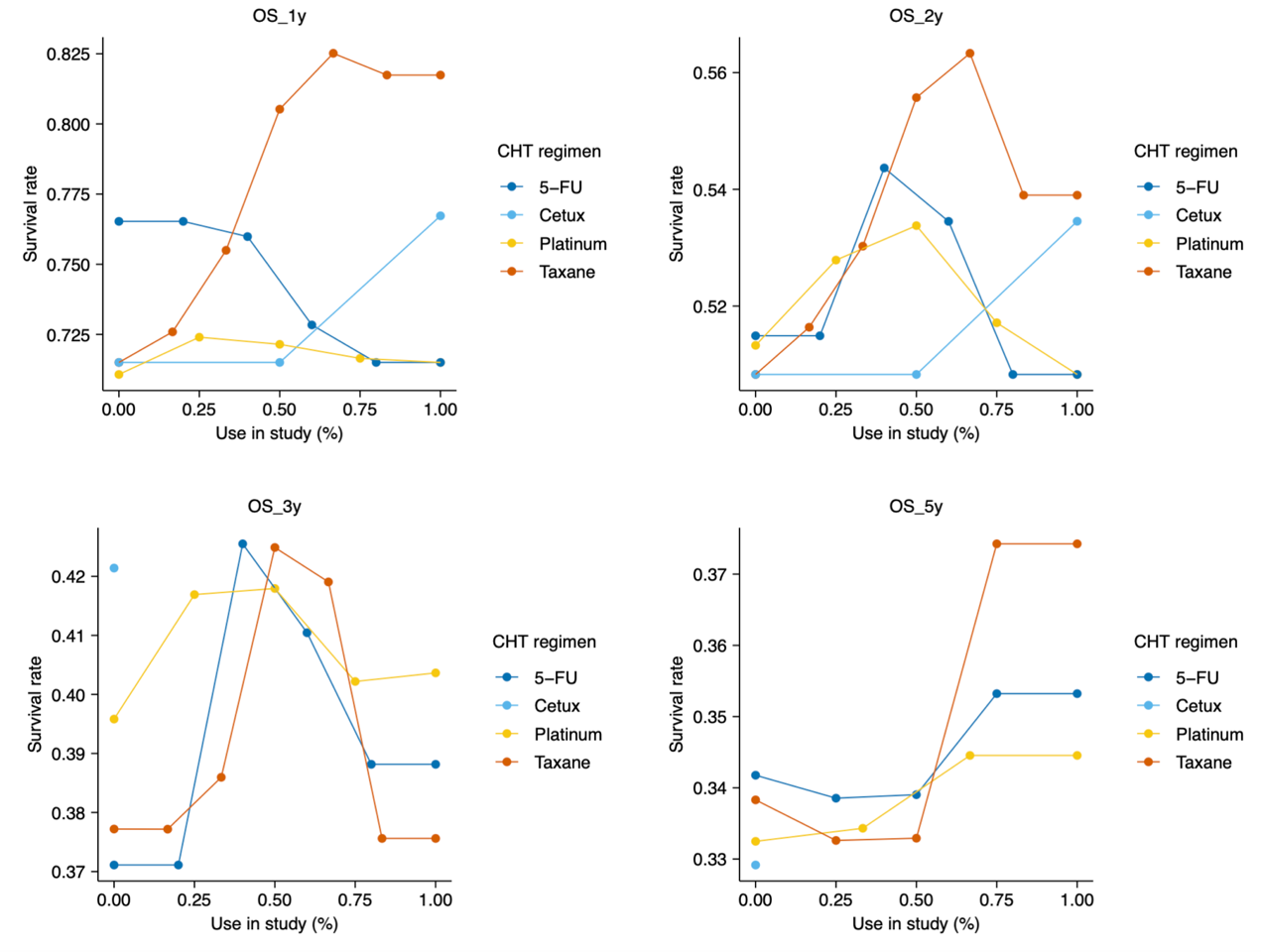
**

**Supplementary Figure S13. Importance plots for PFS and LRPFS in Asian population. PFS=progression-free survival. LRPFS=locoregional progression-free survival.**

**
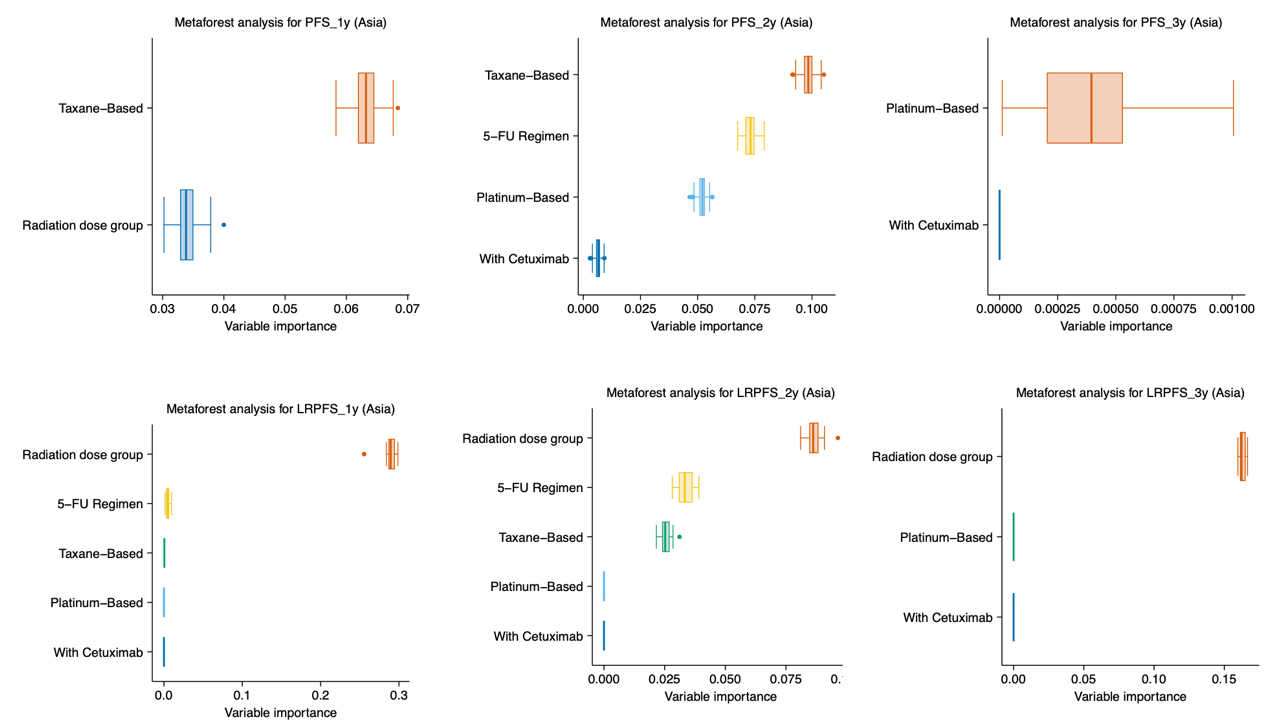
**

**Supplementary Figure S14. Partial dependence plots for PFS and LRPFS in Asian population. PFS=progression-free survival. LRPFS=locoregional progression-free survival. 5-FU=5-fluorouracil. Cetux=Cetuximab.**

**
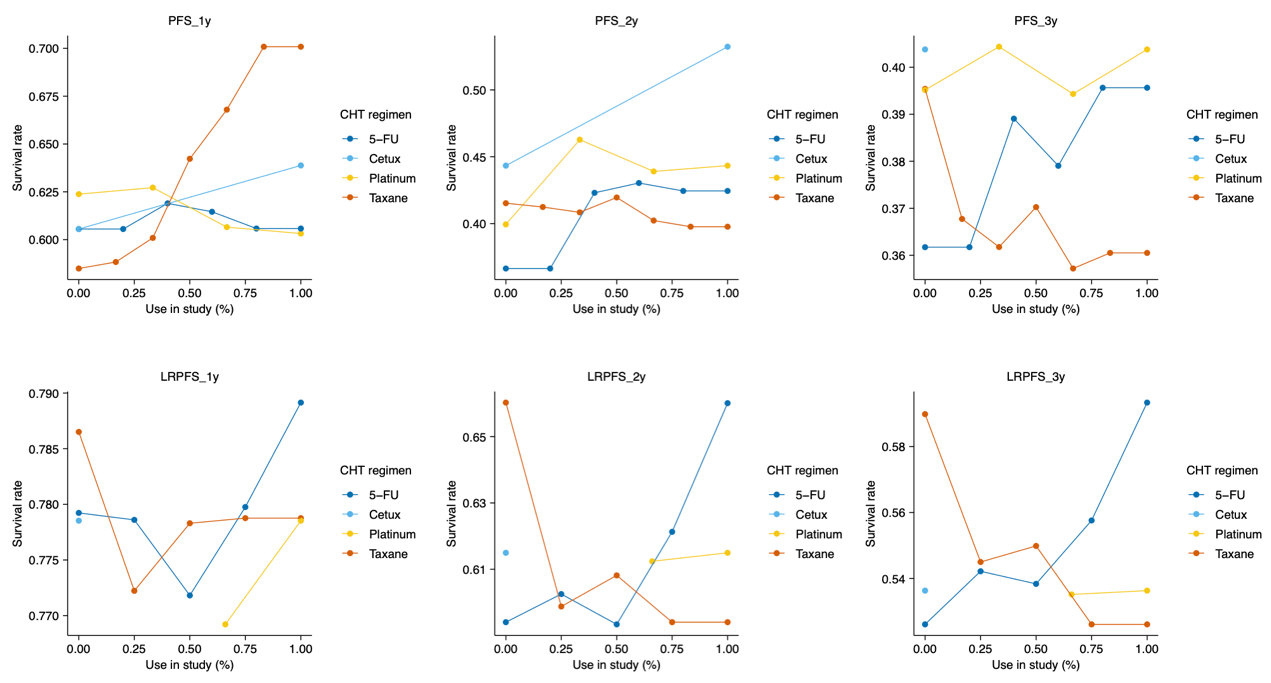
**

**Supplementary Figure S15. Publication bias.**


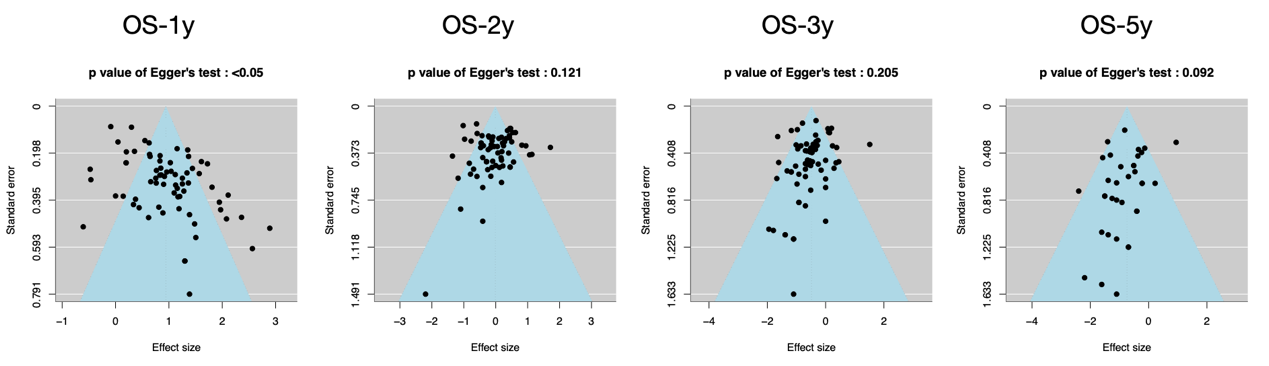


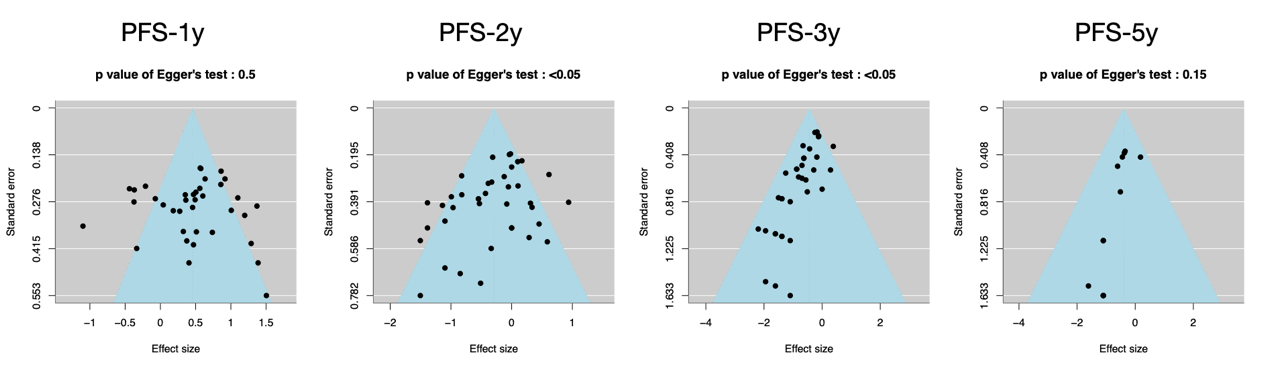


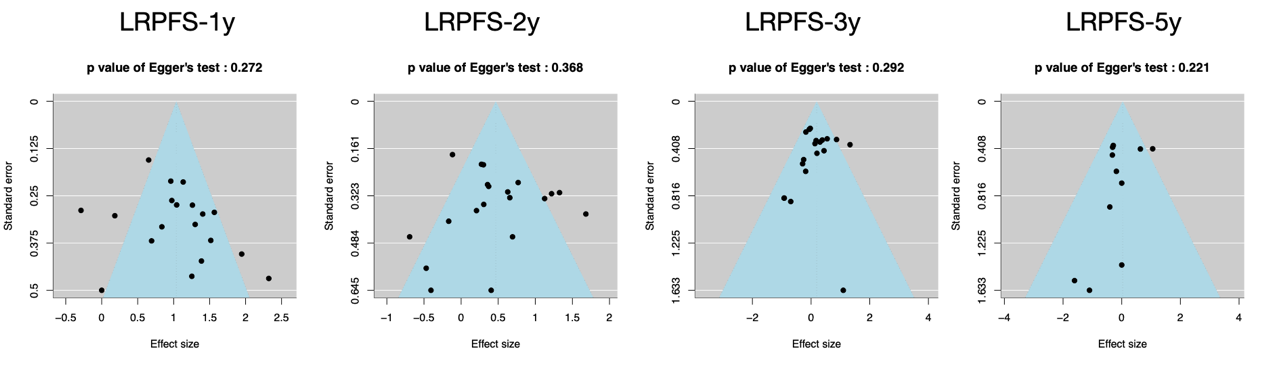


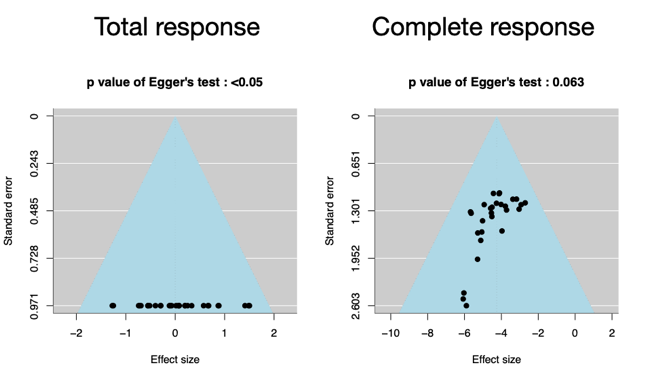


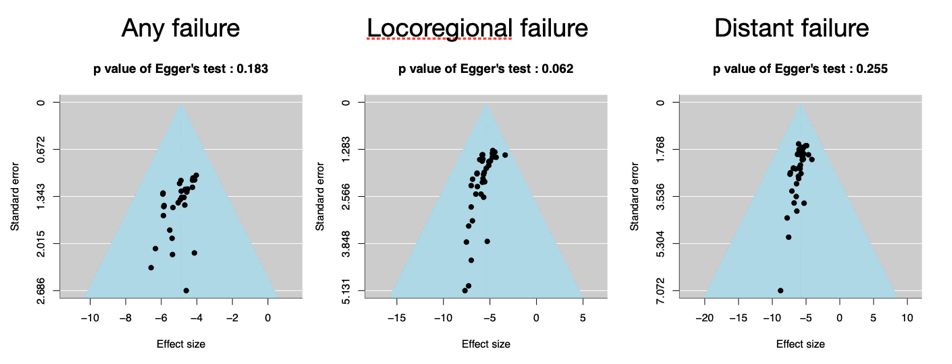


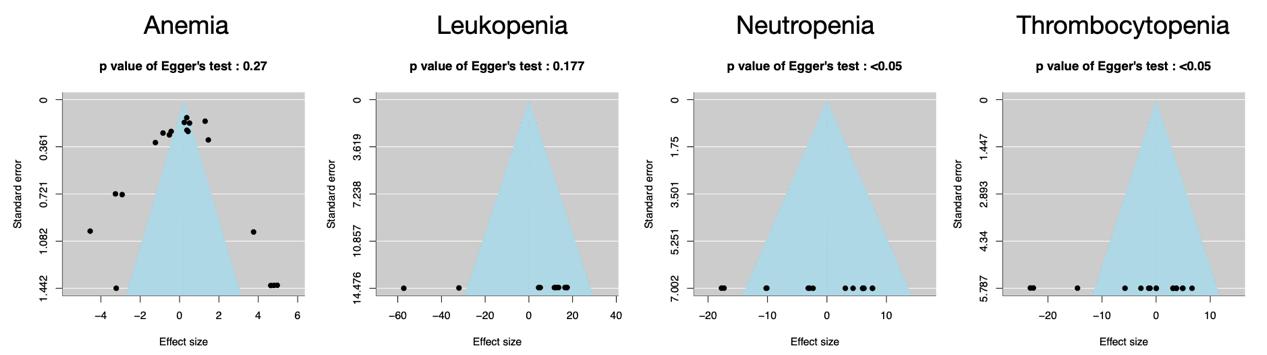


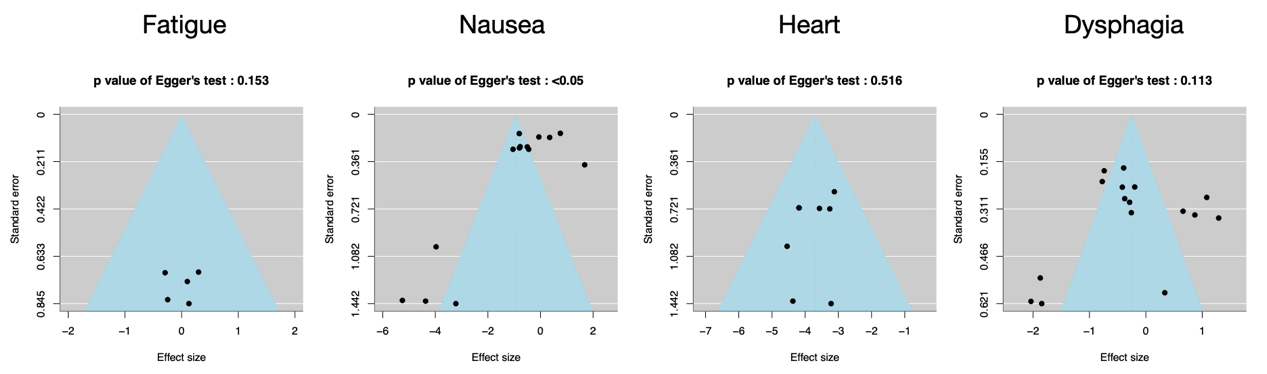


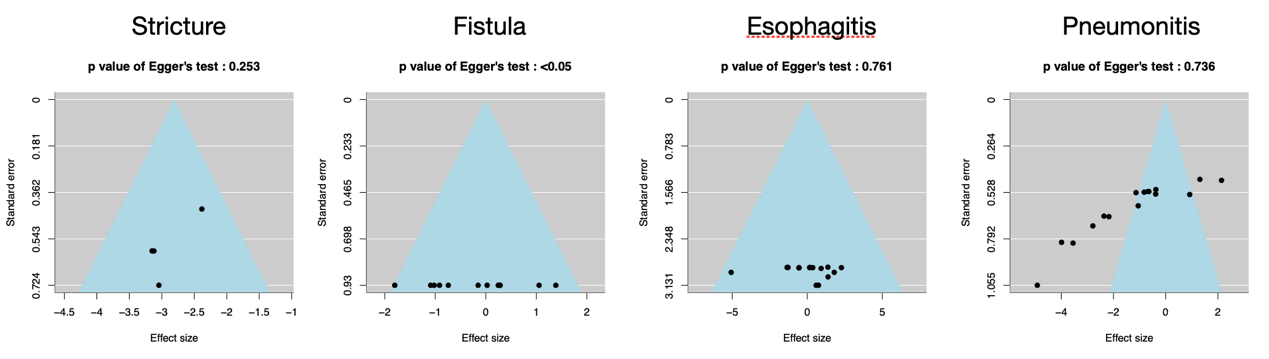


**Supplementary Figure S16. Sensitivity analysis using leave-one-out.**
